# The application of rhubarb concoctions in traditional Chinese medicine and its compounds, processing methods, pharmacology, toxicology and clinical research

**DOI:** 10.3389/fphar.2024.1442297

**Published:** 2024-08-07

**Authors:** Yi Wen, Pei-Jia Yan, Pei-Xuan Fan, Shan-Shan Lu, Mao-Ya Li, Xian-Yun Fu, Shao-Bin Wei

**Affiliations:** ^1^ Gynecology Department, Hospital of Chengdu University of Traditional Chinese Medicine, Chengdu, China; ^2^ College of Medicine and Health Sciences, China Three Gorges University, Yichang, China

**Keywords:** clinical applications, compounds, pharmacology, processing, rhubarb

## Abstract

**Objective:**

This study reviews the development of rhubarb processing and the current status of pharmacological research. We summarized the effects of different processing methods on the active compounds, pharmacological effects, and toxicity of rhubarb, as well as the clinical application of different concoctions, providing reference for further pharmacological research and clinical application of rhubarb.

**Methods:**

A comprehensive literature review was conducted using databases such as Pubmed, Embase, National Science and Technology Library, Web of science, CNKI, China Science and Technology Journal Database, SinoMed, and the *Pharmacopoeia of the People*’*s Republic of China*. Search terms included “rhubarb”, “raw rhubarb”, “wine rhubarb”, “cooked rhubarb”, “rhubarb charcoal”, “herbal processing”, “compounds”, “pharmacological effects”, “inflammation”, “gastrointestinal bleeding”, and “tumor”.

**Results:**

Historical records of rhubarb processing date back to the Han Dynasty, with continual innovations. Currently, the types of rhubarb used in traditional Chinese medicine have stabilized to three species: *Rheum palmatum L.*, *Rheum tanguticum Maxim.ex Balf.* and *Rheum officinale Baill*. Common concoctions include raw rhubarb, wine rhubarb, cooked rhubarb and rhubarb charcoal. The active compounds of rhubarb are known to defecation, exhibit antibacterial and anti-inflammatory properties, regulate coagulation, protect the digestive system, and possess anti-tumor activities. Guided by Chinese medicine theory, the use of different rhubarb concoctions can enhance specific effects such as purgation to eliminate accumulation, clearing heat and toxins, cooling blood to stop hemorrhages, activating blood circulation to remove blood stasis, and inducing dampness to descend jaundice, thereby effectively treating various diseases. The therapeutic impact of these concoctions on diseases reflects not only in the changes to the active compounds of rhubarb but also in the formulations of traditional Chinese medicine. Processing has also shown advantages in reducing toxicity.

**Conclusion:**

Different processing methods alter the active compounds of rhubarb, thereby enhancing its various pharmacological effects and meeting the therapeutic needs of diverse diseases. Selecting an appropriate processing method based on the patient’s specific conditions can maximize its pharmacological properties and improve clinical outcomes.

## 1 Introduction

Rhubarb, an herbaceous plant within the Polygonaceae family, comprises approximately 60 species globally (Cao et al., 2017). In China, it is primarily utilized for medicinal purposes, ranking as one of the four principal traditional Chinese medicines (Min et al., 2019). Rhubarb’s roots and stems are therapeutically effective, with its medicinal application initially recorded in the *Shennong Herbal Scripture* (*Shennong Bencao Jing*). The species of rhubarb commonly employed in traditional Chinese medicine (TCM) are the dried roots and rhizomes of *Rheum palmatum L.*, *Rheum tanguticum Maxim. ex Balf.* and *Rheum officinale Baill*. These exhibit multiple pharmacological properties, including purgation, heat clearing, fire discharging, blood cooling and detoxification, stasis elimination, collateral obstruction removal and jaundice reduction (Committee, 2020). *Rheum palmatum L*. is mainly produced in Gansu and Qinghai, and is mostly cultivated, accounting for the majority of rhubarb production in China. *Rheum tanguticum Maxim. ex Balf.* is mainly produced in Qinghai and Gansu, wild or cultivated. *Rheum officinale Baill*. is mainly produced in Sichuan and Guizhou, cultivated or wild, with relatively low yield (Kang, 2013). As a multi origin traditional Chinese medicine, research has shown that there are differences in the types and contents of compounds contained in rhubarb medicinal materials with different origins, which may lead to differences in their pharmacological effects. However, such differences have not been considered in practical clinical applications.

The processing of Chinese medicines adheres to traditional theories, guided by the requirements of diagnosis, drug characteristics, and the specifics of pharmaceutical preparation (Zhang, 2009). The processing of rhubarb has a long history. Currently, the main rhubarb concoctions used in China include raw rhubarb, wine rhubarb, cooked rhubarb and rhubarb charcoal, addressing ailments across the cardiovascular (Wu et al., 2023), digestive (Hu et al., 2018), endocrine (Zeng et al., 2021), reproductive systems (Otoo et al., 2023) and more.

This study examines the alterations in active compounds of three rhubarb species listed in the *Pharmacopoeia of the People*’*s Republic of China* after different processing methods, their impacts on traditional and modern pharmacology, clinical uses, and toxicity, providing reference for further pharmacological and clinical research on rhubarb in the future.

## 2 Processing of rhubarb

“Using decoction pieces from processed herbs for treatment, raw and cooked herbs have different medicinal effects” is a characteristic of TCM (Zhu et al., 2024). The processing of Chinese herbal medicine involves various procedures such as washing, cutting, soaking, boiling, stir-frying, roasting and steaming, to produce clinically applicable decoction pieces. During these processes, the addition of auxiliary ingredients like vinegar, wine, honey and salt water is often required (Zhu et al., 2016a). In clinical settings, it is imperative to consider numerous factors, including the balance of yin and yang, the state of the viscera, qi and blood, dietary habits, emotional states, climatic conditions and the daily activities of patients to ensure precise diagnosis and treatment. Thus, it is essential to modify the properties, flavors and meridians of TCM herbs through varied processing techniques to optimize efficacy, minimize toxicity and enhance palatability, thereby tailoring them to specific therapeutic requirements (Hou et al., 2023).

### 2.1 Origin and development of rhubarb processing

Rhubarb processing has been practiced for thousands of years. The earliest documentation, from the Han Dynasty, describes techniques such as “removing black skin” and “wine washing, wine soaking” found in Zhang Zhongjing’s *Golden Chamber Jade Letter Classic* (*Jingui Yuhan Jing*) (Zhang et al., 2021). Thereafter, a detailed account from the Southern and Northern Dynasty in *Master Lei*’*s Discourse on Drug Processing* (*Lei Gong Pao Zhi Lun*) noted that rhubarb was “carefully cut, steamed for 5 h, sun-dried, then sprinkled with melted wax and steamed for 9 h, repeated seven times and sun-dried again, and then sprinkled with diluted honey water and steamed for 24 h until the cross-section turned black, dried in the sun for optimal use”. This record also profoundly influenced subsequent generations.

In the early stages, people only divided rhubarb into two categories: raw and cooked. The ancient people had a broad understanding of “cooking” rhubarb. No matter what method was used to heat the rhubarb to achieve the goal of alleviating diarrhea and side effects, it can be included in the scope of “cooking”. Such as calcination, steaming, simmering, boiling, stir frying and so on (Wang Y. et al., 2018). During the Tang and Song dynasties, techniques such as “boiling with wine”, “steaming with wine” and “steaming under rice” were developed. The use of vinegar in rhubarb processing was first noted in the Tang Dynasty’s *Materia Medica for Dietotherapy* (*ShiLiao BenCao*). And the “nine times steaming and nine times drying in the sun” in *General Records of Holy Universal Relief* (*ShengJi ZongLu*) of the Song Dynasty can be said to be the earliest application of the method for processing “*JiuZhengJiuShai*” rhubarb.

In the Jin and Yuan dynasties, rhubarb processing methods became increasingly diverse. The *Miraculous Book of Ten Medicine* (*Shiyao Shenshu*) of the Yuan Dynasty described a method considered the official initiation of rhubarb charcoal production: “Burn until the surface is charred black on the outside, but the inner layer retains its original flavor, grind it into an extremely fine powder”. In Ming and Qing dynasties, the processing of rhubarb with wine as an auxiliary ingredient became very popular (Ren et al., 2023). The method of wine soaking and steaming over water that emerged in this period laid the foundation for the modern sealed wine stewing method of cooked rhubarb (Wang Y. et al., 2018), and also distinguished it from the wine stir-fried rhubarb. The detailed processing methods of rhubarb used in past dynasties are documented in Supplementary Table S1.

**FIGURE 1:**
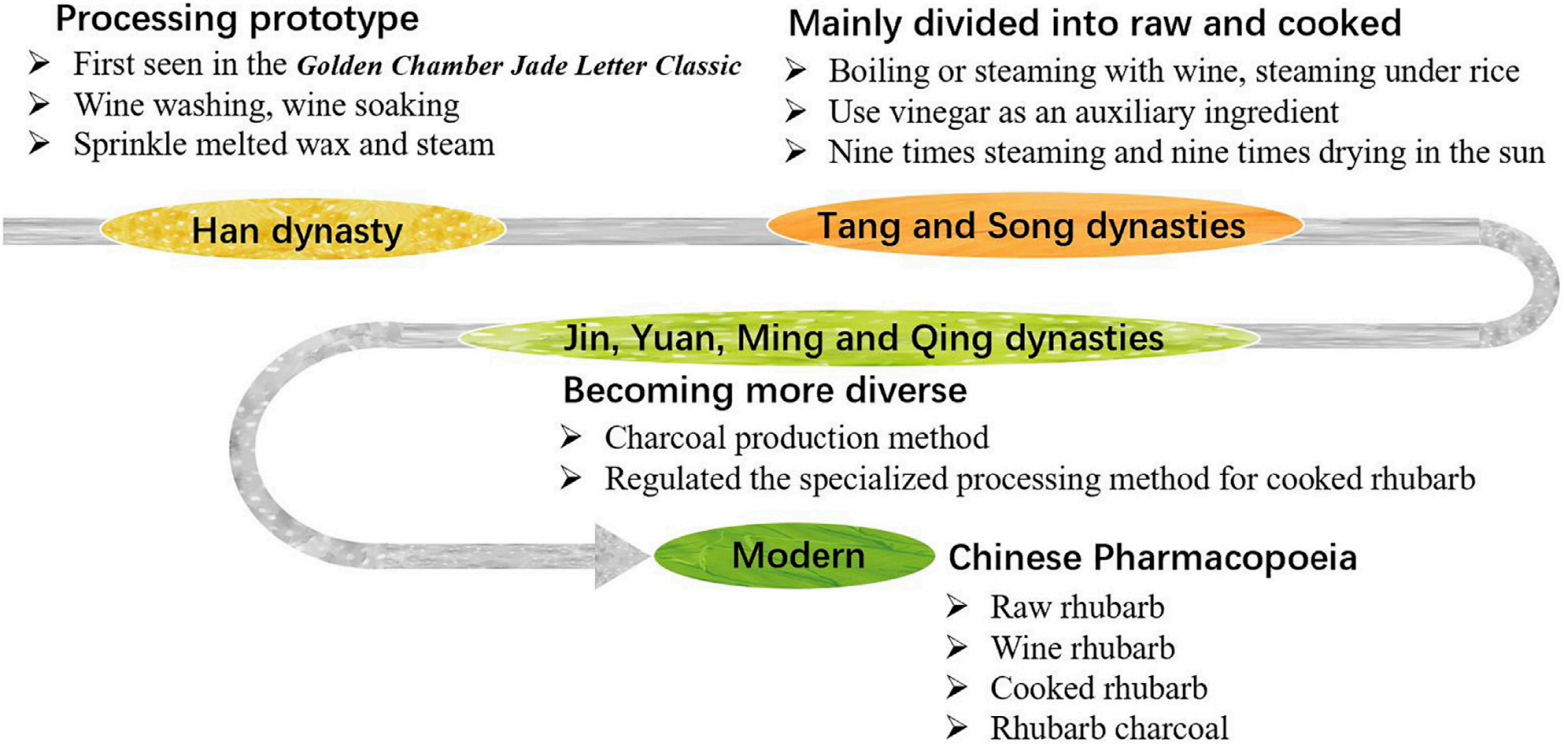
Research on the origin and development of rhubarb processing.

In modern times, the processing methods of rhubarb have become gradually refined. The state has organized various traditional rhubarb processing methods, established the “*General rules of Chinese medicine processing*”. In 1963, several monographs on traditional Chinese medicine processing, including *Integration of Chinese Medicine Processing Experience* were published. In the same year, for the first time, the government included raw rhubarb, wine rhubarb, cooked rhubarb and rhubarb charcoal, which renowned for their long history, extensive use and significant therapeutic effects, into the second edition of the *Pharmacopoeia of the People*’*s Republic of China*, and have been used as official norms since 1985. In addition, the 1988 *National Standards for Traditional Chinese Medicine Processing* and subsequent processing standards for local provinces have also played crucial roles in unifying and standardizing rhubarb processing by selecting and incorporating current, practical concoctions and their techniques from all over the country **(** Figure 1 **)**.

### 2.2 Compounds in different concoctions of rhubarb

**FIGURE 2:**
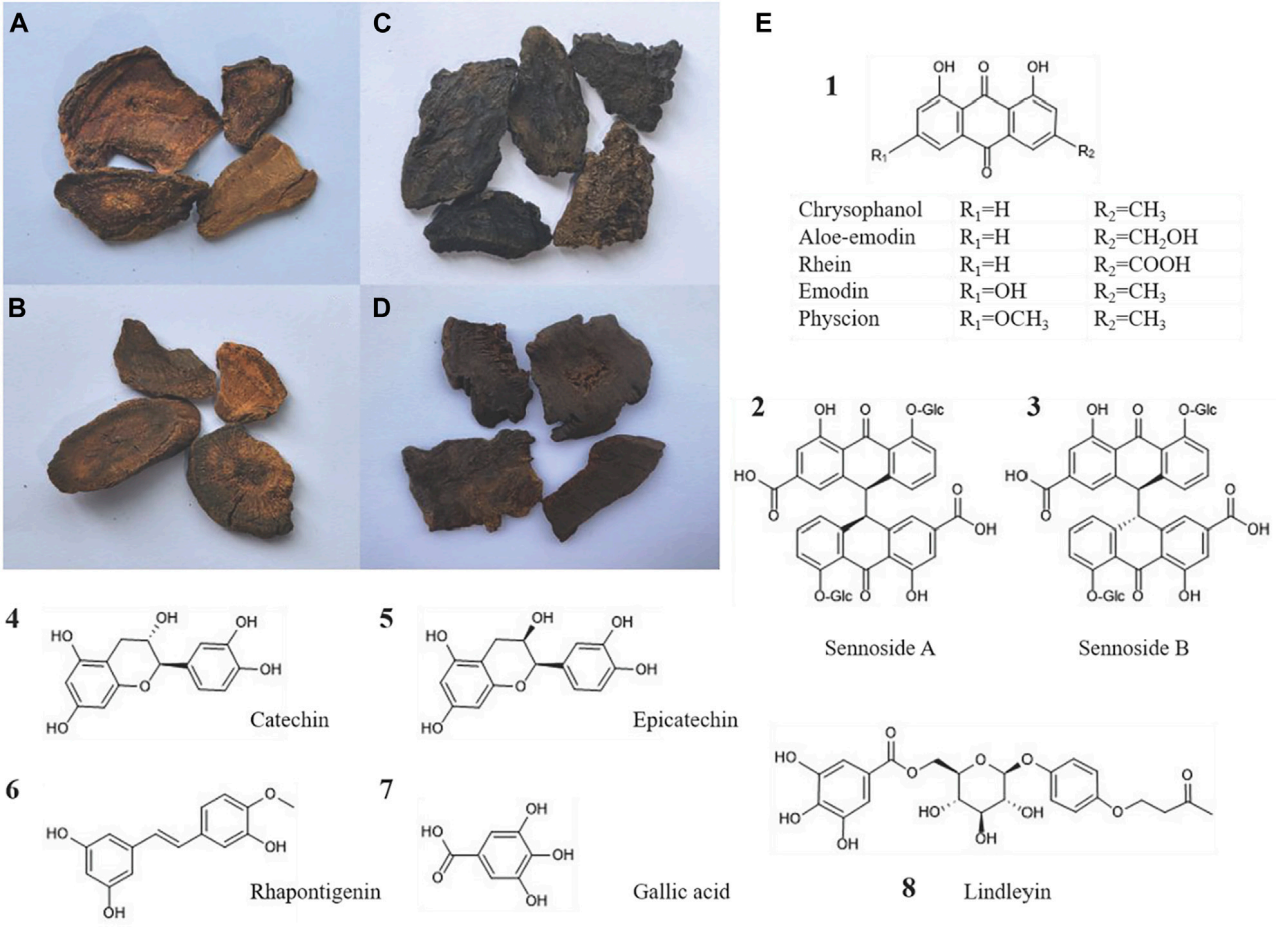
Four types of rhubarb concoctions and the chemical structural formulas of the main active compounds of rhubarb: **(A)** Raw rhubarb, **(B)** Wine rhubarb, **(C)** Cooked rhubarb, **(D)** Rhubarb charcoal. **(E)** The chemical structural formulas of the main free anthraquinones, sennoside A/B, catechins, epicatechin, rhapontigenin, gallic acid, and lindleyin in rhubarb are respectively presented.

Current literature reveals that rhubarb contains over 100 compounds, primarily including anthraquinones, tannins, stilbenes, phenylbutanones (Gao et al., 2017; Yao et al., 2021). Anthraquinones, encompassing both derivatives and anthrone derivatives, are identified as the key characteristics and pharmacologically active compounds of rhubarb, offering effects such as laxative, anti-inflammatory, antibacterial, antiviral, and anticancer (Malik and Müller, 2016). These derivatives are classified into two types: free and combined. Free anthraquinones consist of compounds such as rhein, emodin, aloe-emodin, chrysophanol and physcion. Combined anthraquinones, typically glycosides, result from the linkage of free anthraquinones with glycosyl groups, and anthrone derivatives include compounds such as sennoside A–D (Cao et al., 2017). Tannins, another significant compound, display antioxidant, anti-inflammatory, antibacterial, hemostatic and anti-diarrheal effects (Qin et al., 2011; Laddha and Kulkarni, 2019; Marcińczyk et al., 2021). They are divided into hydrolyzable tannins, condensed tannins with gallic acid and catechin as structural units, and complex tannins that combine these types. Moreover, stilbene compounds such as rhapontigenin, piceatannol, and their derivatives (Wang et al., 2019) have recently been recognized for their considerable biological potential, exhibiting anti-inflammatory (Dvorakova and Landa, 2017), anticancer (De Filippis et al., 2017), antibacterial (Singh et al., 2019) and anti-aging activities (Dutta et al., 2023). Phenylbutanone, including lindleyin and isolindleyin, is noted for its anti-inflammatory and analgesic properties (Zhang K. X. et al., 2022; Figure 2).

The processing methods for rhubarb, as recorded in the *Chinese Pharmacopoeia*, include cleaning rhubarb root, removing impurities, washing, moistening thoroughly, and slicing into thick pieces or blocks, referred to as raw rhubarb. Wine rhubarb is produced by stir-frying raw rhubarb with wine. Cooked rhubarb is obtained by stewing or steaming raw rhubarb with wine until it turns uniformly black. Rhubarb charcoal is produced by stir-frying raw rhubarb without any additives until the surface is charred black and the interior turns burnt brown. The prevalence of glycosides in rhubarb’s effective compounds, which are highly soluble in water and readily hydrolyzed under acidic conditions, explains the preference to minimize water use in processing the four officially recognized rhubarb concoctions used in clinical practice. This also underlines the need for the “less soaking, more moistening” approach during cleaning (Wen et al., 2020), avoidance of steaming or boiling with water, and exclusion of vinegar as an additive. The reactivity of tannins with iron and their ease of oxidation may account for the historical use of bamboo knives for cutting rhubarb in the Song Dynasty and the modern elimination of sun drying. The high solubility of most effective compounds in ethanol supports the traditional use of wine in processing wine rhubarb and cooked rhubarb. These varied processing methods not only emphasize different functional aspects of rhubarb but also alter its medicinal properties, creating new therapeutic effects and broadening its clinical utility.

It can be seen from the processing method of raw rhubarb that the harvested rhubarb raw materials are made into raw rhubarb decoction pieces, which mainly reflects the purifying of medicinal materials, easy to be weighed and used, increasing the contact area between medicinal materials and solvents, which is conducive to the purpose of boiling out the effective compounds when making decoctions. The compounds in it have not changed. The high content of combined anthraquinones and sennosides, known for their laxative effects impart raw rhubarb with its bitter flavor, cold and descending properties, and potent purgative and obstruction-clearing actions. Some anthraquinones are also integral to rhubarb’s ability to promote blood circulation and remove blood stasis (Seo et al., 2012; Tan et al., 2018; Li et al., 2019). Current pharmacological research underscores that the heat-clearing and detoxifying effects of Chinese medicinal herbs are largely linked to their anti-inflammatory and antibacterial activities, offering a scientific basis for the attributed properties of rhubarb (Wang et al., 2019; Zhang et al., 2020).

However, the other processing methods are further processed on the basis of raw rhubarb, including different physical or chemical transformations such as hydrolysis, carbonization, oxidation, decomposition and condensation (Ni et al., 2012), which will inevitably alter the content or ratios of compounds in the medicinal material. This suggests that changes in compounds during these processing methods may be crucial for modifying properties, enhancing efficacy and reducing toxicity. To investigate these changes ulteriorly, a comprehensive literature search was conducted focusing on the compounds of wine rhubarb, cooked rhubarb and rhubarb charcoal before and after processing, aimed to elucidate patterns of compound changes of three processing methods of rhubarb by sorting, analyzing and summarizing. Relevant publications published in English or Chinese from January 2010 to April 2024 were identified in databases such as PubMed, Web of Science, Embase and CNKI using search terms “rhubarb”, “processing” and “composition”. Two authors (Lu Shanshan and Fan Peixuan) identified studies from these databases independently, and disagreements were solved by consultation. If it cannot be resolved, it was resolved through consultation with the corresponding author professor Wei Shaobin. The search strategy is shown in Supplementary Table S2, with the last search date of 10 May 2024.

**FIGURE 3:**
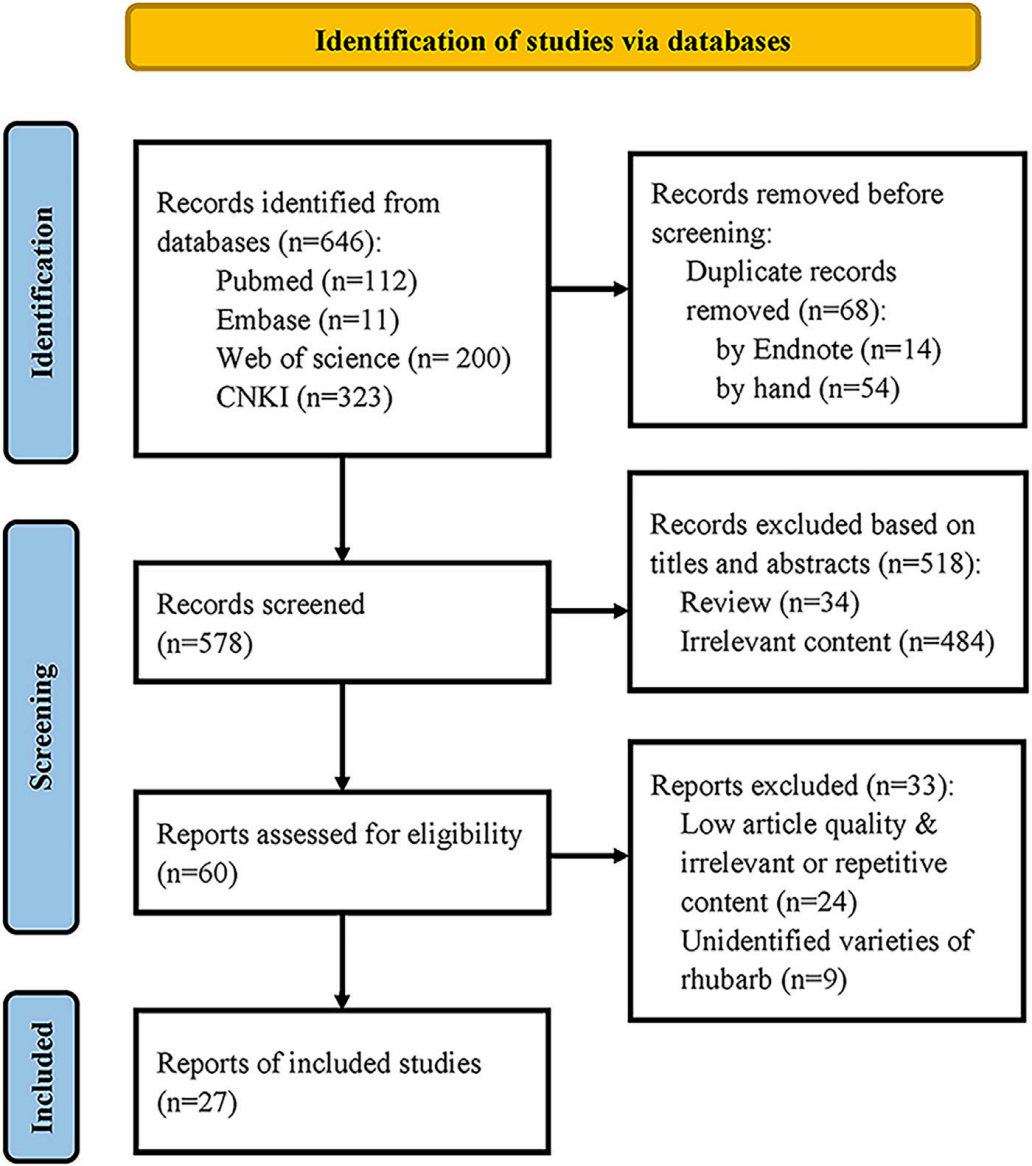
Flow chart of literature selection.

Preset inclusion criteria: (1) Study on the comparison of compounds before and after processing of wine rhubarb, cooked rhubarb and rhubarb charcoal (containing at least one of them); (2) Study published in English or Chinese; (3) The medicinal materials were identified as rhubarb species included in the *Chinese Pharmacopoeia*. Exclusion criteria: (1) Studies where full text is not available; (2) Repeated studies; (3) Non original studies. Finally, according to PRISMA statement of systematic review, there are 27 literature remained, as shown in Figure 3.

We extracted the following details from each study: (1) The first author’s name and publication year; (2) The rhubarb concoctions used and the specific processing methods; (3) The method used to determine the content of the compound and the solvent used to prepare the sample; (4) The difference in compound detection between raw rhubarb and other rhubarb concoctions. Two authors (Lu Shanshan and Fan Peixuan) extracted the data independently, and disagreements were solved by consultation. If it cannot be resolved, it was resolved through consultation with the corresponding author, professor Wei Shaobin. The characteristics of the included studies are shown in Supplementary Tables S3–S5.

We applied a self-designed scale to evaluate the methodological quality of the included study: (1) Optimized the content determination method; (2) Conducted precision, stability, and repeatability tests for detection; (3) Reported the specific values measured in the results section; (4) Reported statistical methods; (5) Potential conflict of interest Statement. Two authors (Lu Shanshan and Fan Peixuan) evaluated the quality of studies independently, and disagreements were solved by consultation. If it cannot be resolved, it was resolved through consultation with the corresponding author professor Wei Shaobin. The results showed that out of the 27 articles, 18 optimized the conditions for compound content determination, such as the mobile phase used in chromatography and the method of sample preparation; 20 conducted the precision, stability and repeatability tests; 17 articles reported numerical information on the measured compounds in the results section, while the remaining 10 articles indirectly described changes in the compounds through graphics or arrows; 16 studies did not clearly report the statistical methods used; Only two articles have made potential conflict of interest statements. Among them, there are 14 studies that meet the above three conditions, 10 studies that meet the two conditions, and three studies that meet the one condition. The research quality list is shown in Supplementary Table S6.

#### 2.2.1 Wine rhubarb

According to our statistics, 13 articles have described the differences in the compounds between wine rhubarb and raw rhubarb. The main compounds that increased in rhubarb after being fried with wine include free anthraquinones, tannins (gallic acid, catechin, gallic acid-3-O-glucoside), phenylbutanones [4-(4′hydroxyphenyl)-2-butanone, 4′-hydroxy-phenyl-2-butanone-4′-O-β-D-(6″-galloyl)-glucoside, 4′-hydroxyphenyl-2-butane-4′-O-β-D-(6″-O-cinnamoyl)-glucoside]. Main compounds that decreased include combined anthraquinones, anthrones, tannins [(epi)catechin-O-gallate, cinnamoyl-O-glucose-O-galloyl, cinnamoyl-O -glucose-O-digalloyl], stilbenes [trans-3,5,4′- trihydroxyvinyl-4′-O-β-D-glucoside, trans-3,5,4′- trihydroxystilbene-4′-O-β-D-(6″-O-galloyl)-glucoside], phenylbutanones [4′-hydroxyphenyl-2-butanone, 4-hydroxyphenyl-2-butane-4′-O-β-D-(2″-O-galloyl-6″-O-(4″-hydroxy)-cinnamoyl)-glucoside].

#### 2.2.2 Cooked rhubarb

There are 21 articles described the differences in the compounds between cooked rhubarb and raw rhubarb. The main compounds of rhubarb increased after steaming with wine include free anthraquinones, tannins (gallic acid, catechin, gallic acid-3-O-glucoside), 5-hydroxymethyl furfural (5-HMF), 4-(4’hydroxy-phenyl)-2-butanone. Main compounds that decreased include combined anthraquinones, anthrones, macromolecular tannins, stilbenes [trans-3,5,4′-trihydroxystilbene-4′-O-β-D-glucoside, trans-3,5,4′-trihydroxystilbene-4′-O-β-D-(6″-O-galloyl)-glucoside], phenylbutanones [4′-hydroxyphenyl-2-butanone, 4′-hydroxyphenyl-2-butanone-4′-O-β-D-(6″-galloyl)-glucoside, 4′-hydroxyphenyl-2-butane-4′-O-β-D-(6″-O-cinnamoyl)-glucoside, 4′-hydroxyphenyl-2-butane-4′-O-β-D-(2″-O-Galloyl-6″-O-(4′-hydroxy)-cinnamoyl)-glucoside].

#### 2.2.3 Rhubarb charcoal

The compounds differences between rhubarb charcoal and raw rhubarb were compared in 18 articles. The main compounds that increased after frying rhubarb into charcoal include free anthraquinones, gallic acid-3-O-glucoside, 5-HMF, phenylbutanones [4′-hydroxyphenyl-2-butanone, 4-(4′hydroxyphenyl)-2-butanone)]. Main compounds that decreased include combined anthraquinones, anthrones, macromolecular tannins, stilbenes [trans-3,5,4′-trihydroxystilbene-4′-O-β-D-glucoside, trans-3,5,4′-trihydroxystilbene-4′-O-β-D-(6″-O-galloyl)-glucoside], phenylbutanones [4′-hydroxyphenyl-2-butanone, 4′-hydroxyphenyl-2-butanone-4′-O-β-D-(6″-galloyl)-glucoside, 4′-hydroxyphenyl-2-butane-4′-O-β-D-(6″-O-cinnamoyl)-glucoside, 4′-hydroxyphenyl-2-butane-4′-O-β-D-(2″-O-Galloyl-6″-O-(4′-hydroxy)-cinnamoyl)-glucoside].

#### 2.2.4 The influences of stir-frying with wine, steaming with wine and stir-frying into charcoal on the compounds of rhubarb

Wine rhubarb and cooked rhubarb both incorporate yellow wine as an auxiliary ingredient. However, wine rhubarb undergoes a brief stir-frying process over slow heat after being moistened with wine. Conversely, cooked rhubarb is stewed or steamed in a sealed container with a greater quantity of yellow wine until it turns black, undergoing a more intense processing. Rhubarb charcoal is stir-fried until carbonized, involving the highest processing temperatures and longest duration, which is the most intensive. Multiple literature has demonstrated through principal component analysis that there are significant differences in the compounds of rhubarb before and after processing (Wang et al., 2014; Zhao et al., 2014; Sun et al., 2024). According to our data, processing increases the levels of free anthraquinones, tannin monomers and other small molecule compounds like 5-HMF in rhubarb. Conversely, the contents of combined anthraquinones, sennosides, tannins, stilbenes and phenylbutanones, which are larger molecules, decrease significantly, especially in cooked rhubarb and rhubarb charcoal. This reduction may result from the decomposition of macromolecular compounds into monomers at varying degrees under high-temperature conditions. Moreover, the processing of rhubarb involves not only the hydrolysis of glycosides but also complex chemical changes, including interactions among different types of compounds, which can alter the herb’s efficacy and properties (Yan et al., 2016).

Studies indicate that the extent of processing affects the levels of certain compounds. For example, the compounds in raw rhubarb and wine rhubarb remains relatively similar, while significant changes occur in cooked rhubarb and rhubarb charcoal (Li et al., 2010; Tian et al., 2010). For instance, the degradation of anthraquinones in wine rhubarb is minimal, with little total change, primarily shifting between combined and free forms. However, due to prolonged heating, the total anthraquinone content in cooked rhubarb is somewhat reduced, and the compounds in rhubarb charcoal are severely degraded (Li H. F. et al., 2011; Li, 2011). As previously noted, compounds like sennoside and anthraquinone glycoside are crucial for the laxative effects of rhubarb. Most free anthraquinones are absorbed before reaching the colon, while the combined anthraquinones are significant as they metabolize into free forms in the intestine (Wang et al., 2015a). This explains why the bitter flavor, cold and descending properties, and strong purgative and blockage-removing effects of processed rhubarb are mitigated, with cooked rhubarb having a weaker effect compared to wine rhubarb, and rhubarb charcoal having the least impact. The intestinal absorption characteristics of anthraquinones in rhubarb are very important for oral traditional Chinese medicine to achieve the expected therapeutic effect. However, there is still a lack of deeper understanding of their absorption characteristics (Ta et al., 2023).

TCM posits that yellow wine, known for its fragrant aroma and rising, diverging properties, can warm and unblock blood vessels, thereby promoting blood circulation and removing blood stasis. Consequently, wine rhubarb and cooked rhubarb, which are processed with wine, are believed to enhance these effects (Zhu et al., 2010; Yang et al., 2012). Wine rhubarb significantly alters the distribution of free anthraquinones in rats, increasing their presence in heart and lung tissues (Wu et al., 2017). This provides experimental support for its use in clearing heat toxicity from the blood phase in the upper energizer. Additionally, *in vivo* experiments demonstrated that compounds such as rhein, emodin, aloe-emodin, physcion, chrysophanol and gallic acid exhibit improved absorption in pathological state of blood stasis in rats and rabbits (Dai et al., 2014; Zhu et al., 2017). Gallic acid also reduces whole blood viscosity, plasma viscosity and plasma fibrinogen levels in blood stasis model rats (Sun et al., 2022), underscoring cooked rhubarb’s potent effect in promoting blood circulation and removing blood stasis due to its significantly increased content of free anthraquinone and gallic acid (Li, 2011; Yan et al., 2016). Furthermore, the carbonization at high temperatures creates many loose pore structures, beneficial for rhubarb charcoal in adsorbing and astringing (Chen et al., 2019), thereby enhancing its hemostatic effect. This aligns with other findings, who noted that rhubarb charcoal exhibits the strongest hemostatic effect among the four concoctions (Zhu et al., 2008).

In a minority of studies, some inconsistencies with the above trends were observed. For instance, the content of emodin-8-O-β-D-glucoside and two phenylbutanone glycosides in rhubarb which have larger molecular weight increased after being stir-fried with wine, likely due to the ethanol in wine may make these compounds more soluble under less intense processing conditions (Li et al., 2010; Tian et al., 2010). Several studies also reported a decrease in the content of some free anthraquinones and tannin monomers in rhubarb after processing (Wang et al., 2015b; Yan et al., 2016; Zhang Q. et al., 2022). During the process of frying rhubarb into charcoal, the content of gallic acid, 5-HMF and free anthraquinones showed a pattern of first increasing and then decreasing (Yang et al., 2020). This may be related to the sublimation of free anthraquinones (Wu et al., 2020) and the intense processing, which led to the destruction of these compounds. Given the characteristics and complexity of plant materials, any analysis of plant compounds requires the application of appropriate plant sample preparation procedures in order to extract the compounds to be analyzed from the matrix. The extraction rates of each compound vary in different solvents (Nan et al., 2019). It has also been reported that the concentration of water in the extractant can affect the accuracy of the determination of anthraquinone glycoside content (Wianowska, 2014). Therefore, different sample preparation procedures, solvents and the specific processing temperature and time used by different scholars may also be the reasons for the differences in conclusions.

The heterogeneity among included studies may affect the summary of the compound changes before and after processing of rhubarb, and we can draw some insights for further research. Optimizing the detection conditions and sample preparation methods before starting the experiment can help obtain more efficient, accurate and sensitive results. Secondly, the examination and reporting of linearity, precision, stability, repeatability and recovery rate of the methodology can reduce the risk of research result bias. The raw data and statistical methods used in studies are also important features for evaluating the quality of evidence. Optimizing the quality of experimental design and reports may be an issue that needs attention in the future.

## 3 Modern pharmacological effects of rhubarb

Rhubarb is extensively utilized in TCM due to a variety of effects, increasingly supported by research into its monomer compound. These effects are primarily attributed to various compounds found in rhubarb, such as anthraquinone derivatives, organic acids, volatile oils, glycosides and tannins. Recent studies have also highlighted its potential in anti-tumor applications (Supplementary Table S7).

### 3.1 Laxative

**FIGURE 4:**
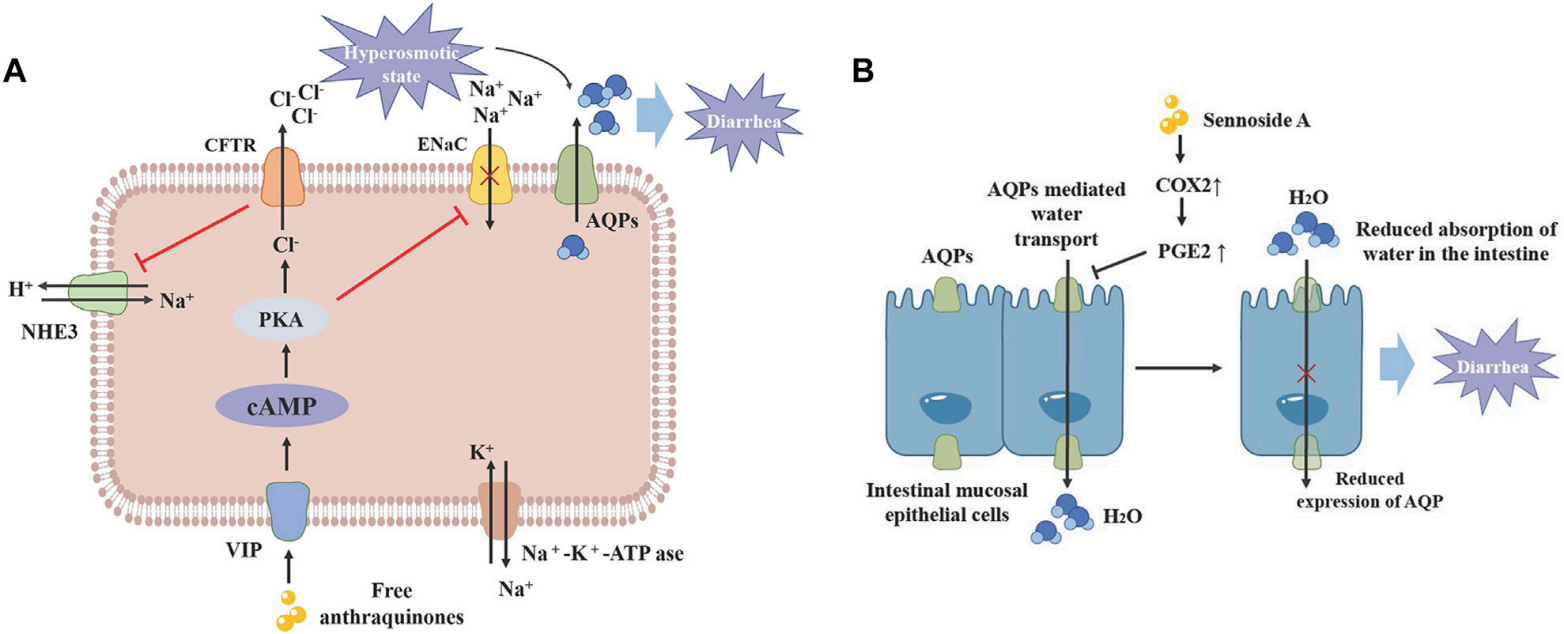
The laxative mechanism of monomer in rhubarb. **(A)** Free anthraquinone can upregulate VIP expression, promote CAMP/PKA signaling pathway transduction and CFTR expression, while inhibiting ENaC and NHE3 expression, increasing intestinal osmotic pressure and leading to diarrhea. **(B)** Sennoside A can inhibit the expression of COX2 and the release of PEG2 in macrophages, thereby reducing the expression of AQPs in the colon and inhibiting the absorption of water in the intestine, leading to diarrhea. CFTR: Cystic fibrosis transmembrane conductance regulator. AQP: Aquaporin. VIP: Vasoactive intestinal peptide. NHE3: Na*/H exchanger 3. ENaC: Epithelial sodium channel.

Modern pharmacological studies have verified that sennoside A, an anthraquinone compound in rhubarb, its laxative impact may be due to its inhibition of water transfer through decreased expression of aquaporin-3 (AQP3) in the colon. This effect is mediated by anthrone, an active metabolite of sennoside A, which activates macrophages in the colon and accelerates prostaglandin E2 (PGE2) secretion (Kon et al., 2014). Additionally, free anthraquinones facilitate stool softening and promote laxation by increasing serum levels of vasoactive intestinal peptide (VIP), motilin (MTL) and substance P (SP), up-regulating the expression of VIP, cyclase-associated protein 1 (CAP1) and protein kinase A (PKA), and the expression of epithelial sodium channels (ENaC), and Na^+^/H^+^ exchanger 3 (NHE3) (Lv et al., 2024). Moreover, the laxative effects of rhubarb are also linked to its regulatory impact on intestinal flora and metabolism (Yang et al., 2022; Figure 4).

### 3.2 Anti-bacterial

**FIGURE 5:**
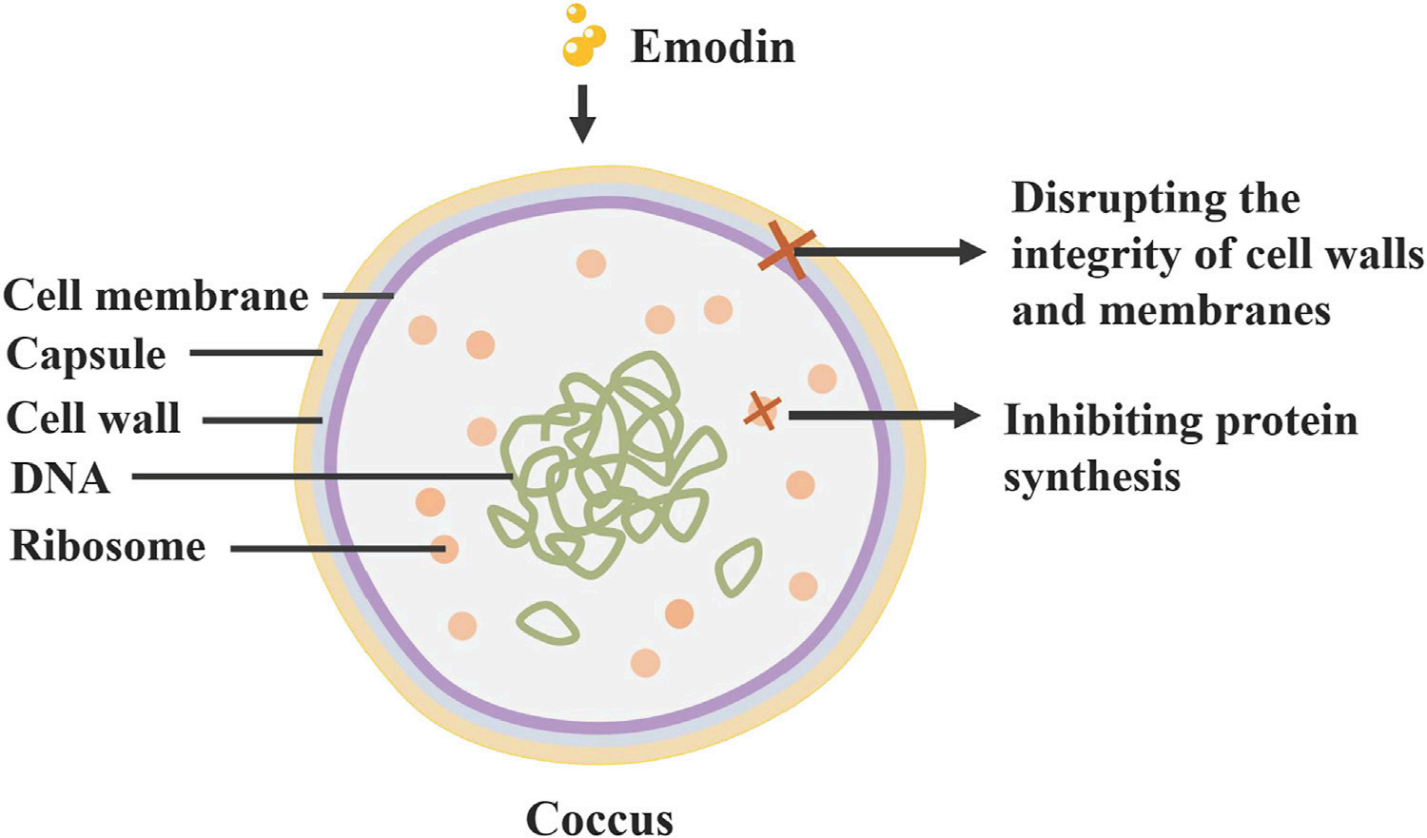
Antibacterial effect of rhubarb: Anthraquinone compounds in rhubarb can inhibit the growth of *Staphylococcus aureus* by disrupting its cell wall and membrane, and inhibiting its protein synthesis.

Research on the bacteriostatic properties of rhubarb’s monomers shows that free anthraquinones (including emodin, rhein, aloe-emodin) possess significant bacteriostatic activity against a broad spectrum of bacteria, such as *Staphylococcus aureus*, *Lactobacillus* and *Escherichia coli* (Stompor-Gorący, 2021). Rhubarb inhibits the growth of *S. aureus* by compromising the integrity of its cell wall and cell membrane (Xu et al., 2021). Additionally, aqueous extracts of rhubarb can prevent biofilm formation by down-regulating transduction systems and altering the levels of DNA-binding proteins and transcriptional regulators (Ding et al., 2017). *Chlamydia trachomatis* is a specialised pathogen that has become an exclusive intracellular pathogen due to a drastically reduced coding capacity, resulting in its dependence on the host cell for nutrient supply (Stelzner et al., 2023). While it does not directly inactivate *C. trachomatis*, it inhibits infection by modulating pathogen-host cell interactions (Yu et al., 2022; Figure 5).

### 3.3 Anti-inflammatory

**FIGURE 6:**
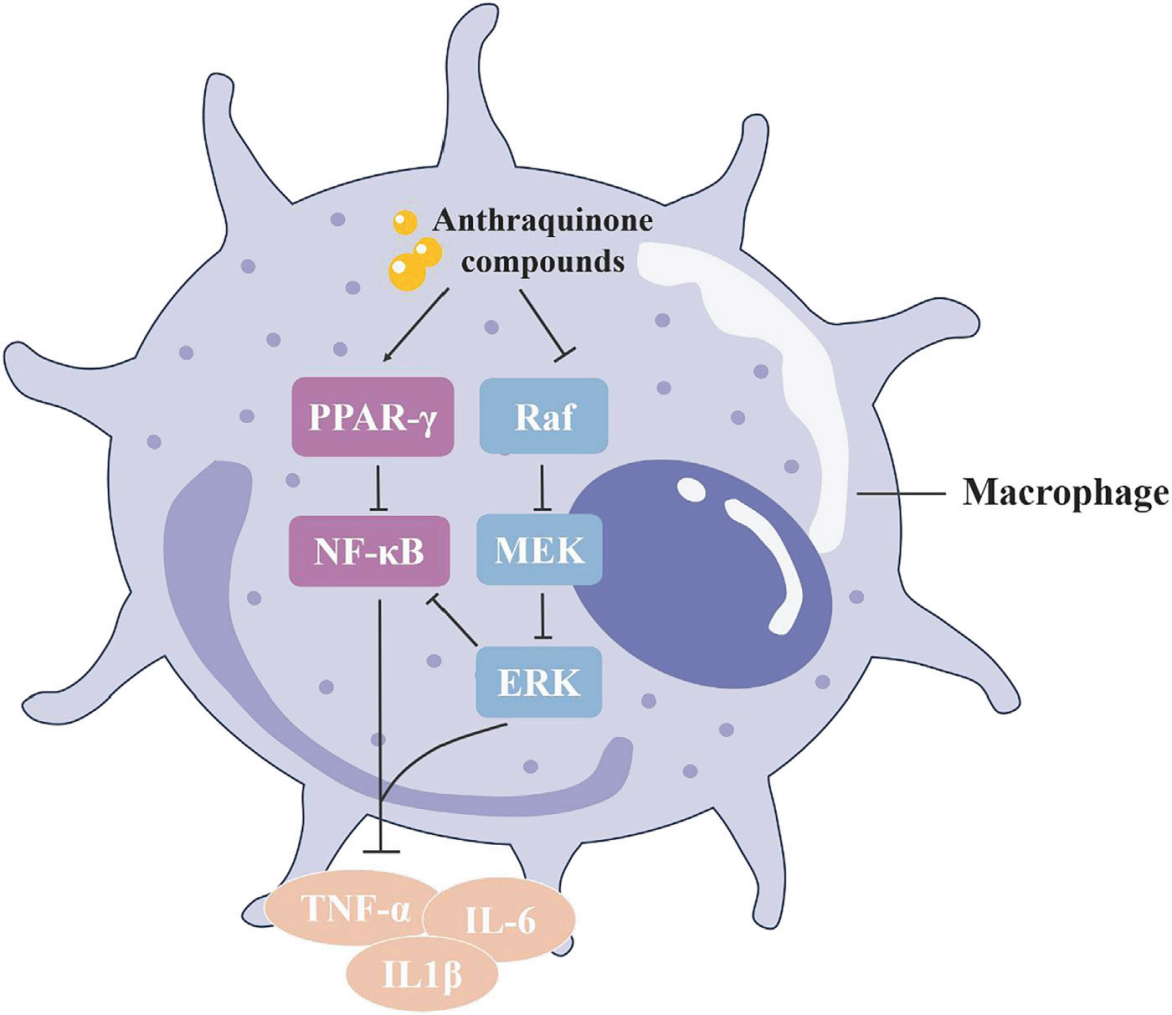
The ant-inflammatory mechanism of anthraquinone in rhubarb: The anthraquinone compounds in rhubarb can promote PPAR–y signaling in macrophages and inhibit phosphorylation of the MAPK signaling pathway, thereby inhibiting downstream NF–к B activation and the release of inflammatory factors, reducing inflammation.

Numerous active compounds in rhubarb display potent anti-inflammatory activity, with anthraquinones like emodin, rhein, chrysophanol and aloe-emodin being particularly significant. Studies involving lipopolysaccharide (LPS)-induced RAW264.7 cells have shown that rhein inhibits the production of pro-inflammatory cytokines (interleukin-6 [IL-6], IL-1β and tumor necrosis factor-α [TNF-α]), and reduces NF-κB p65 levels. In LPS + ATP-induced RAW264.7 macrophages, rhein also diminished the expression of NALP3 inflammasome and cleaved IL-1β, suggesting that rhein’s anti-inflammatory effects may stem from its ability to inhibit both NF-κB and NALP3 inflammasome pathways (Ge et al., 2017). Emodin and chrysophanol are noted to have similar effects (Zhu et al., 2016b; Wen et al., 2018). Notably, the anti-inflammatory effectiveness of aloe-emodin exceeds that of emodin and rhein at the same dosage, a difference attributed to its molecular structure (Hu, 2019; Figure 6).

### 3.4 Regulation of blood coagulation

**FIGURE 7:**
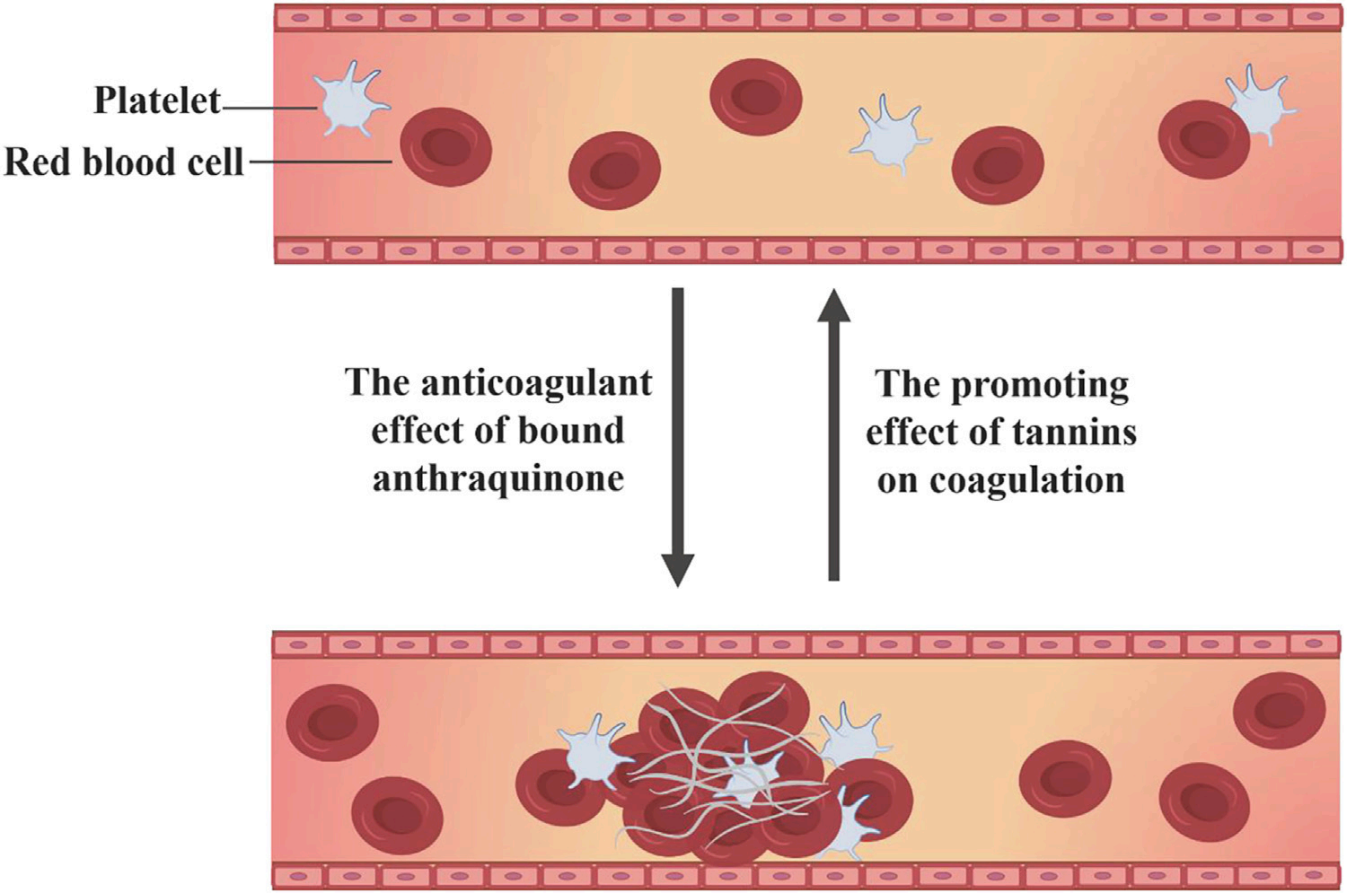
Bidirectional regulation of coagulation function by rhubarb compounds.

Rhubarb demonstrates a bidirectional regulatory effect on blood coagulation, capable of both activating and stopping bleeding through its diverse active compounds. Research based on network pharmacology has identified that key compounds in rhubarb, emodin-8-O-β-D-glucopyranoside, physcion-8-O-β-D-glucopyranoside and 3′-O-gallate, significantly enhance blood rheological parameters and arterial blood flow in hyperviscosity syndrome (HVS) rats, with anticoagulant effects comparable to aspirin (Gao et al., 2020). Conversely, the same compounds in rhubarb also perform hemostatic functions. Aqueous extracts of *R. palmatum L*. have shown pronounced vasoconstrictive effects on blood vessels, with d-catechin and gallic acid identified as the hemostatic active compounds through compound isolation, pharmacological screening and chemical structure identification (Du et al., 1983). Studies on *R. tanguticum Maxim.ex Balf.* from different production areas have also confirmed significant hemostatic and platelet-increasing effects in *R. tanguticum Maxim.ex Balf.* from the four main production areas of Qinghai (Wang S. W. et al., 2015). A systematic strategy based on computational simulation, biological validation and biophysical research was used in the experiment, which confirmed that gallic acid can directly inhibit thrombin and its induced platelet aggregation, and can also bind to thrombin protein and rapidly stabilize protein conformation (Zhang Y. et al., 2022). However, the current research on hemostasis of gallic acid mostly focuses on the hemostatic materials with gallic acid esterification and gallic acid compounds as raw materials (Trombetta et al., 2024; Yu et al., 2024). There is a lack of reliable experimental research on the mechanism through which d-catechin and gallic acid in rhubarb exert hemostatic effects (Figure 7).

### 3.5 Digestive system protective effects

**FIGURE 8:**
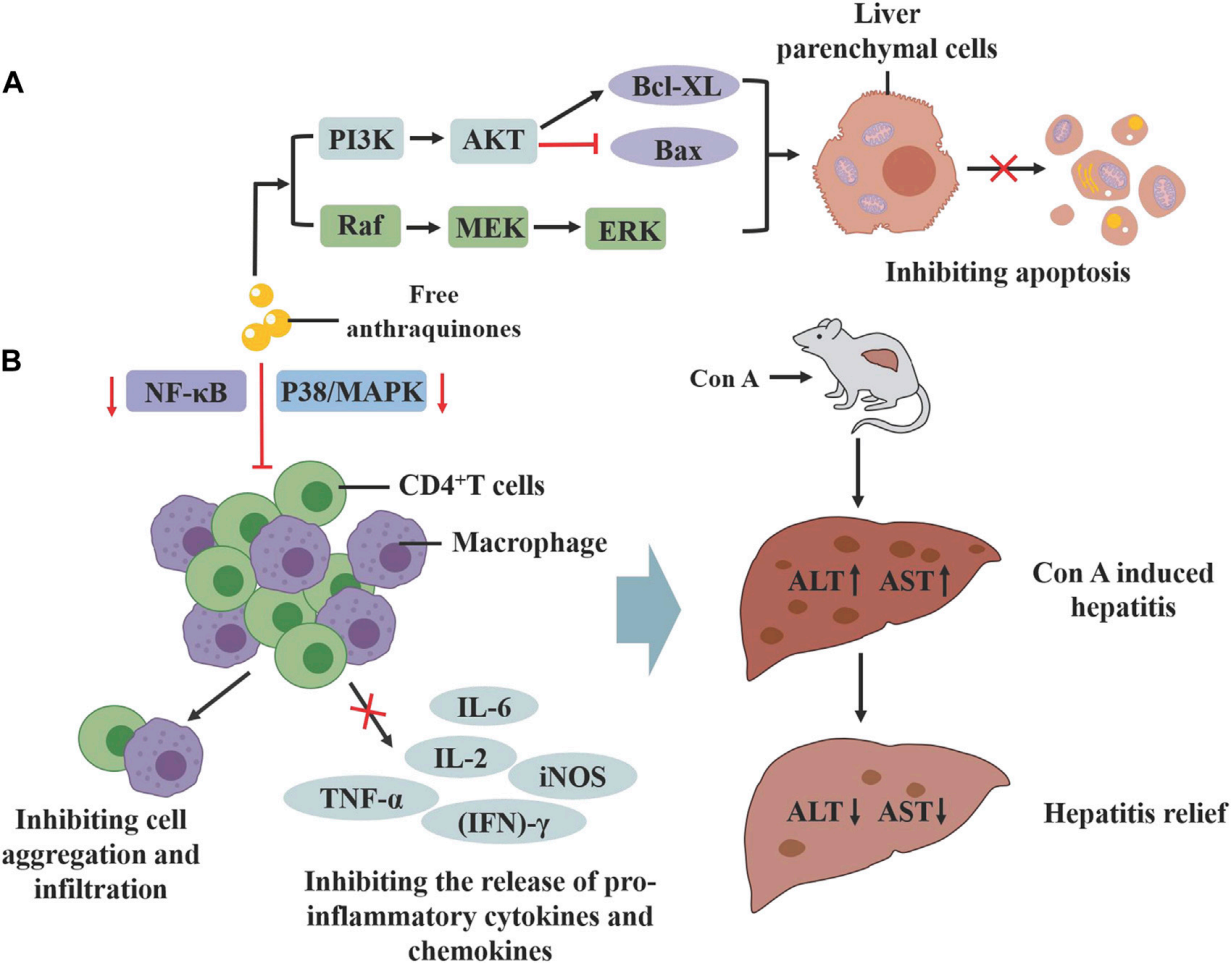
The protective effect of active compounds in rhubarb on the digestive system. **(A)** Rhein can activate the PI3K/AKT and MAPK signaling pathways, inhibiting liver cell apoptosis. **(B)** Emodin can inhibit the p38-MAPK and NF-KB signaling pathways in CD4+cells and macrophages, alleviating ConA induced liver injury.

Rhubarb exerts a protective effect on various organs of the digestive system. In studies examining the effects of the classic formula *Yinchenhao Decoction* (YCHD)*,* with rhubarb as the main medication, on hepatic fibrosis, treatment with rhein in human hepatic L02 cells notably decreased the protein level of cleaved cysteine-3, and increased the expression of p-ERK1/2, PI3K and Bcl-XL proteins. *In vivo* experiments further demonstrated that treatment with YCHD not only alleviated liver fibrosis symptoms but also reduced apoptosis in hepatic parenchymal cells, confirming the decoction’s ability to inhibit hepatic fibrosis in rats by regulating apoptosis (Cai et al., 2019). Moreover, emodin mitigates concurrent Concanavalin A (Con A)-induced liver injury in mice by inhibiting infiltration and activation of CD4^+^ and F4/80+ cells, and suppressing the p38- MAPK-NF-κB pathway in CD4^+^ T cells and macrophages (Xue et al., 2015). In addition, emodin may alleviate pancreatic injury by correcting the Treg/Th cell imbalance and inhibiting the inflammatory response, thereby mitigating severe acute pancreatitis (Wu et al., 2019; Figure 8).

### 3.6 Anti-tumor

**FIGURE 9:**
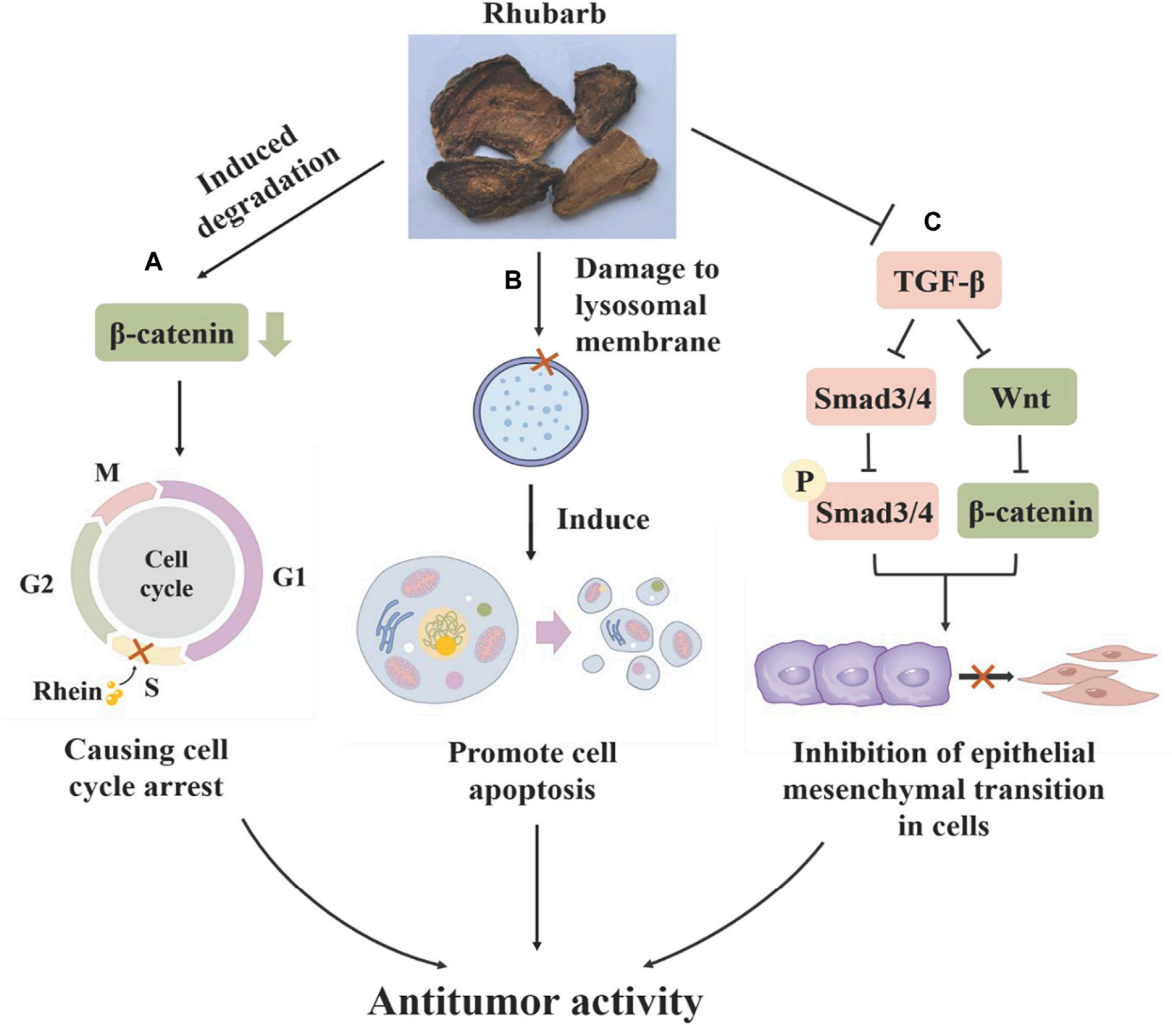
The mechanism of rhubarb in inhibiting gynecological malignant tumors: **(A)** Rhein can induce the degradation of ẞ-Catenin protein, causing cell cycle arrest in the S phase and inhibiting the proliferation of cervical cancer HeLa cells. **(B)** Rhubarb can damage the lysosomal membrane, increase the activity of tissue protease, and induce cell apoptosis. **(C)** Rhubarb can inhibit TGF-B/Smads and Wnt/B-catenin signaling pathways, thereby inhibiting the epithelial mesenchymal transition of tumor cells.

Rhubarb exhibits a potent inhibitory effect on various tumors across the respiratory, digestive and reproductive systems, including lung adenocarcinoma (Shia et al., 2011), gastric cancer (Chen et al., 2007), pancreatic cancer (Pan et al., 2016), cervical cancer (Liu et al., 2018) and ovarian cancer (Zhou et al., 2017). It effectively inhibits tumor cell growth (Liu et al., 2018), suppresses tumor invasion and migration (Chen et al., 2010; Zhou et al., 2017), and prevents the formation of tumor neovascularization, playing a crucial role in various stages of tumor progression by targeting different mechanisms. The anti-tumor effects of rhubarb are primarily attributed to a range of compounds extracted from it, such as emodin, rhein, aloe-emodin and stilbene (Cao et al., 2017). Rhein can regulate cyclin D1 by inducing the degradation of β-catenin, arresting the cell cycle in S-phase and inhibiting the proliferation of cervical cancer HeLa cells (Liu et al., 2018). It has been shown to induce apoptosis in human nasopharyngeal carcinoma cells through endoplasmic reticulum stress and a Ca2^+^-dependent mitochondrial death pathway and to inhibit the migration and invasion of human tongue cancer SCC-4 cells and human ovarian cancer SKOV3-PM4 cells by regulating matrix metalloproteinases (MMPS) (Chen et al., 2010; Zhou et al., 2017). The Wnt/β-catenin signaling pathway, crucial in cell proliferation and invasion in lung cancer, gastric cancer, ovarian cancer and nephroblastoma, is negatively regulated by rhubarb, which promotes β-catenin protein degradation and inhibits tumor metastasis (Tsai et al., 2013). In advanced stages of cancer, TGF-β signaling has been shown to promote invasiveness and metastasis by inducing the expression of Snail and other transcription factors, leading to epithelial mesenchymal transition (EMT) (Peinado et al., 2003). Emodin can inhibit the expression of β-catenin in SiHa and HeLa cervical cancer cells, downregulate transforming growth factor-β (TGF-β), and inhibit the Wnt/β-catenin signaling pathway, and thus suppress the occurrence of EMT (Thacker and Karunagaran, 2015). These findings indicate that rhubarb’s compounds can impact cancer-related biological processes through multiple signaling pathways, offering advantages over traditional cytotoxic drugs by reducing tumor drug resistance due to their multi-targeting properties. It has been demonstrated that emodin at a concentration of 10 µM can enhance the sensitivity of tumor cells to chemotherapy and radiotherapy by decreasing P-glycoprotein (P-gp) function and activating the mitochondrial apoptotic pathway *in vitro* (Liu et al., 2012; Li et al., 2016; Figure 9).

## 4 Influence of rhubarb concoctions on traditional pharmacological effects and clinical applications

**FIGURE 10:**
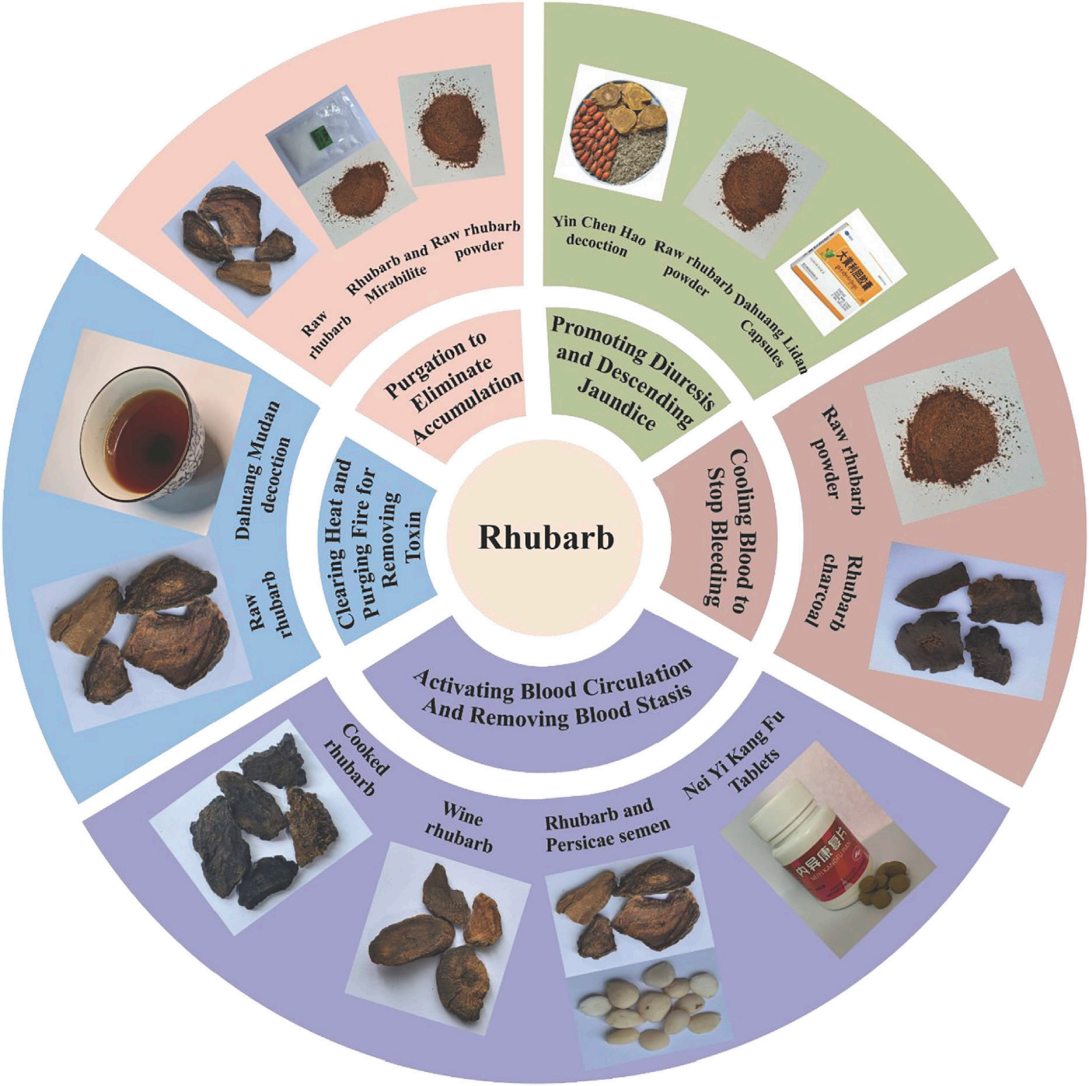
Influence of rhubarb concoctions on traditional pharmacological effects and clinical applications.

Different concoctions of rhubarb significantly impact its traditional pharmacological actions, primarily due to changes in compounds during processing. These alterations in traditional pharmacology across various concoctions are detailed in Supplementary Table S8. Throughout years of clinical practice, medical practitioners have recognized the limitations of using single herbs due to the complexity of diseases and the diversity of patient constitutions. Consequently, they have developed a variety of Chinese medicinal herb combinations, such as pairs of herbs, angular herbs and multi-herb formulas, tailored according to the principles of Chinese medicine’s diagnostic methods and the characteristics of the medicines (Wang et al., 2012). This extensive use of concoctions enhances the unique advantages and appeal of Chinese medicine in treating various diseases, as exemplified in the traditional use of rhubarb. We have compiled the main formulas of Chinese medicine that utilize different concoctions of rhubarb (Supplementary Table S9). Given the distinct main effects of each concoction, individual concoctions of rhubarb and their compound formulas are also widely employed in treating conditions like constipation, intestinal obstruction, hepatitis, gastrointestinal bleeding, malignant tumors and other diseases (Supplementary Table S10; Figure 10).

### 4.1 Purgation to eliminate accumulation

“Purgation to eliminate accumulation” refers to the process by which medications clear stagnation in the stomach and intestines and alleviate constipation, thereby treating related gastrointestinal issues. Raw rhubarb is a primary agent for this effect. The laxative efficacy of raw rhubarb tends to diminish when processed, likely due to the decomposition and inactivation of anthraquinones during processing. Studies comparing the laxative effects of four rhubarb concoctions (raw rhubarb, wine rhubarb, cooked rhubarb and rhubarb charcoal) on healthy mice found that only raw rhubarb and wine rhubarb exhibited significant laxative effects, occurring approximately 3 h post-administration. Wine rhubarb slightly delayed the onset and reduced the frequency and volume of diarrheal stools compared to raw rhubarb, which showed considerably stronger laxative properties. Further experiments on intestinal motility revealed that raw rhubarb produced the most potent diarrheal effects, wine rhubarb was moderately effective, and both rhubarb charcoal and cooked rhubarb exhibited some antidiarrheal properties (Yan et al., 2010). In an experiment analyzing the effect of 75% ethanol extract of different concoctions on the propulsion rate of small intestinal charcoal and the activity of Na^+^-K^+^-ATPase in colon intestinal wall cells, results indicated significant inhibition of Na^+^-K^+^-ATPase activity in all concoctions compared to controls, with raw rhubarb showing the strongest effect. Both raw and wine rhubarb increased the propulsion rate of small intestinal charcoal in rats, while cooked rhubarb and rhubarb charcoal decreased it, aligning with previous research (Li Y. et al., 2011). The diarrhea-causing potency of different rhubarb concoctions, assessed *via* the “parallel line method of quantitative response”, showed that raw rhubarb had the highest potency at 825.22 U/g, followed by wine rhubarb at 699.05 U/g, with cooked rhubarb weaker at 459.76 U/g, and rhubarb charcoal undetectable due to its low diarrheal potency (Li et al., 2012c). The comparative analysis of the laxative effects of raw and cooked rhubarb in a mouse model of heat-induced constipation revealed that the more potent effect of raw rhubarb might be induced by the stimulation of intestinal smooth muscle contractions, mediated by increased secretion of intestinal acetylcholinesterase (Ach E) and SP and the augmentation of intracellular Ca^2+^ concentration (Wu et al., 2014). Conversely, the diminished efficacy of cooked rhubarb may illustrate one mechanism behind the adage “raw rhubarb causes diarrhea, while cooked acts gradually”.

Raw rhubarb and its formulations are utilized in treating constipation, gastrointestinal dysfunction and intestinal obstruction. Supplementation with raw rhubarb extract has improved stool frequency and consistency in a dose-dependent manner in middle-aged constipated patients, without impacting safety indicators, thus confirming its reliability and safety for non-severe constipation cases. This therapeutic effect is likely linked to the modulation of the intestinal microbiota by raw rhubarb (Neyrinck et al., 2022). Additionally, significant enhancements in gastrointestinal dynamics have been observed in patients with severe acute pancreatitis treated with *Da Chengqi Decoction* combined with western medicine therapy (Xiu et al., 2023). Topical application of raw rhubarb powder to the *Shenque* acupoint has effectively improved functional constipation in Parkinson’s patients in terms of defecation frequency and stool character (Fan et al., 2015). External application of raw rhubarb and mirabilite powder around the umbilicus to assist in the treatment of adhesive intestinal obstruction can improve intestinal inflammation, shorten the duration of abdominal pain relief, and expedite the onset of spontaneous gas and defecation, thus proving the treatment’s efficacy (Wang W. et al., 2018). *Jiawei Chengqi Decoction* can promote the recovery of gastrointestinal function, improve urodynamics, and improve the quality of life of patients after extensive hysterectomy with preservation of pelvic autonomic nerves, and the clinical efficacy is remarkable (Gao and Wang, 2015).

### 4.2 Clearing heat and purging fire for removing toxin

Rhubarb is known for its ability to clear heat, purge fire and detoxify, primarily due to its anti-pathogenic microorganism, anti-inflammatory and antitoxin pharmacological effects (Wang et al., 2019). In studies using a rat fever model and a rat foot swelling model, both raw and cooked rhubarb demonstrated an inhibitory effect on the increase of body temperature and swelling rate (Yang et al., 2011). The effects of rhubarb on inflammation were further analyzed using an oral ulcer model and a pneumonia model in rats, comparing the outcomes before and after wine-roasting (Wang Y. et al., 2015). Post wine-roasting, wine rhubarb significantly reduced the inflammation scores in rats with oral ulcers and was more effective in healing mucous membrane tissue damage. It also markedly decreased the number of leukocytes, the ratio of neutrophils and the level of TNF-α in the blood of pneumonia-afflicted rats, indicating that wine rhubarb has enhanced therapeutic effects on upper energizer diseases compared to its raw form.

In the management of inflammatory diseases, raw rhubarb is frequently used and proves effective in treating both acute and chronic inflammatory conditions. Topically applied raw rhubarb extract as an adjuvant treatment for severe periodontitis in diabetic patients improves clinical indices, demonstrates robust clinical efficacy, and maintains a favorable safety profile (Li et al., 2018). The combination of *Dahuang Mudan Decoction* (DMD) with antibiotics in treating pediatric periappendiceal abscesses significantly boosts the cure rate without causing liver injury, underscoring the safety of this therapy (Lin et al., 2023). Many preclinical studies have shown that DMD has functions such as balancing gut microbiota, restoring intestinal homeostasis, protecting intestinal mucosa, immune suppression and anti-inflammatory effects. It is commonly used in clinical practice to treat ulcerative colitis (Yang et al., 2023). In treating chronic pelvic inflammatory disease, DMD reduces leukocyte levels in the serum and effectively mitigates the inflammatory response, enhancing clinical outcomes (Chen, 2021). Nasal feeding of raw rhubarb as an adjunct treatment for patients with severe pancreatitis complicated by abdominal cavity infection notably improves patient conditions, reduces inflammatory factors, and aids in the regression of the disease (Xiang et al., 2021).

### 4.3 Cooling blood to stop bleeding

Rhubarb is recognized for its ability to cool blood and stop bleeding, famously characterized as “stopping bleeding without leaving stasis” (Wang et al., 2019). The hemostatic effects of rhubarb and its various concoctions were compared using two methods: the capillary glass tube method and the mouse tail-breaking method, with a gray correlation analysis used to investigate the spectral effect relationship. The results indicated that the hemostatic times for the raw rhubarb group (13.90 ± 1.97 min) and the rhubarb charcoal group (6.40 ± 1.45 min) were significantly shorter than those of the control group (16.10 ± 1.66 min). There was no notable difference in hemostatic times between the wine rhubarb group (15.00 ± 1.94 min) and the cooked rhubarb group (15.00 ± 1.94 min). Additionally, compared to the coagulation time of the control group (3.05 ± 0.72 min), the coagulation time for the rhubarb charcoal group (1.85 ± 0.63 min) was significantly shorter (Zhu et al., 2008).

Clinically, rhubarb powder is frequently used to treat upper gastrointestinal bleeding. Gastroscopic application of ultramicro rhubarb powder for acute non-variceal upper gastrointestinal bleeding has shown significant advantages in terms of hemostasis time and rebleeding rates compared to epinephrine preparations (He and Yin, 2014; Zhou et al., 2015). In TCM, the process of “stir-fry to a charcoal” is employed to enhance the hemostatic effect, and rhubarb charcoal is commonly used to treat hemorrhagic conditions. However, there are no clinical studies evaluating the hemostatic effectiveness of rhubarb charcoal alone or in combination with compound formulas.

### 4.4 Removing blood stasis and dredging meridians

The term “removing blood stasis and dredging meridians” involves using medication to eliminate blood stasis, enabling *qi* and blood to flow freely through the body’s meridians. A comparison of the effects of four rhubarb concoctions on activating blood circulation and removing blood stasis in an acute blood stasis rat model revealed varying degrees of improvement in fibrinogen (FIB), prothrombin time (PT), activated partial thromboplastin time (APTT), thrombin time (TT) and whole blood viscosity. The effects of wine rhubarb and cooked rhubarb were less pronounced than those of raw rhubarb, and charcoal rhubarb showed no significant impact on any indices (Sui et al., 2012). An evaluation using a blood bio-efficiency assay assessed the antiplatelet aggregation effects of 10 anthraquinone derivatives found in rhubarb. The results indicated that the blood-activating potency of rhein and emodin was significantly higher than that of aloe-emodin, chrysophanol and physcion, and was 5.02 and 5.15 times greater than that of aspirin, respectively, demonstrating a stronger antagonism to adenosine diphosphate (ADP)-induced platelet aggregation; The antiplatelet potencies of aloe-emodin-8-O-β-D-glucoside, rhein-8-O-β-D -glucoside, emodin-8-O-β-D -glucoside, chrysophanol-8-O-β-D -glucoside and physcion-8-O-β-D -glucoside were higher 4.13, 4.46, 9.31, 5.46 and 7.80 times than that of aspirin, respectively, which indicated that the five anthraquinone glucosides had a strong ability to antagonize ADP-induced platelet aggregation (Tan et al., 2018).

Among the different rhubarb concoctions, wine rhubarb and cooked rhubarb are most effective in activating blood circulation and removing blood stasis, making them commonly used in clinical settings for treating diseases associated with blood stasis. In traditional Chinese medicine processing theory, wine directs the medicine’s properties to the upper energizer, making wine rhubarb particularly effective for treating blood stasis in respiratory diseases. *Jiawei Xiayuxue Decoction* combined with acetylcysteine has improved alveolar diffusion function, exercise endurance and quality of life in patients with interstitial pulmonary fibrosis, while halting the progression of lung lesions (An et al., 2017).

In TCM, one of the primary pathological factors of tumor diseases is “blood stasis”. *Dahuang Zhechong Pill* (DZP), a classic formula for activating blood circulation and removing blood stasis that includes cooked rhubarb, underscores rhubarb’s primary clinical application in tumor treatment. Drug research has also found that DZP can exert anti-tumor effects by inhibiting cell proliferation, inducing cell apoptosis, regulating immune function, inhibiting angiogenesis, reducing metastasis and reversing cell drug resistance (Tian et al., 2023). Transcatheter arterial chemoembolization (TACE) is recognized as a safe and effective palliative treatment for intermediate and advanced hepatocellular carcinoma, yet postoperative complications can impact therapeutic efficacy (Lv et al., 2022). The combination of DZP with TACE in treating primary liver cancer significantly enhanced the effective rate to 88.99%, improved clinical indicators, raised the benefit rate to 94.87%, and markedly reduced the incidence of postoperative adverse reactions (Dai et al., 2021). Additionally, DZP combined with paclitaxel+cisplatin and olaparib has improved lymphocyte and serum tumor marker levels in patients with advanced ovarian cancer, enhancing patient quality of life and reducing adverse reaction incidences (Chen and Zhao, 2023).

Endometriosis (EMs) and adenomyosis (AM), although benign gynecological conditions, exhibit malignant behaviors such as implantation, infiltration, metastasis and recurrence (Xia et al., 2016). And TCM theory suggests that the core mechanism is “stagnation of blood stasis”. The Wine Rhubarb-Peach Seed herb pair, pivotal in many formulas for resolving blood stasis, has been studied by the author’s team for its effectiveness in treating AM. As a systematic and holistic medical research paradigm, network pharmacology helps to systematically reveal the biological basis of TCM, and has been widely applied in the research of bioactive compounds, syndromes, formulas and other topics of TCM (Wang and Li, 2022). Network pharmacology studies have suggested that this herb pair can target AM through multiple pathways, particularly through the P53 signaling and apoptosis pathways mediated by the expression of P53 and BAX (Liao et al., 2020). Additionally, this herb pair influences the kinetic characteristics of endometrial cells in mice with AM, inhibiting abnormal proliferation and reducing cell migration and invasiveness (Lei et al., 2023). The traditional hospital preparation *NeYiKangFu Tablet* (NYKF), featuring the Cooked Rhubarb-Peach Seed herb pair, has been widely used clinically to treat EMs and AM. NYKF promotes apoptosis and inhibits cell proliferation and migration in EMs by targeting the RAF kinase inhibitor protein (RKIP) to inhibit the RAF/MEK/ERK signaling pathway, as demonstrated in our animal experiments (Wen et al., 2022). DZP, whose core medication including Cooked Rhubarb-Peach Seed herb pair, has also been utilized in the treatment of EMs. Compared with mifepristone, DZP can better improve clinical symptoms and signs in patients, and its mechanism of action may be related to the regulation of prostaglandin levels (Li, 2013). On the basis of progestin therapy, the use of DZP in the treatment of EMs patients with pelvic pain can further reduce the symptoms associated with pelvic pain, improve the quality of life of the patients and clinical efficacy, and have the effect of regulating the levels of prostaglandins, matrix metalloproteinases and pro-inflammatory factors (Fan et al., 2019).

### 4.5 Promoting diuresis and descending jaundice

Promoting diuresis and descending jaundice are critical traditional effects of rhubarb, reflecting its therapeutic role in treating liver and gallbladder disorders. Rhubarb compound preparations have demonstrated effective treatment for common liver diseases such as jaundice hepatitis, non-alcoholic fatty liver disease and cirrhosis ascites. The combination of *Dahuang Lidan Capsule* and silymarin in treating non-alcoholic fatty liver disease has been shown to improve the liver function and lipid levels in patients, enhance clinical outcomes, and potentially regulate intestinal flora, which may be one of the mechanisms of its effectiveness (Li et al., 2021). YCHD combined with probiotics significantly ameliorates symptoms of non-alcoholic fatty liver disease, reduces levels of blood lipids and liver enzymes, prevents hepatocyte destruction, and notably decreases levels of lipopolysaccharide-binding protein (LBP), total bile acid (TBA) and TNF-α. This treatment also repairs intestinal mucosal barrier function, reduces the inflammatory response causing liver damage, and interrupts the liver-intestinal vicious cycle, thus protecting the liver (Gu, 2021). Furthermore, the oral administration of raw rhubarb powder as an adjuvant in the treatment of jaundice hepatitis significantly improved the pathological damage to the liver of patients and markedly reduces the levels of alanine aminotransferase (ALT) and total bilirubin (TB) in the liver tissue and serum, possibly through the modulation of the Fas/FasL system by rhubarb (Huang et al., 2008). However, the differences between various rhubarb concoctions in inducing diuresis and reducing jaundice have not been experimentally studied and warrant further investigation.

## 5 Toxicological studies

### 5.1 Toxicity of rhubarb

As research into the pharmacological activities of rhubarb deepens, attention has increasingly focused on its potential toxic and side effects. Current studies predominantly suggest that anthraquinones, the most abundant compounds in rhubarb, might be responsible for its toxicity, particularly causing hepatorenal issues during drug use. Emodin exhibits concentration and time-dependent cytotoxic effects on L-02 cells (Li C. L. et al., 2012) and can significantly disrupt cellular metabolism, including glutathione and fatty acid metabolism (Liu et al., 2015). *In vitro* studies have demonstrated that aloe-emodin may inhibit the proliferation of HL-7702 cells by inducing reactive oxygen species (ROS) production, leading to Fas-mediated death and mitochondrial pathway activation, thus causing cell cycle arrest, apoptosis and liver cell damage (Dong et al., 2017). Long-term, high-dose administration of rhein also shows notable toxicity to mouse kidneys, with more pronounced effects in males (Hu et al., 2019). The toxicity of total anthraquinone from rhubarb has a dose-effect relationship with HK-2 cells, directly inhibiting their proliferation, altering cell morphology, and at low dosages, causing advanced apoptosis and necrosis (Ren et al., 2015b). Total anthraquinone can also induce varying degrees of edema and necrosis in rat renal tubular epithelial cells (Ren et al., 2012).

The strong laxative effect of rhubarb frequently causes gastrointestinal reactions in clinical practice, with anthraquinone compounds primarily responsible for these effects (Qin et al., 2011). Concerns exist that excessive use of anthraquinones may lead to melanosis coli (Zhang et al., 2023) and potentially increase the risk of colon cancer. However, it has not been confirmed that long-term use of high-dose sennosides induces intestinal hyperplasia or increases the risk of melanosis coli and subsequent colon cancer (Le et al., 2021). Emodin, another compound, exhibits embryotoxic effects (Chang et al., 2012a; Chang et al., 2012b) that could damage embryonic development through caspase-dependent apoptosis, significantly reducing the maturation of oocytes, fertilization rates and *in vitro* embryo development. It may also induce apoptosis in the cell mass within the blastocyst and the trophoblast ectoderm in mice, resulting in decreased embryo development and survival rates. In addition, certain anthraquinones in rhubarb are known to cause cardiotoxicity (Li et al., 2022), phototoxicity, genotoxicity, reproductive toxicity and other adverse effects (Dong et al., 2016; Dong et al., 2020). Tannins in rhubarb also appear to demonstrate hepatorenal toxicity, evidenced by reduced liver transparency, liver atrophy, delayed absorption of the yolk sac, liver cell deformation, and apoptosis in juvenile zebrafish (Wang et al., 2022), as well as mild toxicity to HK-2 cells (Ren et al., 2015a).

Compared to anthraquinones, the toxicity of other rhubarb compounds such as tannins, stilbenes and phenylbutanones has been less studied, and most of them were *in vitro* and *in vivo*, although this is also a crucial step for the clinical application of rhubarb. Therefore, further research on compounds other than anthraquinones and well-designed clinical studies are needed to more comprehensively understand the toxicological effects of rhubarb on the human body.

### 5.2 Effects of processing on toxicity

The *Jingyue*’*s Complete Book (Jingyue QuanShu)* and *Convenient Reader on Materia Medica (Bencao Biandu)* note that rhubarb is inherently cold, with a strong smell and flavor, classified as deeply yin, and is toxic. However, it is also acknowledged that processing rhubarb or combining it with other Chinese medicinal herbs can mitigate its intense properties. Indeed, the processing of Chinese medicinal herbs has been shown to reduce toxicity to some extent. There is experimental evidence that “7 times steaming” cooked rhubarb nearly eliminates the hepatotoxicity seen in raw products (Sim et al., 2015). A study investigated the relationship between detoxification processes and changes in rhubarb’s compounds, establishing a correlation sequence for major compounds associated with hepatorenal toxicity: total combined anthraquinones> total tannins> total free anthraquinones (Wang et al., 2009). While raw rhubarb can harm the liver and kidneys in mice, processed rhubarb diminishes this toxicity, likely due to reduced levels of combined anthraquinones and tannins. Further research in an acute toxicity study on zebrafish demonstrated that, compared to liver degeneration observed in the group treated with raw rhubarb, the group treated with cooked rhubarb exhibited only mild liver degeneration at the same high concentration. The maximum non-lethal concentration of cooked rhubarb was significantly higher, about nine times that of raw rhubarb, indicating a substantial detoxification effect (Wang et al., 2022). After processing, the impact of rhubarb’s “bitter cold” on the stomach is significantly reduced, lessening gastric mucosa damage and gastrointestinal dysfunction in rats (Zhang et al., 2019). The study on the toxicity of different rhubarb concoctions on *Tetrahymena thermophila* BF5, found that the inhibitory effect on growth was in the order of raw rhubarb> wine rhubarb> cooked rhubarb> rhubarb charcoal, suggesting significant detoxification post-processing (Li et al., 2012b). Another research also demonstrated that both steaming and vinegar steaming effectively reduce the genotoxicity of rhubarb (Zou et al., 2011).

Multiple toxicological studies align with compounds analyses pre- and post-processing, suggesting that processing can detoxify by reducing large molecule compounds like anthraquinones and tannins. However, the increased content of free anthraquinones such as emodin, rhein, aloe-emodin, physcion and chrysophanol post-processing presents a contradiction. An *in vivo* study examined the distribution of free anthraquinones in rat tissues after administration of raw and cooked rhubarb extracts, revealing that despite higher levels of free anthraquinones in cooked rhubarb, the concentrations of rhein, emodin and aloe-emodin, particularly in liver and kidney tissues, were significantly decreased (Fang et al., 2011). This suggests that processing not only alters the content but may also modify the efficacy and reduce toxicity by influencing the distribution and action of its compounds within the body, providing a scientific foundation for “processing attenuating toxicity”. Nonetheless, more pharmacokinetic studies on the compounds of rhubarb pre- and post-processing are necessary to further elucidate the mechanisms of processing.

## 6 Summarizing and looking forward

Rhubarb is renowned for its diverse pharmacological effects. The TCM technique of processing can alter the composition of its compounds to enhance its efficacy, modify its medicinal properties, and reduce its toxicity. The effectiveness of rhubarb and its preparations in treating diseases has been well-documented through long-standing practice in Chinese medicine, highlighting its significant clinical value. Raw rhubarb is characterized as bitter, cold and sedative, possessing strong, harsh medicinal properties with potent laxative effects. Wine rhubarb, also bitter and cold, has a slightly slower laxative effect and is effective at clearing heat and toxins from the upper energizer. Cooked rhubarb, with reduced laxative strength, enhances blood circulation and removes blood stasis, making it particularly useful in oncology. Rhubarb charcoal, with minimal laxative properties, is primarily used to cool blood and stop bleeding. Although numerous studies have investigated the changes in active compounds due to various rhubarb processing, discrepancies in processing temperature, duration, and solvents across studies introduce variability in the outcomes. This variability complicates the consistency of the changes in active compounds after processing, impacting their clinical applications. Implementing strict and standardized processing procedures and compound identification standards is crucial to address these issues. Moreover, there is a shortage of high-quality clinical studies on the active monomers, whole drug and compound formulations of rhubarb, which hampers the development of systematic medication regimens and clinical promotion for rhubarb and its concoctions. As a multi-potency, multi-targeted Chinese medicine, ongoing pharmacological research on various concoctions of rhubarb will facilitate the standardization of its processing and clinical use, enhance our understanding of its therapeutic efficacy, and broaden the spectrum of treatable diseases with rhubarb. It is also conducive to further exploration of the medicinal value of rhubarb, improve its therapeutic effect, and provide more valuable basis for clinical application.
