# Supplementary material for: The application of rhubarb concoctions in traditional Chinese medicine and its compounds, processing methods, pharmacology, toxicology and clinical research
Source: Front Pharmacol. 2024 Aug 7;15:1442297. doi: 10.3389/fphar.2024.1442297 (PMC11335691; doi:10.3389/fphar.2024.1442297)
Supplement: Supplementary file 1 [file Table1.DOCX]

Supplementary Material

# Supplementary Tables

**Supplementary Table 1 The origin and development of rhubarb processing.**

| **Age** | **Main processing method** | **Source** |
| --- | --- | --- |
| Eastern Han Dynasty  25-220 AD | Peeling; wine washing; wine soaking | *Golden Chamber Jade Letter Classic*  *(Jingui Yuhan Jing)* |
|  | steaming | *Synopsis of Golden Chamber*  *(Jingui Yaolve)* |
|  | Wrap ten layers of wet paper and simmer | *Zhongzang Classic*  *(Zhong Zang Jing)* |
| Wei-Jin Dynasties  AD 220-420 | Cut into pieces of chess size and stir fry together with shaosu, allowing the shaosu to blend into rhubarb. Do not stir fry until yellow or black, as it will lose its medicinal effect | *The Handbook of Prescriptions for Emergencies*  *(Zhouhou Beiji Fang)* |
| Northern and Southern Dynasties  420-589 AD | After cutting, steam and dry, then sprinkle melted wax and steam seven times, dry, then sprinkle diluted honey water and steam until the section is black, dry | *Master Lei's Discourse on Drug Processing*  *(Lei Gong Pao Zhi Lun)* |
| Tang Dynasty  AD 618-907 | Roast until black; Boil with wine | *Valuable Prescriptions for Emergency*  *(Beiji Qianjin Yaofang)* |
|  | Soak in wine and stir-fry; Stir-fry with wine; Steam with wine | *Essence of the Silvery Sea*  *(Yin Hai Jing Wei)* |
|  | Roast until smoke comes out; Cut into thin slices and steam under rice before drying in the sun | *Supplement to Valuable Prescriptions*  *(Qianjin Yifang)* |
|  | Fry with vinegar | *Materia Medica for Dietotherapy*  *(Shi Liao Ben Cao)* |
|  | Boil with rice vinegar; Roast until it turns yellow black; Steamed under rice; fry | *Medical Secrets of an Official*  *(Wai Tai Mi Yao)* |
| Song Dynasty  960-1279 AD | Wrap with wet paper and simmer until the paper turns yellow; Soak in rice vinegar for two nights, carefully chop with a bamboo knife, steam nine times on a tiled cookware, and grind into a paste | *Widespread Treatment Prescriptions*  *(Bo Ji Fang)* |
|  | Soak in urine for seven days, change fresh urine every day, wrap in wet paper, simmer, cut into pieces, and bake | *Medical Prescriptions Collected by Su Shi and Shen Kuo*  *(Su Shen Liang Fang)* |
|  | Soak in rice washed water for a meal time, then drain and stir fry over low heat until cooked | *Book of Nanyang for Life Saving*  *(Lei Zheng Huo Ren Shu)* |
|  | Apply ginger juice onto rhubarb and bake until dry, then mash into small pieces; Cut into pieces and soak in honey overnight before baking; Cut and soak in vinegar, then stir fry over low heat until cooked; Stir fry with vinegar until it turns purple; Steam with wine and cut into pieces for grilling; After steaming, sun dry and repeat nine times | *General Records of Holy Universal Relief*  *(Sheng Ji Zong Lu)* |
|  | Soak in wine and slice the rhubarb into slices, paste the peeled croton beans on the rhubarb. Wrap it in paper and steam it three times over the rice. Cut and stir fry until it turns a burnt yellow color. Remove the croton beans and use it | *Key to Therapeutics of Children's Diseases*  *(Xiaoer Yaozheng Zhijue)* |
|  | Bury in hot ash and burn until cooked; Baking | *Prescriptions Collected by the Public Pharmacy*  *(Tai Ping Hui Min He Ji Ju Fang)* |
|  | Burn until the exterior is burnt black and the interior is burnt yellow. The surface is partially carbonized but the inner layer retains its original flavor | *General and Micro Health Prescriptions for Children*  *(Xiaoer Weisheng Zongwei Lunfang)* |
|  | Simmer with wheat bran and steam; Steaming with honey | *Treatise on Three Categories of Pathogenic Factors*  *(San Yin Ji Yi Bing Zheng Fang Lun)* |
| Yuan Dynasty  1206-1368 AD | Soak in rice washed water for three days, replace it with fresh rice washing water every day, and then bake it to shreds; Wrap in vinegar soaked paper and simmer, then cut into pieces; Soak in wine together with Sanleng for a night and bake | *Precious Mirror for Health*  *(Weisheng Baojian)* |
|  | Remove the rough skin, soak in wine for four to six hours, wrap in paper, and simmer | *Empirical Recipes of Ruizhu Hall*  *(Rui Zhu Tang Jing Yan Fang)* |
|  | Wrap it in wet paper and steam it under the rice. After the rice is cooked, remove the paper and cut it into pieces for baking | *Effective Formulae Handed Down for Generations*  *(Shi Yi De Xiao Fang)* |
|  | Wrap it in wet paper and simmer it, do not simmer it too much. Cut it and bake it dry, then add wine to moisten it. Stir fry until cooked and dry; Steam on rice and sun dry it; Wrap in dough and simmer until it cooked and crushed | *Danxi’s Mastery of Medicine*  *(Danxi Xinfa)* |
|  | Burn until the surface is charred black on the outside, but the inner layer retains its original flavor. Grind it into an extremely fine powder, wrap it in paper, and cover it with a bowl on the ground overnight to remove the fire toxins | *Miraculous Book of Ten Medicines*  *(Shiyao Shenshu)* |
| Ming Dynasty  1368-1644 AD | Cut into small pieces and place them in a sand container, add water and stir 81 times. Take the upper suspension and filter it for use; Boil with rice vinegar after peeling until soft and tender; Soak in good wine overnight and drain to dry in the sun | *Medical Compendium*  *(Yi Xue Gang Mu)* |
|  | Wrap with dough and simmer for a while, then remove the dough and bake; Add rice vinegar to boil into paste, prepare brick and tile powder on the new bricks, pour the boiled rhubarb paste on it, place it outdoors in the hottest time of summer, until it completely dry and grind it into powder | *Prescriptions for Universal Relief*  *(Pu Ji Fang)* |
|  | Steam after soaking in wine; Stir with wine until the wine evaporates completely | *Overview of benevolent treatment methods*  *(Ren Shu Bian Lan)* |
|  | Soak rhubarb in water soaked with safflower for a day to drain; Soak rhubarb in little boy's urine with some salt added for a day and take it out to dry; Soak the rhubarb and Angelica sinensis together in diluted vinegar for a day, then remove the rhubarb and dry | *Supplement to Ten Thousand Diseases and Revival*  *(Zengbu Wanbing Huichun)* |
|  | Add wine and stir, steam and dry, repeat nine times, then grind it into powder | *Forbidden Prescriptions of Lu Mansion*  *(Lu Fu Jin Fang)* |
|  | Slice the Sichuan Coptis chinensis with the root hair removed, soak in water and use the solution stir together with the sliced rhubarb, fry dry; Remove the stem of Evodia rutaecarpa，soak in water and use the solution stir together with the sliced rhubarb, Fry until dry | *Prolonging Life and Preserving the Origin*  *(Shou Shi Bao Yuan)* |
| Qing Dynasty  1616-1911 AD | Stir fry with ginger juice; Stir with leek juice and dry in the sun | *Overview of Treatment by Various Medical Practitioners*  *(Yi Zong Shuo Yue)* |
|  | Soak in vinegar and sun dry, repeat nine times; Steam with wine until it turns black | *Compendium of Surgery*  *(Wai Ke Da Cheng)* |
|  | Stir fry with leek juice until it turns black | *Encountering the Sources of the "Classic of Materia Medica"*  *(Ben Jing Feng Yuan)* |
|  | Steam three times and then dry in the sun; After soaking in vinegar, wrap it in paper and place it in hot ash until it cooked | *Collection of Good Prescriptions*  *(Liang Peng Hui Ji)* |
|  | Boil with aged wine until it soft and tender, then dry it in the sun | *Life-saving Manual of Diagnosis and Treatment of External Diseases*  *(Wai Ke Quan Sheng Ji)* |
|  | Slice and stir fry with lime in a pot. Take it out when the lime turns peach red, remove the rhubarb, and leave the left part on the ground overnight. Grind it into powder to use; Soak thoroughly in wine and steam in a bowl over water until it cooked and soft, then take it out and mash | *Complete Book of Syndromes and Treatment of Surgery*  *(Waike Zhengzhi Quanshu)* |
|  | Boil rhubarb with gaomi vinegar until thick, sun dry and crush, then add vinegar and boil over low heat, repeat three times, sun dry and grind it into powder | *Medical Cases of Wu Jutong*  *(Wu Ju Tong Yi An)* |
