# Supplementary material for: The application of rhubarb concoctions in traditional Chinese medicine and its compounds, processing methods, pharmacology, toxicology and clinical research
Source: Front Pharmacol. 2024 Aug 7;15:1442297. doi: 10.3389/fphar.2024.1442297 (PMC11335691; doi:10.3389/fphar.2024.1442297)
Supplement: Supplementary file 2 [file Table8.docx]

Supplementary Material

# Supplementary Tables

# Supplementary Table 8 Effect of rhubarb concoctions on traditional pharmacology.

| **Efficacy** | **Processed products of rhubarb** | **Cells/Animals and weight** | **Controls** | **Modeling method** | **Dose (concentration) and administration method** | **Duration of administration** | **Results** | **References** |
| --- | --- | --- | --- | --- | --- | --- | --- | --- |
| Purgation to Eliminate Accumulation | Raw rhubarb, Wine rhubarb, Cooked rhubarb, Rhubarb charcoal. | Kunming breeder mice, half male and half female, weighing 20-22g. | Distilled water | — | High dose 5g/kg  Low dose 3g/kg  Volume 30mL/kg | 5h | The four processed products of rhubarb all have certain laxative effects, but the intensity of laxative action, first laxative time, frequency of laxative action, total defecation frequency, and promotion effect on the small intestine are all different. Raw rhubarb has the strongest laxative effect, followed by wine rhubarb, while cooked rhubarb and rhubarb charcoal have the weakest effect. | (Yan et al., 2010) |
|  |  |  | Distilled water | — |  | 30min |  |  |
|  | Raw rhubarb, Wine rhubarb, Cooked rhubarb, Rhubarb charcoal. | Kunming breeder mice, half male and half female, weighing 20-22g. | Distilled water | — | High dose 5g/kg  Low dose 3g/kg  Volume 20mL/kg | 5h | The extracts of the four processed products of rhubarb all have certain laxative effects, but the intensity of the laxative effect varies. Raw rhubarb has the strongest laxative effect, followed by wine rhubarb, while cooked rhubarb and rhubarb charcoal have the weakest effect. | (Li et al., 2011b) |
|  |  |  | Distilled water | — |  | 30min |  |  |
|  |  | Healthy SD rats, male, weighing 180-200g. | Distilled water | — |  | 5d |  |  |
|  | Raw rhubarb, Wine rhubarb, Cooked rhubarb, Rhubarb charcoal. | Healthy ICR mice, weighing 16-18g, male. | Rh03 rhubarb sample at high and low doses (dose spacing of 1:0.7) | Compound Diphenoxylate Tablets (50mg/kg) by gavage. | High dose 2000U/kg  Low dose 1200U/kg | 10h | After processing, the laxative potency of raw rhubarb significantly decreased, while the laxative activity of wine rhubarb was weaker than that of raw rhubarb, followed by cooked rhubarb, and rhubarb charcoal lost its laxative activity. | (Li et al., 2012c) |
|  | Raw rhubarb, Cooked rhubarb. | Kunming clean grade mice, 18-22g, male. | Normal saline | Traditional Chinese medicine (Aconite, Dried Ginger, Cinnamon, Fructus Evodiae, Pepper, etc.) combined with loperamide hydrochloride orally administered to create a model, 0.2mL/10g (40g of raw medicine/kg, 4 mg of loperamide hydrochloride/kg), once a day, continuously administered for 15 days. | Raw rhubarb 5.00, 2.50, 1.25g/kg  Cooked rhubarb 5.00, 2.50, 1.25g/kg  Tong Bian Ling Capsules 1g/kg | 3h  30min | There are significant differences in the regulatory effects of raw and cooked rhubarb on gastrointestinal hormones and intestinal neurotransmitters. | (Wu et al., 2014) |
|  |  | Healthy Wistar rats, male, weighing 220 ± 20g. | Normal saline | Traditional Chinese medicine (Aconite, Ginger, Cinnamon, Fructus Evodiae, Pepper, etc.) combined with Loperamide Hydrochloride by gavage for modeling, 2mL/100g (20g raw medicine/kg, 2mg Loperamide Hydrochloride/kg), once a day, continuously administered for 28 days. | Raw rhubarb 1. 25g/kg  Cooked rhubarb 1. 25g/kg  Tong Bian Ling Capsules 11g/kg | 5d |  |  |
| Clearing Heat and Purging Fire for Removing Toxin | Raw rhubarb, Wine rhubarb, Cooked rhubarb. | Kunming breeder mice, male, weighing 17-20g. | A 1:1 mixture of sodium carboxymethyl cellulose and Indian ink | — | Crude drug 10g/kg | Single-dose administration | Different processed rhubarb have obvious laxative effects, but their laxative effects are weakened after processing. Different processed rhubarb have obvious antipyretic and anti-inflammatory effects. | (Yang et al., 2011) |
|  |  | Wistar rats, male, weighing 180-200g. | Not specifically mentioned | Immediately after administration, 10mL/kg of 20% yeast suspension was subcutaneously injected into the back of each mouse. | Raw rhubarb, Wine rhubarb, Cooked rhubarb 5.6g/kg  Positive drug aspirin 150mg/kg | Single-dose administration |  |  |
|  |  |  | Sodium carboxymethyl cellulose | After 30 minutes of administration in each group, 0.1mL of 1% carrageenan suspension was subcutaneously injected into the right hind foot of each mouse. | Raw rhubarb, Wine rhubarb, Cooked rhubarb 5.6g/kg  Positive drug dexamethasone acetate tablets 20mg/kg | 30min |  |  |
|  | Raw rhubarb, Wine rhubarb. | SPF grade SD rats, male, with a body weight of 250 ± 20g. | Normal saline | 40% acetic acid solution is vertically placed on the inner oral mucosa of rats and burned for 30 seconds. After 24 hours, oral ulcers can form. | 3g/kg | 3d | After being roasted in alcohol, the therapeutic effect of rhubarb on upper-jiao syndrome is enhanced. The inhibitory effect on the activity of energy metabolism enzymes in the liver tends to weaken. | (Wang et al., 2015d) |
|  |  |  | Normal saline | Intraperitoneal injection of 10% chloral hydrate anesthesia, inserting a sterilized silicone tube into the trachea, immediately using a microinjector to draw 0.02mL of bacterial fluid into the tube, keeping the rat in place for 10 minutes, allowing the bacterial fluid inside the tube to reach the bronchi and alveoli under gravity, leading to pulmonary infection. | Raw rhubarb, Wine rhubarb 3g/kg  Positive drug cefixime 10mg/kg | Start gavage administration 3 days before modeling, once a day, until the 6th day after modeling |  |  |
|  |  | Healthy and clean ICR mice, male, with a body weight of 18 ± 2g. | Normal saline | — | Raw rhubarb and wine rhubarb decoction, once per day, 5 g/kg | 10d |  |  |
| Cooling Blood to Stop Bleeding | Raw rhubarb, Wine rhubarb, Cooked rhubarb, Rhubarb charcoal. | Kunming breeder mice with half male and half female, weighing 20 ± 2g. | Normal saline | — | 1.75g/kg | Single-dose administration | Among different processed products of rhubarb, raw rhubarb has the strongest laxative effect, while cooked rhubarb and wine rhubarb have a slightly slower effect. Rhubarb charcoal has an antidiarrheal effect. Among different processed products of rhubarb, rhubarb charcoal has the strongest hemostatic effect, followed by raw rhubarb, while cooked rhubarb and wine rhubarb have no significant hemostatic effect. There are differences in the laxative and hemostatic effects of different processed groups of rhubarb. | (Zhu et al., 2008) |
| Removing Blood Stasis and Dredging Meridians | Raw rhubarb, Wine rhubarb, Cooked rhubarb, Rhubarb charcoal. | SD rats, weighing 220-240g, half male and half female. | Cold boiled water | Subcutaneous injection of 0.1% adrenaline hydrochloride (0.06mg/100g) twice, with a 4-hour interval between the two injections. The rats were placed in ice water (4℃) and soaked in cold water for 5 minutes. | 3.5g/kg, 2ml/100g, twice a day in the first two days and once a day in the last five days | 7d | Except for rhubarb charcoal, all processed products of rhubarb have certain blood activating effects on rats with blood stasis, but their effects on various indicators of blood activating and stasis resolving are different, reflecting the characteristic of multiple effects of traditional Chinese medicine components through multiple parts and targets. At the same time, it also indicates that different processing methods may form differences in the medicinal properties of each processed product by changing the material basis of rhubarb, and ultimately display different biological activities. | (Sui et al., 2012) |
