# Supplementary material for: The application of rhubarb concoctions in traditional Chinese medicine and its compounds, processing methods, pharmacology, toxicology and clinical research
Source: Front Pharmacol. 2024 Aug 7;15:1442297. doi: 10.3389/fphar.2024.1442297 (PMC11335691; doi:10.3389/fphar.2024.1442297)
Supplement: Supplementary file 3 [file Table7.docx]

Supplementary Material

# Supplementary Tables

**Supplementary Table 7 Experimental study on modern pharmacological effects of rhubarb.**

| **Pharmaco-logical effects** | **Varieties of rhubarb** | **Rhubarb extract (withdrawal method)/ Phytochemical components** | **Controls** | **Cells/Animals** | **Model** | **Dose (concentration), administration method** | **Duration of administration** | **Minimum active dose (concentration)** | **Results** | **Ref** |
| --- | --- | --- | --- | --- | --- | --- | --- | --- | --- | --- |
| Regulation of gastrointestinal function | / | Rhubarb extract | Negative: - | Male Wistar rats  (10 weeks old) | / | 1g/kg body weight  Oral administration | 0h/  2h/  5h/  8h | / | Sennoside A decrease AQP3 expression in the colon to inhibit water transport from the luminal to the vascular side, leading to a laxative effect. The decreases in the levels of AQP3 are caused by rheinanthrone, which is a metabolite of sennoside A, this metabolite activates the macrophages in the colon and increases the secretion of PGE2; PGE2 acts as a paracrine factor and decreases AQP3 expression in colon mucosal epithelial cells. | (Kon et al., 2014) |
|  |  |  | Positive:  sennoside A  (50 mg/ kg  body weight) |  |  |  |  |  |  |  |
|  |  | Sennoside A/ rheinanthrone/ rhein | Negative: - | HT-29 cells | / | Sennoside A  (200μM)  Rheinanthrone  (10μM)  Rhein  (200μM) | 15min/5h | / |  |  |
|  |  | Sennoside A/ rheinanthrone/ rhein | Negative: - | Raw264.7 cells | / | Sennoside A  (100 or 200 μM)  Rheinanthrone  (2 or 10 μM)  Rhein  (100 or 200 μM) | 30min | / |  |  |
|  | *Rheum palmatum L.* | Rhubarb free anthraquinones (RhA) (by 70% ethanol) | Negative: Mod | Male Sprague-Dawley rats (six weeks old, 200 ± 20 g) | Constipation | 100 mg/kg·d,  300 mg/kg·d,  500 mg/kg·d | 3d | 300 mg/kg | RhA upregulated VIP expression, activated the cyclic adenosine monophosphate protein kinase A (cAMP/PKA) pathway, and further stimulated CFTR expres­sion while inhibiting NHE3 and ENaC expression, resulting in a hypertonic state in the colonic lumen. Water transport could then be driven by an osmotic gradient, which in turn led to the upregulation of AQP3, AQP4, and AQP8 expression. RhA likely improved gastrointestinal motility by increasing serum VIP, SP, and MTL concentrations, thus promoting faecal excretion. | (Lv et al., 2024) |
|  |  |  | Positive:  Polyoxyethylene 4000  (1800 mg/kg) |  |  |  |  |  |  |  |
|  |  | RhA, aloe-emodin, rhein, emodin, chrysophanol, physcion | Negative: saline | Male Sprague-Dawley rats (six weeks old, 200 ± 20 g) | Constipation | RhA  (300 mg/kg·d)  Aloe-emodin  (300 mg/kg·d)  Rhein  (300 mg/kg·d)  Emodin  (300 mg/kg·d)  Chrysophanol  (300 mg/kg·d)  Physcion  (300 mg/kg·d) | 3d | / |  |  |
|  | *Rheum tanguticum Maxim. ex Balf.* | Rhubarb extract  (water) | Negative: - | Male Sprague-Dawley rats (5-week-old, 170 ± 20 g) | Constipation | 1.0 g/kg·d,  3.0 g/kg·d,  9.0 g/kg·d | 10d | / | Rhubarb could significantly shorten gastrointestinal transit time, increase fecal water content and defecation frequency, improve gastrointestinal hormone disruption, and protect the colon mucus layer. Rhubarb could improve the disorder of intestinal microbiota in constipated rats. The fecal metabolic profiles of constipated rats were improved by rhubarb. The amelioration of rhubarb in constipation might modulate the intestinal microflora and its metabolism. | (Yang et al., 2022) |
|  |  |  | Negative: models |  |  |  |  |  |  |  |
|  |  |  | Positive: bisacodyl |  |  |  |  |  |  |  |
| Anti-bacterial | / | Rhubarb suspension  (water) | / | *Methicillin-resistant staphylococcus aureus* (MRSA)  *Methicillin-sensitive staphylococcus aureus* (MSSA)  *Staphylococcus aureus* ATCC 29213 | / | 256/ 128/ 64/ 32/ 16/ 8/ 4/ 2/ 1/ 0.5/ 0.25/ 0.125 mg/mL  10 μL | 18h | MRSA:16-128mg/ml  MIC 50:32 mg/ml  MIC 90:64mg/ml  MSSA:16-64 mg/ml  MIC 50:32 mg/ml  MIC 90:64 mg/ml | The inhibitory effect of rhubarb on Staphylococcus aureus is obvious, and the mechanism of action is by destroying the integrity of the bacterial cell wall and the cell membrane. | (Xu et al., 2021) |
|  | / | Rhein-8-O-β-D-glucopyranoside, emodin-8-glucoside, chrysophanol, aloe-emodin, gallic acid, rhein, emodin, physcion | / | *Methicillin-resistant staphylococcus aureus* (MRSA)  *Methicillin-sensitive staphylococcus aureus* (MSSA)  *Staphylococcus aureus* ATCC 29213 | / | 256/ 128/ 64/ 32/ 16/ 8/ 4/ 2/ 1/ 0.5/ 0.25/ 0.125 mg/mL  10 μL | 18h | Rhein-8-O-β-D-glucopyranoside: 3.2mg/ml  Emodin-8-glucoside: 3.2mg/ml  Chrysophanol: 1.6mg/ml  Aloe-emodin: 0.1mg/ml  Gallic acid: 0.2mg/ml  Rhein: 0.05mg/ml  Emodin: 0.025mg/ml  Physcion: 1.6mg/ml |  |  |
|  | / | Rhubarb extract  (water) | Negative: - | *Streptococcus suis strain* ATCC 700794 | / | 1/2, 1/4, 1/8, or 1/16 × MIC  100 μL | 24h/ 48h/ 72h | the MIC of rhubarb water extracts against *S.suis* ATCC 700794was determined as 1.56 mg/ml | Rhein could not directly inactivate C. trachomatis but could inhibit the growth of C. trachomatis by regulating pathogen-host cell interactions. | (Ding et al., 2017) |
|  | / | Rhein | Positive: DMSO | HeLa cell | *Chlamydia trachomatis* infection | 40 μM | 1h | / | Rhein could not directly inactivate C. trachomatis but could inhibit the growth of C. trachomatis by regulating pathogen-host cell interactions. Combined with azithromycin, the inhibitory effect of rehin was synergistic both in vitro and in vivo. | (Yu et al., 2022) |
|  | / | Rhein | Negative: - | Female BALB/c rats (4-6week old) | Chlamydia  trachomatis  infection | 120 mg/kg·d rhein  1.0 mg/kg·d AZM  120 mg/kg rhein and 1.0 mg/kg AZM | 7d | / |  |  |
|  |  |  | Positive: DMSO |  |  |  |  |  |  |  |
| Anti-inflammatory | / | Rhein | Negative: - | Zebrafish | Inflammation | 1/ 5/ 20 μM | 6h | / | Tail-cutting-induced migration of immune cells was significantly reduced in transgenic zebrafish treated with rhein. The production of proinflammatory cytokines, including IL-6, IL-1β, and tumor necrosis factor-α, were significantly reduced in lipopolysaccharide (LPS)-induced RAW264.7 macrophages treated with rhein. Parallel to the inhibition of proinflammatory cytokines, rhein significantly reduced phosphorylation levels of NF-κB p65 and inducible nitric oxide synthase, as well as COX-2 protein expression levels. Furthermore, rhein significantly reduced NALP3 and cleaved IL-1β expression in LPS + ATP-induced RAW264.7 macrophages. Rhein may exhibit its anti-inflammatory action via inhibition of NF-κB and NALP3 inflammasome pathways. | (Ge et al., 2017) |
|  | / | Rhein | Negative: - | Raw264.7 cell | LPS-induced inflammation | 1/ 5/ 20 μM | 24h | / |  |  |
|  | / | Emodin | Negative: - | Raw264.7 cell | LPS-induced inflammation | 1/ 5/ 20 μM | 4h | / | LPS-induced the up-regulation of ICAM-1, MCP-1 and TNF-α, LPS-induced the down-regulation of PPARγ, and LPS-enhanced NF-κB p65 activation and DNA binding activity were substantially suppressed by emdoin in RAW264.7 cells.These effects of emdoin were largely abrogated by siRNA-PPARγ transfection. LPS-induced inflammation was potently compromised by emodin very likely through the PPARγ-dependent inactivation of NF-κB in RAW264.7 cells. | (Zhu et al., 2016b) |
|  | / | Chrysophanol | Negative: - | Raw264.7 cell | LPS-induced inflammation | 5 μM/ 10 μM/ 15 μM | 24h | / | LPS induced the up-regulation of TNF-α, IL-1β, iNOS and NF-κB p65, the down-regulation of PPAR-γ were substantially suppressed by chrysophanol in RAW264.7 cells. These effects of chrysophanol were largely abrogated by PPAR-γ inhibitor GW9662. LPS-induced inflammation was potently compromised by chrysophanol very likely through the PPAR-γ-dependent inactivation of NF-κB in RAW264.7 cells. | (Wen et al., 2018) |
|  |  |  | Negative: 0.2 μg/ml LPS |  |  |  |  |  |  |  |
|  |  |  | Positive: 10mM rosiglitazone |  |  |  |  |  |  |  |
|  |  |  | Negative: 10mM GW9662 +LPS |  |  |  |  |  |  |  |
| Regulation of coagulation | *Rheum palmatum L.* | Rhubarb extract  (water) | Negative: - | Male SD rats (8-10 weeks old, 230 ± 20 g) | Hyperviscosity syndrome | 0,0.21 g/kg·d, 0.42 g/kg·d, 0.84 g/kg·d  Phy: 50 mg/kg·d, 100 mg/kg·d  Emo: 50 mg/kg·d, 100 mg/kg·d | 7d | / | Middle dose of rhubarb (0.42 g/kg/d) significantly ameliorated pathological changes, hemorheology parameters, as well as levels of representative biomarkers such as plasma P-selectin (P-sel) and thromboxane (TXB2) in platelet activation compared to HVS rat model, whose effects were comparable to the positive drug aspirin or even better. F2 and FGG as the major effective targets of rhubarb as well as its two active ingredients Emo and Phy in PBRB. | (Gao et al., 2020) |
|  | *Rheum tanguticum Maxim.ex Balf.* of four areas | Rhubarb extract  (water) | Negative: saline | Half male and half female Kunming breeder rats (6 weeks old,  20.00±2.03 g) | / | 0.2mL/10g·d | 7d | / | The four main producing areas of Qinghai Rheum tanguticum Maxim.ex Balf. have obvious hemostatic and increase platelet effect, hemostatic effect from strong to weak order: Guoluo rhubarb, Qilian rhubarb, Huangnan rhubarb and Qunjia rhubarb. | (Wang et al., 2015c) |
| Digestive system protection | / | Yinchenhao  Decoction extract  (by 70% ethanol) | Negative: - | Male Wistar rats (200-250 g) | Hepatic fibrosis | 3.15 g/kg·d | 21d | / | Yinchenhao Decoction may attenuate liver fibrosis partially by regulating the targets in apoptosis-related TNF, PI3K-Akt and MAPK signaling pathways, subsequently promoting HSCs apoptosis and reducing HPCs apoptosis. | (Cai et al., 2019) |
|  |  |  | Negative: models |  |  |  |  |  |  |  |
|  | / | Emodin | Negative: - | Male Balb/c rats (6-8 weeks old) | Liver damage | 1.5625 mg/kg, 3.125 mg/kg, 6.25 mg/kg, 12.5 mg/kg, 25 mg/kg, 50 mg/kg body weight | 12h | / | Pretreatment with emodin significantly protected the animals from T cell-mediated hepatitis, as shown by the decreased elevations of serum alanine aminotransferase (ALT) and aspartate aminotransferase (AST), as well as reduced hepatic necrosis. Emodin pretreatment markedly reduced the intrahepatic expression of pro-inflammatory cytokines and chemokines, including tumor necrosis factor (TNF)-α, interferon (IFN)-γ, interleukin (IL)-1β, IL-6, IL-12, inducible nitric oxide synthase (iNOS), integrin alpha M (ITGAM), chemokine (C-C motif) ligand 2 (CCL2), macrophage inflammatory protein 2 (MIP-2) and chemokine (CXC motif) receptor 2 (CXCR2). Emodin pretreatment dramatically suppressed the numbers of CD4(+) and F4/80(+) cells infiltrating into the liver as well as the activation of p38 MAPK and NF-κB in Con A-treated mouse livers and RAW264.7 and EL4 cells. | (Xue et al., 2015) |
|  |  |  | Negative: models |  |  |  |  |  |  |  |
|  |  |  | Positive: emodin (50 mg/kg body weight) without models |  |  |  |  |  |  |  |
|  | / | Emodin | Negative: - | Male C57BL/6 rats (20-25 g) | Acute pancreatitis | 2.5 mg/kg·d | 48h | / | Emodin can significantly reduce the expression of TNF-α, DAO and IL-10, alleviate inflammatory stress and oxidative stress, and at the same time regulate the balance of Treg/Th17 and improve the immune disorders, so as to play a therapeutic role in the treatment of severe acute pancreatitis. | (Wu et al., 2019) |
|  |  |  | Negative: models |  |  |  |  |  |  |  |
