# Supplementary material for: The application of rhubarb concoctions in traditional Chinese medicine and its compounds, processing methods, pharmacology, toxicology and clinical research
Source: Front Pharmacol. 2024 Aug 7;15:1442297. doi: 10.3389/fphar.2024.1442297 (PMC11335691; doi:10.3389/fphar.2024.1442297)
Supplement: Supplementary file 4 [file Table9.docx]

Supplementary Material

# Supplementary Tables

**Supplementary Table 9 Traditional Chinese medicine prescriptions for the application of rhubarb and its processed products.**

| **Formula**  **effect** | **Formula name** | **Indications** | **Rhubarb used** | **Other components** | **Rhubarb effect** | **Dosage form** | **Source** |
| --- | --- | --- | --- | --- | --- | --- | --- |
| Purgation | Da Cheng Qi Tang | 1. Constipation, increased passing of flatus, abdominal distension, hard and pain, refusal to press, possible tidal fever, sweating one after another, obnubilation, talking nonsense, dry and yellow tongue coating, or burnt black and cracked, with deep and replete pulse;  2. The stool is cyan and watery, with a foul odor and pain around the navel, hard and lumpy when pressed, dry mouth and tongue, with slippery and replete pulse;  3. Cold hands and feet, stiff neck and back, convulsions in limbs, and even ankylosing spasms and mania, caused by excess internal heat | Raw rhubarb  (12g) | Magnoliae officinalis cortex (24g) Aurantii fructus immaturus (12g) Natrii sulfas (9g) | Purgation, clearing away pathogenic heat and stagnation in the intestines and stomach | Decoction | *Treatise on Cold Damage Diseases*  *(Shang han lun)* |
|  | Da Xian Xiong Tang | Pain and hard below the heart, refusal to press, or hard, full and pain from the bottom of the heart to the lower abdomen, inability to touch, constipation, fever from 3 to 5 pm in the afternoon, or shortness of breath and fidget, dry mouth and tongue, with deep, tense and strong pulse | Raw rhubarb  (10g) | Natrii sulfas (10g) 　 Kansui radix (1g) | clearing away pathogenic heat in the chest and abdomen | Decoction | *Treatise on Cold Damage Diseases*  *(Shang han lun)* |
|  | Da Huang Fu Zi Tang | Abdominal pain and constipation, pain in the lower part of one side of hypochondrium, fever, fear of cold, cold limbs, white and greasy tongue coating, with wiry tense pulse | Raw rhubarb  (9g) | Aconiti lateralis radix praeparata (12g) 　 Asari radix et rhizoma (3g) | Purgation, clearing away stagnation in the intestines and stomach | Decoction | *Synopsis of Golden Chamber*  *(Jin gui yao lve)* |
|  | Ma Zi Ren Wan  (also known as Pi Yue Wan) | Constipation, frequent urination, abdominal distension and pain, red tongue with yellow coating, rapid pulse | Raw rhubarb  (12g) | Cannabis fructus (20g)  Paeoniae radix alba (9g) 　 Aurantii fructus immaturus (9g)  Magnoliae officinalis cortex (9g) 　 Armeniacae semen amarum (10g) | Purgation, clearing away pathogenic heat and stagnation in the intestines and stomach | Pilula | *Treatise on Cold Damage Diseases*  *(Shang han lun)* |
|  | Zhou Che Wan | Edema, thirst, coarse breathing sound, hard abdomen, oliguria, constipation, with deep, rapid and strong pulse | Raw rhubarb  (60g) | Pharbitidis semen (120g)  Kansui radix (30g) 　 Genkwa flos (30g)  Euphorbiae pekinensis radix (30g) 　 Citri reticulatae pericarpium viride (15g)  Citri reticulatae pericarpium (15g)　 Aucklandiae radix (15g)  Arecae semen(15g) 　 Calomelas(3g) | Cleaning the gastrointestinal tract, eliminating edema, purgating heat | Pilula | *Taiping Holy Prescriptions for Universal Relief*  *(Taiping shenghui fang)* |
|  | Huang long Tang | The stool is cyan and watery, with a foul odor, or constipation, abdominal distension and pain, refusal to press, fever, thirst, tiredness, shortness of breath, obnubilation, talking nonsense, even hands unconsciously grasping in the air, coma, cold limbs, burnt yellow or black tongue coating, with feeble pulse | Raw rhubarb  (9g) | Natrii sulfas(6g)  Aurantii fructus immaturus (9g) Magnoliae officinalis cortex (9g)  Glycyrrhizae radix et rhizoma (3g) Ginseng radix et rhizoma (9g)  Angelicae sinensis radix (6g) Zingiberis rhizoma recens (9g)  Jujubae fructus (20g) Platycodonis radix (6g) | Purgation, clearing away pathogenic heat and stagnation in the intestines and stomach | Decoction | *Six Books on Cold Damage Diseases*  *(Shang han liu shu)* |
| Relieving exterior syndromes, purgation | Da Chai Hu Tang | Irregular alternating attacks of fever and aversion to cold, full and distress in chest and hypochondrium, continuous vomiting, depression and fidget, hard and full or sudden pain below the heart, constipation or diarrhea, yellow tongue coating, with wiry, rapid and strong pulse | Raw rhubarb  (6g) | Bupleuri radix (24g)  Scutellariae radix (9g) 　 Paeoniae radix alba (9g)  Pinelliae rhizoma(9g) 　 Aurantii fructus immaturus (9g)  Jujubae fructus (40g) 　 Zingiberis rhizoma recens (15g) | Clearing and purgating heat of Yangming's stagnation, unblocking the intestines | Decoction | *Synopsis of Golden Chamber*  *(Jin gui yao lve)* |
|  | Kai Jie Wan | After catching a cold: fever, headache, sore legs, chest distension, dry stool, more than ten days of abdominal distension tension, but unable to defecate, took sweating medicine but still not alleviated | Cooked rhubarb  (30g) | Pharbitidis semen (30g)  Pinelliae rhizoma (8g) Arisaematis rhizoma (4g)  Alumen (4g) Chinese honeylocust fruit  (30g)  Descurainiae semen/  Lepidii semen (8g) Aucklandiae radix (4g)  Citri reticulatae pericarpium viride (8g) Aurantii fructus immaturus (8g) | Clearing heat and unblocking the intestines | Pilula | *Compilation of good and effective prescriptions*  *(Ji yan liang fang)* |
| Activating blood circulation and removing blood stasis | Tao He Cheng Qi Tang | 1. Normal urination but feel tense in the lower abdomen, fever at night, fidget and hyperactivity, even obnubilation, talking nonsense;  2. Amenorrhea and dysmenorrhea caused by blood stasis, with deep, replete and unsmooth pulse | Raw rhubarb  (12g) | Persicae semen (12g) Cinnamomi ramulus (6g) 　 Glycyrrhizae radix et rhizoma (6g) Natrii sulfas (6g) | Clearing heat and promoting blood circulation, allowing blood stasis and pathogenic heat to be excreted through feces | Decoction | *Treatise on Cold Damage Diseases*  *(Shang han lun)* |
|  | Di Dang Tang | 1. Normal urination but feel hard and full in the lower abdomen, forgetfulness, manic hyperactivity, with black stools that are easily excreted;  2. Amenorrhea, hard and full in the lower abdomen, refusal to press | Raw rhubarb  (9g) | Hirudo (6g)　 Tabanus (6g) Persicae semen (5g) | Promoting stagnation of pathogenic heat to be excreted downward | Decoction | *Treatise on Cold Damage Diseases*  *(Shang han lun)* |
|  | Xia Yu Xue Tang | 1. Prickling pain with hard lumps in the lower abdomen after parturition, refusal to press, or lochia cannot be discharged smoothly, dry mouth and tongue, dry stool, and even the skin is rough and dry, brown and scaly. The tongue is purple red and has ecchymosis, yellow and dry coating, with deep, unsmooth and strong pulse;  2. Unsmooth menstruation caused by blood stasis | Wine rhubarb  (6g) | Persicae semen (12g) Eupolyphaga steleophaga (9g) | Activating blood circulation and removing blood stasis, allowing obstinate blood stasis to be excreted | Pilula | *Synopsis of Golden Chamber*  *(Jin gui yao lve)* |
|  | Dang Gui Jiao Ai Tang | Pregnant women’s Chong and Ren Vessels impairment due to excessive strain, affecting the fetus and experiencing symptoms such as lower back and abdominal pain and irregular vaginal bleeding | Cooked rhubarb  (8g) | Chuanxiong rhizoma (8g)  Angelicae sinensis radix (8g) Poria (8g)  Atractylodis macrocephalae rhizoma (8g) Ginseng radix et rhizoma (8g)  Eucommiae cortex (8g) Asini corii colla (8g)  Artemisiae argyi folium (8g) Furnace Soil (8g)  Glycyrrhizae radix et rhizoma (3g) | Eliminating blood stasis and normalizing the function of Chong and Ren Vessels | Decoction | *Poor Township Convenience Formulas*  *(Qiong xiang bian fang)* |
|  | Da Huang Zhe Chong Wan | Extremely weak caused by long-term overwork or bad living habits. Thin and weak body, swell and full abdomen, unable to eat or drink, the skin is rough and dry, brown and scaly, with dark eye circles and blurred vision | Cooked rhubarb  (7.5g) | Scutellariae radix (6g)  Glycyrrhizae radix et rhizoma (9g) Persicae semen (6g)  Armeniacae semen amarum (6g)  Paeoniae radix alba (12g)  Rehmanniae radix (30g) Toxicodendri resina (3g)  Tabanus (6g) 　 Hirudo (6g)  Northeast Giant Black Chafer (6g) Eupolyphaga steleophaga (3g) | Clearing away stagnation in the intestines and stomach, activating blood circulation and removing blood stasis | Pilula | *Synopsis of Golden Chamber*  *(Jin gui yao lve)* |
|  | Ji Ming San | Sharp-metal trauma, falls and injuries, blood stasis and accumulation, Extreme fidget | Cooked rhubarb  (30g) | Angelicae sinensis radix (18g) Persicae semen (9g) | Activating blood circulation and removing blood stasis | Powder | *Treatise on Three Categories of Pathogenic Factors*  *(Sanyin jiyi bingzheng fanglun)* |
| Hemostasis | Shi Hui San | Hematemesis, hemoptysis, epistaxis, etc., sudden and urgent massive bleeding with bright red in color, red tongue, rapid pulse | Rhubarb charcoal  (9g) | Cirsii japonici herba carbonisata (9g)　Cirsii herba charcoal (9g)　 Nelumbinis folium charcoal (9g)  Imperatae rhizoma charcoal (9g)  Platycladi cacumen charcoal (9g)　 Rubiae radix et rhizoma charcoal (9g)　 Gardeniae fructus charcoal (9g)　 Moutan cortex charcoal (9g)  Trachycarpi petiolus charcoal (9g) | Clearing heat and reducing fire, allowing pathogenic heat to be excreted through feces, astringing and hemostasis | Powder | *Miraculous Book of Ten Medicines*  *(Shi yao shen shu)* |
|  | Si Hong Dan | Hematemesis, epistaxis, hematochezia, hematuria and irregular vaginal bleeding caused by lung heat or sudden anger | Rhubarb charcoal  (30g) | Angelicae sinensis radix charcoal (30g) Typhae pollen charcoal (30g) Sophorae flos charcoal (30g) Asini corii colla (30g) | Cooling blood and hemostasis | Pilula | *Selected Formulas of Traditional Chinese Medicine in Beijing* |
| Clearing heat | Xie Xin Tang | 1. Hematemesis, epistaxis, etc. caused by excessive internal heat; 2. Jaundice, full chest, fidget and fever; 3. Red and swollen eyes and ulcerative stomatitis caused by upward rushes of stagnant heat;  4. Purulent infection on the body surface, fidget and fever in chest, dry stools, constipation, etc. | Raw rhubarb  (6g) | Coptidis rhizoma (3g) Scutellariae radix (3g) | Clearing heat, eliminating fullness, allowing pathogenic heat to be excreted through feces | Decoction | *Synopsis of Golden Chamber*  *(Jin gui yao lve)* |
|  | Liang Ge San | Fidget, thirst, red face, dry and burnt lips, fever in chest and diaphragm, ulcerative stomatitis, inability to fall asleep or even lie flat, obnubilation, talking nonsense, manic hyperactivity, or sore throat, hematemesis, epistaxis, poor bowel movements or constipation, deep urine color, red and yellow tongue coating, with slippery and rapid pulse | Raw rhubarb  (12g) | Natrii sulfas (12g)  Glycyrrhizae radix et rhizoma (12g) 　 Menthae haplocalycis herba (6g)  Scutellariae radix (6g) 　 Forsythiae fructus (25g)  Lophatheri herba (3g)  Gardeniae fructus (6g)  Mel (6g) | Promoting pathogenic heat to be excreted downward, purgation, relieving restlessness | Powder | *Prescriptions Collected by the Public Pharmacy*  *(Taiping huimin heji jufang)* |
|  | Xi Jiao Di Huang Wan | Coughing, hematemesis, epistaxis, fidget, strong heartbeat caused by heat stagnation in lung ang stomach or exuberant liver fire | Rhubarb charcoal  (30g) | Rehmanniae radix (8g)  Paeoniae radix alba (8g) Moutan cortex (15g)  Platycladi cacumen charcoal (15g) Nelumbinis folium charcoal (30g) Imperatae rhizoma (15g) Gardeniae fructus charcoal (30g) | Promoting pathogenic heat to be excreted downward, cooling blood and hemostasis | Pilula | *Selected Formulas of Traditional Chinese Medicine in Beijing* |
|  | Gu Chi Mi Fang | Odontalgia caused by stomach fire | Raw rhubarb  (30g) Cooked rhubarb  (30g) | Gypsum fibrosum (60g)  Eucommiae cortex (30g) Halitum (60g) Alumen (30g) Angelicae sinensis radix (15g)  Drynariae rhizoma (30g) | Clearing heat, activating blood circulation | Powder | *Medical Prescriptions Written for Empress Dowager Cixi and Emperor Guangxu with Commentary*  *(Cixi guangxu yifang xuanyi)* |
|  | Jia Jian Xie Xin San | Localized nodules protruded outward on the bulbar conjunctiva and sclera, due to fire stagnation in heart and affecting the lung: initially, they are granular protrusions, circular or elliptical in shape, dark red or purplish red in color, with tenderness. Later, the granules gradually become larger, red and painful, with photophobia, tearing and unclear vision | Wine rhubarb  (6g) | Coptidis rhizoma (3g)  Gardeniae fructus (6g) Scutellariae radix (9g)  Platycodonis radix (6g) Anemarrhenae rhizoma (9g)  Scrophulariae radix (6g) Paeoniae radix rubra (9g)  Angelicae sinensis radix (6g) Schizonepetae herba (1.5g) | Purgation, cleaning the large intestine to relieve lung heat | Powder | *Zhang Jiechun's Ophthalmology Diagnosis and Treatments*  *(Zhang jiechun yanke zhengzhi)* |
|  | Xie Fei Qing Gan Tang | The bulbar conjunctiva and sclera are red and swollen, and suddenly appear gray and white cloudy membrane covering the cornea from around the cornea, which is high around the circumference and low in the middle, shaped like petals. Mainly due to lung fire affecting the liver | Wine rhubarb  (6g) | Lonicerae japonicae flos (18g)  Scutellariae radix (12g) Bupleuri radix (6g)  Celosiae semen (3g) Paeoniae radix rubra (9g)  Moutan cortex (9g) Indigo naturalis (0.3g) | Clearing and purgating excess heat of lung | Decoction | *Zhang Jiechun's Ophthalmology Diagnosis and Treatments*  *(Zhang jiechun yanke zhengzhi)* |
|  | Jiu Ji Jie Du Wan | 1. Epidemic diseases, sore throat, jugular vein distension, stiff tongue, hoarseness, stuffy nose, difficult to drink;  2. Edema of the head and face, hard mass nodules, wet scabies eczema, discharge of pus in the ears, redness and swelling of the eyelid, ulcerative stomatitis | Wine rhubarb  (15g) | Glycyrrhizae radix et rhizoma (30g)  Platycodonis radix (30g) Schizonepetae herba (15g)  Saposhnikoviae radix (15g) Forsythiae fructus (15g)  Scutellariae radix (15g) Coptidis rhizoma (15g)  Menthae haplocalycis herba (15g) Cimicifugae rhizoma (15g)  Bombyx batryticatus (8g) Typhae pollen (8g)  Indigo naturalis (8g) Natrii sulfas (8g)  Belamcandae rhizoma (8g) | Purgation, promoting pathogenic heat and blood stasis to be excreted downward, eliminating lumps | Pilula | *Complete Book of Summer-Heat Damage*  *(Shang shu quan shu)* |
|  | Shu Du Yin | In the early stage of epidemic diphtheria, after taking BaiduSan, the white pseudomembrane does not disappear but increases instead, oliguria, with dark yellow color, or constipation, or the stool is watery and black | Cooked rhubarb  (30g) | Rhinoceros horn (15g)  Scutellariae radix (10g) Indigo naturalis (20g)  Bombyx batryticatus (15g) Anemarrhenae rhizoma (10g)  Forsythiae fructus (10g) Plantaginis semen (10g)  Tetrapanacis medulla (10g) Cicadae periostracum (15g)  Gardeniae fructus (10g) Arctii fructus (10g) | Reducing fire, detoxification | Decoction | *Contribution to laryngology*  *(Hou ke zhong fu)* |
|  | Shao Yao Tang | Abdominal pain, stool with pus and blood, anus burning with a feeling of falling and swelling, urgent defecation sense but difficult to defecate, oliguria, with dark yellow color, yellow greasy tongue coating, with wiry and rapid pulse | Raw rhubarb  (6g) | Paeoniae radix alba (30g)  Angelicae sinensis radix (15g)　 Coptidis rhizoma (15g)  Arecae semen (6g)　 Aucklandiae radix (6g)  Glycyrrhizae radix et rhizoma (6g)  Scutellariae radix (9g)  Cinnamomi cortex (5g) | Clearing heat and drying dampness, promoting circulation of qi and blood, purgation, promoting pathogenic heat and dampness to be excreted downward | Decoction | *Compilation of Pathogenesis, Health Care, and Lifesaving Methods in Su Wen*  *(Suwen bingji qiyi bao ming ji)* |
|  | Qing Jin Yang Ying Tang | Hemorrhoids, anal fistula, anal pain with a feeling of falling and swelling, constipation, urgent defecation sense but difficult to defecate | Cooked rhubarb  (15g) | Angelicae sinensis radix (20g)  Paeoniae radix alba (8g) Rehmanniae radix (20g)  Coptidis rhizoma (20g) Forsythiae fructus (10g)  Scutellariae radix (15g) Aurantii fructus (20g)  Cannabis fructus (20g) Poria (10g)  Glycyrrhizae radix et rhizoma (5g) Trichosanthis radix (10g) | Clearing heat and drying dampness, promoting circulation of qi and blood, purgation | Decoction | *Compendium of Surgery*  *(Wai ke da cheng)* |
| Detoxification | Da Huang Mu Dan Tang | In the early stage of appendicitis, pain in right lower abdomen, refusal to press, or right leg cannot be straightened, stretch will increase the pain, and even local swelling, or intermittent fever, sweating, aversion to cold, thin greasy and yellow tongue coating, with slippery and rapid pulse | Raw rhubarb  (12g) | Moutan cortex (3g) 　 Persicae semen (9g) Chinese waxgourd semen (30g) Natrii sulfas (6g) | Clearing stagnation of dampness, heat and blood stasis in the intestines | Decoction | *Synopsis of Golden Chamber*  *(Jin gui yao lve)* |
|  | Jia Zi Hua Du Wan | Purulent infection on the body surface, syphilis | Cooked rhubarb  (25g) | Cimicifugae rhizoma (20g)  Bovis calculus (4g) Milk (10g)  Sulfur (10g) Cinnabaris (17g)  Olibanom (17g) Rosae chinensis flos (15g) Bombyx batryticatus (15g) Malayan pangolin (15g) Dictamni cortex (15g) Aucklandiae radix (25g) Moutan cortex (25g) | Reducing fire, detoxification | Pilula | *Complete book of experience in sores and ulcers*  *(Chuangyang jingyan quan shu)* |
|  | Jia Wei Hua Du Dan | In the early stage, there are often ringworm spots on the groin and scrotum, with severe itching and a purple black color resembling cowhide, gradually spreading to the lower abdomen, buttocks and thighs, and even to the chest, back, and hypochondrium in the later stage. The appearance looks like armor, accompanied by dark yellow urine color, dry and black stool, with a feeling of something rushing up in the body, headache, irritability | Cooked rhubarb  (10g) | Bovis calculus (4g)  Amber (5g) Draconis sanguis (15g)  Sulfur (15g) Cinnabaris (15g)  Tiger tibia (15g) Rhinoceros horn (15g)  Malayan pangolin (15g) Zaocys (15g)  Stalactitum (20g) Borneolum (3g)  Moschus (2g) Calomelas (5g) | Clearing excess heat, reducing fire, detoxification | Pilula | *A Secret Mirror on the Treatment of Syphilis*  *(Meichuang zhengzhi mijian)* |
| Eliminating food accumulation and stagnation | Zhi Shi Dao Zhi Wan | Abdominal distension pain, constipation or diarrhea, oliguria, with dark yellow color, yellow greasy tongue coating, deep strong pulse | Raw rhubarb  (30g) | Aurantii fructus immaturus (15g)  Medicated leaven (15g) Poria (9g)  Scutellariae radix (9g) 　 Coptidis rhizoma (9g)  Atractylodis macrocephalae rhizoma (9g) 　 Alismatis rhizoma (6g) | Promoting stagnation of dampness and heat to be excreted downward | Pilula | *Differentiation on Endogenous and Exogenous Diseases*  *(Nei wai shang bian huo lun)* |
|  | Mu Xiang Bing Lang Wan | Abdominal distension and pain, or diarrhea, feces with pus and blood, urgent defecation sense but difficult to defecate, or constipation, yellow greasy tongue coating, with deep and replete pulse | Raw rhubarb  (9g) | Aucklandiae radix (3g)  Arecae semen (3g)　 Citri reticulatae pericarpium viride (3g)  Citri reticulatae pericarpium (3g)　 Curcumae rhizoma (3g)  Coptidis rhizoma (3g) 　 Phellodendri chinensis cortex (9g)  Cyperi rhizoma (12g) 　 Pharbitidis semen (12g) | Clearing heat, purgation, eliminating stagnation, guiding the downward movement of pathogenic qi | Pilula | *Confucians' Duties to Their Parents*  *(Ru men shi qin)* |
|  | Kuan Zhong Jiang Ni Tang | Abdominal distension, hiccup and belching, poor appetite, caused by gastrointestinal food accumulation | Wine rhubarb  (6g) | Raphani semen (10g)  Crataegi fructus (10g)  Hordei fructus germinatus (10g)  Medicated leaven (10g)  Magnoliae officinalis cortex (6g)  Aurantii fructus immaturus (6g) | Purgation, clearing away pathogenic heat and stagnation in the intestines and stomach | Decoction | *A Humble Opinion on Warm Diseases*  *(Wen bing zou yan)* |
| Regulating qi | Liu Mo Yin Zi | Hypochondrium and abdominal distension, or abdominal pain, constipation, decreased appetite, thin and greasy tongue coating, with wiry pulse | Raw rhubarb  (6g) | Arecae semen (6g)　 Aquilariae lignum resinatum (6g)　 Aucklandiae radix (6g)　 Linderae radix (6g)　 Aurantii fructus (6g) | Purgation, eliminating stagnation, guiding the downward movement of pathogenic qi | Paste | *Effective Formulae Handed Down for Generations*  *(Shi yi de xiao fang)* |
| Clearing dampness | Yin Chen Hao Tang | Jaundice: the whole body, face and eyes are bright yellow, fever, no sweating or only sweating in the head, thirst and desire to drink, nausea and vomiting, mild abdominal fullness, dark yellow urine, poor bowel movement or constipation, red tongue and yellow greasy tongue coating, with deep rapid pulse or slippery strong pulse | Raw rhubarb  (6g) | Artemisiae scopariae herba (18g) 　 Gardeniae fructus (12g) | Reducing fire, purgation, allowing stagnation of dampness, heat and blood stasis to be excreted through feces | Decoction | *Treatise on Cold Damage Diseases*  *(Shang han lun)* |
|  | Ba Zheng San | Frequent micturition, urgent micturition, painful micturition, dark yellow turbid urine, or even anuria, lower abdominal tension, fullness, dry mouth and tongue, yellow greasy tongue coating, with slippery rapid pulse | Raw rhubarb  (9g) | Plantaginis semen (9g)  Dianthi herba (9g)　 Polygoni avicularis herba (9g)  Talcum (9g)　 Gardeniae fructus (9g)  Glycyrrhizae radix et rhizoma (9g) 　 Akebiae caulis (9g) | Clearing heat, purgation, letting the dampness and heat discharge separately with urine and feces | Powder | *Prescriptions Collected by the Public Pharmacy*  *(Taiping huimin heji jufang)* |
| Eliminating phlegm | Meng Shi Gun Tan Wan | Mental disorders, coma, or susceptibility to shock, palpitations, or coughing and wheezing with thick sputum, distension in chest and epigastrium, or dizziness and tinnitus, or multiple subcutaneous nodules in the neck, or involuntary movements of facial muscles, or insomnia, or frequent dreaming of strange objects, or sudden and inexplicable pain in the bones and joints, or respiratory and eating obstruction, fidget, constipation, yellow and greasy tongue coating, with slippery, rapid and strong pulse | Cooked rhubarb  (24g) | Scutellariae radix (24g) 　 Chloriti lapis (3g) 　 Aquilariae lignum resinatum (2g) | Clearing excess heat, opening a way for phlegm and fire to be excreted downward | Pilula | *Taiding Health Preservation Theory*  *(Tai ding yang sheng zhu lun)* |
|  | Niu Huang Huo Tan Wan | 1. Sudden fainting, loss of consciousness, mental disorder, palpitations, epilepsy, body stiffness, trismus, with a lot of sputum and saliva flowing out; 2. All asthma and chest tightness with obvious phlegm syndrome | Cooked rhubarb  (30g) | Arisaema cum bile (30g)  Scutellariae radix (30g) Bambusae concretio silicea (30g)  Fritillariae cirrhosae bulbus (18g) Natrii sulfas exsiccatus (18g)  Pharbitidis semen (18g) Typhonii rhizoma (18g)  Gastrodiae rhizoma (18g) Sulfur (4.5g)  Cinnabaris (4.5g) Chloriti lapis (4.5g)  Aquilariae lignum resinatum (6g) Bovis calculus (3g)  Moschus (2g) Borneolum syntheticum (1g) | Unblocking blood vessels, opening a way for phlegm, blood stasis and fire to be excreted downward | Pilula | *Concise Medical Encyclopedia*  *(Jian ming yi gou)* |
