# Supplementary material for: The application of rhubarb concoctions in traditional Chinese medicine and its compounds, processing methods, pharmacology, toxicology and clinical research
Source: Front Pharmacol. 2024 Aug 7;15:1442297. doi: 10.3389/fphar.2024.1442297 (PMC11335691; doi:10.3389/fphar.2024.1442297)
Supplement: Supplementary file 5 [file Table2.docx]

Supplementary Material

# Supplementary Tables

**Supplementary Table 2 Literature search strategy of components related to wine rhubarb, cooked rhubarb and rhubarb charcoal before and after processing.**

| **Database** | **Search strategy** |
| --- | --- |
| Pubmed | ((((((((((Rheum tanguticum[MeSH Terms]) OR (Rheum palmatum[MeSH Terms])) OR (Rheum[MeSH Terms])) OR (Rheum officinale[MeSH Terms])) OR (Da Huang[MeSH Terms])) OR (Huang, Da[MeSH Terms])) OR (Chinese Rhubarb[MeSH Terms])) OR (Rhubarb, Chinese[MeSH Terms])) OR (Rhubarb[MeSH Terms])) AND ((((((paozhi) OR (prepar*)) OR (processing)) OR (chinese medicine processing)) OR (processing chinese materia medica)) OR (tcm processing))) AND ((((((component) OR (constituent)) OR (composition)) OR (chemical component)) OR (chemical constituent)) OR (chemical composition)) Filters: English, from 2010/1 - 2024/4  112 results |
| Embase | #1 'rheum'/exp/mj OR 'rheum tanguticum'/exp/mj OR 'rheum officinale'/exp/mj OR 'rheum palmatum'/exp/mj OR 'da huang'/exp/mj OR 'huang, da'/exp/mj OR 'chinese rhubarb'/exp/mj OR 'rhubarb, chinese'/exp/mj OR 'rhubarb'/exp/mj  #2 paozhi OR 'prepar*' OR processing OR 'chinese medicine processing' OR 'processing chinese materia medica' OR 'tcm processing'  #3 component OR constituent OR composition OR 'chemical component' OR 'chemical constituent' OR 'chemical composition'  #1 AND #2 AND #3 AND [english]/lim AND [2010-2024]/py  11 results |
| Web of science | #1 ((((((((TS=(Rheum)) OR TS=(Rheum tanguticum)) OR TS=(Rheum palmatum)) OR TS=(Rheum officinale)) OR TS=(Da Huang)) OR TS=(Huang, Da)) OR TS=(Chinese Rhubarb)) OR TS=(Rhubarb, Chinese)) OR TS=(Rhubarb)  #2 (((((ALL=(paozhi)) OR ALL=(prepar*)) OR ALL=(processing)) OR ALL=(chinese medicine processing)) OR ALL=(processing chinese materia medica)) OR ALL=(tcm processing)  #3 (((((ALL=(component)) OR ALL=(constituent)) OR ALL=(composition)) OR ALL=(chemical component)) OR ALL=(chemical constituent)) OR ALL=(chemical composition)  #1 AND #2 AND #3 and English (Languages) and Publication Date (2010/1/1 - 2024/4/1)  200 results |
| CNKI | ((rheum OR raw rhubarb OR wine rhubarb OR cooked rhubarb OR rhubarb charcoal)(Theme) AND processing(Theme) AND component(Theme)) Filters: Chinese, from 2010/1/1 - 2024/4/1, Academic journals or Thesis  323 results |
