# Supplementary material for: The application of rhubarb concoctions in traditional Chinese medicine and its compounds, processing methods, pharmacology, toxicology and clinical research
Source: Front Pharmacol. 2024 Aug 7;15:1442297. doi: 10.3389/fphar.2024.1442297 (PMC11335691; doi:10.3389/fphar.2024.1442297)
Supplement: Supplementary file 6 [file Table5.docx]

Supplementary Material

# Supplementary Tables

**Supplementary Table 5 The compound changes of rhubarb charcoal before and after processing.**

| **References** | **Processing methods** | **Solvent used for sample preparation** | **Identification methods** | **Differences in compounds compared to raw rhubarb (increased)** | **Differences in compounds compared to raw rhubarb (decreased)** |
| --- | --- | --- | --- | --- | --- |
| Sun et al., 2024 | The raw rhubarb decoction pieces are roasted in a medicine stir-frying machine at 180℃ for 50 min, then removed and let dry | Water; methanol | Ultra-high performance liquid chromatography-quadrupole-electrostatic field orbitrap high-resolution mass spectrometry | Rhein; emodin; aloe-emodin; chrysophanol; physcion | Rhein-8-O-β-D-glucopyranoside; aloe-emodin 8-O-β-D-glucopyranoside; emodin 8-O-β-D-glucopyranoside; chrysophanol 8-O-β-D-glucopyranoside |
|  |  |  |  | Gallic acid | (-)-epicatechin 3-O-gallate; resveratrol 4'-O-β-D-(6''-O-galloyl)-glucopyranoside; (-)-epicatechin; (+)-catechin |
|  |  |  |  | 5-HMF | Lindleyin |
| Yang et al., 2023 | Oven heating | 70% methanol | High performance liquid chromatography | Free anthraquinones | Combined anthraquinones |
|  |  |  |  | Gallic acid | Sennoside |
|  |  |  |  | 5-HMF |  |
| Nan et al., 2019 | Stir-fry raw rhubarb at 200℃ for 15 minutes, until the surface is burnt black, the inside is burnt brown, spray a little water, extinguish the spark, take out and dry | Anthraquinone: methanol  Gallic acid: ultrapure water | High performance liquid chromatography | Rhein; emodin; aloe-emodin; chrysophanol | — |
|  |  |  |  | Gallic acid |  |
| Cai et al., 2018 | Not specifically mentioned | 50% methanol | High performance liquid chromatography | 5-HMF | — |
| Li et al., 2010 | According to the processing methods specified in the *Chinese Pharmacopoeia(2005 edition)* | 50% methanol | High performance liquid chromatography | 4'-hydroxyphenyl-2-butanone | 4'-hydroxyphenyl-2-butanone-4'-O-β-D-(6"-galloyl)-glucoside |
| Wang et al., 2010 | According to the processing methods specified in the *Chinese Pharmacopoeia* and the *National Standards for the Processing of Traditional Chinese Medicine*, prepared by Beijing Renwei Herbal Pieces Factory | 100% methanol | High performance liquid chromatography | Gallic acid | — |
| Tian et al., 2010 | According to the processing methods specified in the *Chinese Pharmacopoeia* and the *National Standards for the Processing of Traditional Chinese Medicine*, prepared by Beijing Renwei Decoction Pieces Factory | Methanol | High performance liquid chromatography | — | Aloe-emodin-3-CH2-O-β-D-glucoside |
| Li et al., 2011 | According to the processing methods specified in the *Beijing Standards for the Processing of Traditional Chinese Medicine decoction pieces (2006 edition)*, prepared by Beijing Qiancao Decoction Pieces Co., Ltd | Tannins: water  Anthraquinone: methanol; 8% hydrochloric acid; chloroform | Anthraquinone components by ultra-high performance liquid chromatography, and tannin components by colorimetry | Chrysophanol | Rhein; emodin; aloe-emodin; physcion |
|  |  |  |  |  | Total free anthraquinones; Total combined anthraquinones |
|  |  |  |  |  | Total tannins |
| Li, 2011 | According to the processing methods specified in the *Chinese Pharmacopoeia*, prepared by Beijing Renwei Herbal Pieces Factory | Methanol | High performance liquid chromatography | Rhein; emodin; aloe-emodin; chrysophanol; physcion | Emodin-8-O-β-D-glucopyranoside; aloe-emodin-8-O-β-D-glucoside; rhein-8-O-β-D-glucoside; aloe-emodin-3-CH2-O-β-D-glucoside |
|  |  |  |  | Gallic acid | (+)-catechin |
|  |  |  |  | 4'-hydroxyphenyl-2-butanone | Trans-3,5,4'-trihydroxystilbene-4'-O-β-D-(6"-O-galloyl)-glucoside; trans-3,5,4'-trihydroxystilbene-4'-O-β-D-glucoside |
|  |  |  |  |  | 4'-hydroxyphenyl-2-butanone-4'-O-β-D-(6"-galloyl)-glucoside; 4'-hydroxyphenyl-2-butane-4'-O-β-D-(6"-O-cinnamoyl)-glucoside; 4'-hydroxyphenyl-2-butane-4'-O-β-D-(2"-O-Galloyl-6"-O-(4'-hydroxy)-cinnamoyl)-glucoside |
| Yan et al., 2016 | According to the processing method of rhubarb in the *Science of Chinese Medicine Processing* | Water; methanol | High performance liquid chromatography | Gallic acid | Emodin; chrysophanol; physcion |
|  |  |  |  |  | Chrysophanol-1-O-glucoside |
| Yang et al., 2013 | Put rhubarb slices in a pot and heat them over high heat. Stir fry until the surface is burnt brown and the inside is dark brown. Sprinkle a little water to extinguish any sparks, then remove and cool thoroughly | Methanol; 10% hydrochloric acid; chloroform | High performance liquid chromatography | Emodin; chrysophanol; physcion | Rhein; aloe-emodin |
| Yang et al., 2020 | Rhubarb is laid flat in stainless steel discs to evenly heat them, and then baked in a laboratory oven at a temperature of 180, 200, 220℃ for 10-60 minutes respectively | 70% methanol | High performance liquid chromatography | Rhein; emodin; aloe-emodin; chrysophanol; physcion  (Increased first and then decreased) | Emodin-8-O-β-D-glucoside; chrysophanol-8-O-β-D-glucoside; aloe-emodin-8-O-β-D-glucoside; physcion-8-O-β-D-glucoside; rhein-8-O-β-D-glucoside |
|  |  |  |  | Gallic acid  (Increased first and then decreased) |  |
|  |  |  |  | 5-HMF  (Increased first and then decreased) | Sennoside A; sennoside B |
| Zeng et al., 2020 | Heat the clean raw rhubarb slices over low heat first, then turn to high heat, stir fry until the surface is burnt black, the section is burnt brown, there is thick smoke coming out and sparks can be seen. When there is a burnt aroma, spray clean water, extinguish the sparks, take them out and let them cool | 0.1% sodium bicarbonate solution | Reversed phase high performance liquid chromatography | — | Sennoside A; sennoside B |
| Han, 2021 | Clean rhubarb slices are stir fried over high heat at (200 ± 20) ℃ until the surface is burnt black and the interior is burnt brown | 8 times purified water | High performance liquid chromatography | Chrysophanol; physcion | Rhein; aloe-emodin |
| Zhang et al., 2022 | Place the rhubarb pieces in a pot, fry over high heat until the inside color is dark brown, the surface color is burnt brown, spray with a little water to ensure that there is no spark, and cool completely | 70% methanol | High performance liquid chromatography | Gallic acid; catechin | Rhein; emodin; aloe-emodin; chrysophanol; physcion |
|  |  |  |  |  | Emodin-8-O-β-D-glucopyranoside; chrysophanol-8-O-β-D-glucoside; aloe-emodin-8-O-β-D-glucoside; physcion-8-O-β-D-glucoside; rhein-8-O-β-D-glucoside |
|  |  |  |  | 4-(4'hydroxyphenyl)-2-butanone | Sennoside A; sennoside B |
|  |  |  |  |  | Polydatin |
| Wang et al., 2014 | Stir fry rhubarb until it appears brownish black on the outside and a burnt brownish black inside | Methanol | Ultra-performance liquid chromatography-quadrupole\time-of- flight mass spectrometry | Chrysophanol dimethyl ether | Emodin-8-O-glucoside; emodin-O-glucoside |
|  |  |  |  | Gallic acid-3-O-glucoside | Catechin-glucopyranoside; cinnamyl-galloyl-glucoside derivative |
|  |  |  |  | Torachrysone |  |
| Wang et al., 2015a | Directly purchased rhubarb charcoal after processing | 80% methanol; magnolol solution (IS, 200 μg/mL) | Ultra fast liquid chromatography with ion trap/time-of-flight mass spectrometry | Physcion | 6-dehydroxylaccaic acid D; rhein; emodin |
|  |  |  |  |  | Chrysophanol-O-glucoside; acetyl-chrysophanol-O-glucoside; 6-dehydroxylaccaic acid D-glucoside; emodin-O-glucoside; acetyl-emodin-O-glucoside; emodin-O-(-6’-O-malonyl)-glucoside |
|  |  |  |  |  | (+)-catechin; (epi)catechin-O-gallate; gallic acid; cinnamoyl-O -glucose-O-galloyl; cinnamoyl-O-glucose-O-digalloyl |
|  |  |  |  |  | 6-hydroxyrumicin-8-O-glucoside; torachrysone-8-O-glucoside |
| Wang et al., 2015b | Directly purchased rhubarb charcoal after processing | 80% methanol; naringenin solution (IS, 4 mg/mL) | High performance liquid chromatography coupled with tandem mass spectrometry | — | Rhein; emodin; aloe-emodin; chrysophanol; physcion |
|  |  |  |  |  | Rhein-8-O-b-D-glucoside; emodin-1-O-b-D-glucoside; emodin-8-O-b-D-glucoside; aloe-emodin-8-O-b-D-glucoside; chryphanol-8-O-b-D-glucoside |
|  |  |  |  |  | Sennoside A; sennoside B |

**References**

Cai, X. J., Xu, H. X., Lin, S. H., Yu, N. C. (2018). Separation and identification of the new chemical constituent from rhubarb charcoal and its formational rule. *Chinese Journal of Hospital Pharmacy*, 38, 2336-2339. doi:10.13286/j.cnki.chinhosppharmacyj.2018.22.09.

Han, H. F. (2021). Correlation Between Anthraquinone and Antibacterial Activity in Different Processed Products of Dahuang (Rhubarb) by Multiple Regression Analysis. *Guiding Journal of Traditional Chinese Medicine and Pharmacy*, 27, 65-68. doi:10.13862/j.cnki.cn43-1446/r.2021.01.014.

Li, H. F., Sun, Q., Wang, J. B., Jin, C., Xiao, X. H. (2011). Analysis on Change Law of Main Chemical Constituents of Rhubarb After Processing. *Journal of Shanxi University of Chinese Medicine*, 12, 14-17.

Li, L. (2011). *Study on the Variation Rules of Material Basis of Rhubarb after Processing*. doctor's thesis, China Academy of Chinese Medical Sciences.

Li, L., Zhang, C., Xiao, Y. Q., Chen, D. D., Tian, G. F., Wang, Y. (2010). Comparison of two butyrophenone constituents in 5 kinds of pieces of Dahuang (Radix et Rhizoma Rhei). *Journal of Beijing University of Traditional Chinese Medicine*, 33, 559-561.

Nan, J. H., Zhang, X. S., Hua, Y. L., Wei, Y. M. (2019). Study on Variations of Five Chemical Compositions in Raw Rhubarb and Charred Rhubarb. *Progress in Veterinary Medicine*, 40, 58-63. doi:10.16437/j.cnki.1007-5038.2019.04.011.

Sun, J., Xu, W. J., Zhong, L. Y., Chen, J. B., DONG, L. (2024). UPLC-QE-Orbitrap-MS combined with network pharmacology to explore differential components and mechanisms of raw and scorched rhubarb for treatment of ulcerative colitis. *China Journal of Chinese Materia Medica*, 49, 1834-1847. doi:10.19540/j.cnki.cjcmm.20231226.302.

Tian, G. F., Zhang, C., Li, L., Xiao, Y. Q., Chen, D. D., Wang, Y. (2010). Variety regulation of aloe-emodin-3-CH2-O-β-D-glucopyranoside and emodin-8-O-β-D-glucopyranoside in five processed pieces from Rheum palmatum. *China Journal of Chinese Materia Medica*, 35, 2437-2439.

Wang, M., Fu, J. F., Guo, H. M., Tian, Y., Xu, F. G., Song, R., et al. (2015a). Discrimination of crude and processed rhubarb products using a chemometric approach based on ultra fast liquid chromatography with ion trap/time-of-flight mass spectrometry. *J Sep Sci*, 38, 395-401. doi: 10.1002/jssc.201401044. Epub 2015 Jan 7. PMID: 25421806.

Wang, M., Tian, Y., Lv, M. Y., Xu, F. G., Zhang, Z. J., Song, R. (2015b). Targeted quantitative analysis of anthraquinone derivatives by high-performance liquid chromatography coupled with tandem mass spectrometry to discriminate between crude and processed rhubarb samples. *Analytical Methods*, 7, 5375-5380. doi: 10.1039/c5ay01067e.

Wang, Y., Li, L., Zhang, C., Xiao, Y. Q., Chen, D. D., Tian, G. F. (2010). Comparison of gallic acid and catechin contents in five processed products of Rheum palametum. *China Journal of Chinese Materia Medica*, 35, 2267-2269.

Wang, Z. H., Wang, D. M., Zheng, S. H., Wu, L. B., Huang, L. F., Chen, S. L. (2014). Ultra-performance liquid chromatography-quadrupole\time-of- flight mass spectrometry with multivariate statistical analysis for exploring potential chemical markers to distinguish between raw and processed Rheum palmatum. *BMC Complement Altern Med*, 14, 302. doi: 10.1186/1472-6882-14-302. PMID: 25128184; PMCID: PMC4147172.

Yan, Y. G., Yin, L. M., Wang, H. Y., Guo, L. L., Deng, C. (2016). Simultaneous Determination of 10 Kinds of Chemical Components in Processed Products of Rhei Radix et Rhizoma. *China Pharmacy*, 27, 3839-3842.

Yang, L., Wen, Y. X., Liu, Y., Cheng, Y. R., Shi, X. J., Gong, Y. T., et al. (2020). Study on relationship between color characteristics of rhubarb charcoal in heating process and contents of 14 chemical components. *China Journal of Chinese Materia Medica*, 45, 4230-4237. doi:10.19540/j.cnki.cjcmm.20200622.307.

Yang, L., Yang, D. P., Sun, J., Dong, L.Chen, J. B. (2023). Study on the Variation Patterns of the Potential Q-Markers for the Efficacy Enhancement and Toxicity Attenuation during the Scorching Process of Rhubarb Charcoal. *Guiding Journal of Traditional Chinese Medicine and Pharmacy*, 29, 66-73. doi:10.13862/j.cn43-1446/r.2023.11.012.

Yang, M., Xu, B. H., Wang, D. G., Chen, G. T. (2013). Effect of different processing methods on the content of five anthraquinones in Rhei Radix et Rhizoma. *Journal of Nantong University (Medical Sciences)*, 33, 385-387.

Zeng, C., Lu, M. Y., Mo, T. T., Qin, Y. S., Huang, M. (2020). Processing of Dahuang(Rhubarb) and Establishment of Determination Methods of Sennanoside A and Sennanoside B in Different Processed Products. *Chinese Archives of Traditional Chinese Medicine*, 38, 47-52+263. doi:10.13193/j.issn.1673-7717.2020.11.013.

Zhang, Q., Chen, Y. Y., Yue, S. J., Wang, W. X., Zhao, C. B., Song, Y. J., et al. (2022). Study on the content changes of 16 chemical components in Radix et Rhizoma Rhei and its different processed products. *Chin J Tradit Chin Med Pharm*, 37, 1036-1040.
