# Supplementary material for: The application of rhubarb concoctions in traditional Chinese medicine and its compounds, processing methods, pharmacology, toxicology and clinical research
Source: Front Pharmacol. 2024 Aug 7;15:1442297. doi: 10.3389/fphar.2024.1442297 (PMC11335691; doi:10.3389/fphar.2024.1442297)
Supplement: Supplementary file 7 [file Table3.docx]

Supplementary Material

# Supplementary Tables

**Supplementary Table 3 The compound changes of wine rhubarb before and after processing.**

| **References** | **Processing methods** | **Solvent used for sample preparation** | **Identification methods** | **Differences in compounds compared to raw rhubarb (increased)** | **Differences in compounds compared to raw rhubarb (decreased)** |
| --- | --- | --- | --- | --- | --- |
| Li et al., 2010 | According to the processing methods specified in the *Chinese Pharmacopoeia(2005 edition)* | 50% methanol | High performance liquid chromatography | 4'-hydroxyphenyl-2-butanone-4'-O-β-D-(6″-galloyl)-glucoside | 4'-hydroxyphenyl-2-butanone |
| Wang et al., 2010 | According to the processing methods specified in the *Chinese Pharmacopoeia* and the *National Standards for the Processing of Traditional Chinese Medicine*, prepared by Beijing Renwei Herbal Pieces Factory | 100% methanol | High performance liquid chromatography | Gallic acid; catechin | — |
| Tian et al., 2010 | According to the processing methods specified in the *Chinese Pharmacopoeia* and the *National Standards for the Processing of Traditional Chinese Medicine*, prepared by Beijing Renwei Decoction Pieces Factory | Methanol | High performance liquid chromatography | Emodin-8-O-β-D-glucoside | Aloe-emodin-3-CH2-O-β-D-glucoside |
| Li et al., 2011 | According to the processing methods specified in the *Beijing Standards for the Processing of Traditional Chinese Medicine decoction pieces(2006 edition)*, prepared by Beijing Qiancao Decoction Pieces Co., Ltd | Tannins: water  Anthraquinone: methanol; 8% hydrochloric acid; chloroform | Anthraquinone components by ultra-high performance liquid chromatography, and tannin components by colorimetry | Emodin; aloe-emodin; chrysophanol; total free anthraquinone | Physcion; total combined anthraquinone; total tannins |
| Yang et al., 2013 | Mix rhubarb slices with yellow wine, moisten until fully soaked, place in a pot, stir fry over low heat until dry, take out and let cool. Processed in a ratio of rhubarb to yellow wine at 10:1 | Methanol; 10% hydrochloric acid; chloroform | High performance liquid chromatography | Emodin; aloe-emodin | Rhein; chrysophanol; physcion |
| Yang et al., 2012 | Clean 50g rhubarb slices or pieces, spray with 5g yellow wine, moisten for 30min, after the wine is sucked up, put into a hot pot, stir-fry gently for 15min, until the surface is dark brown or brown, the moisture has dried, occasionally burnt spots, solid texture, a little wine aroma, then take out to cool, remove debris | 70% methanol | High performance liquid chromatography | Rhein; emodin; aloe-emodin; chrysophanol; physcion | Sennoside A; sennoside B |
|  |  |  |  | Gallic acid |  |
| Yan et al., 2016 | According to the processing method of rhubarb in the *Science of Chinese Medicine Processing* | Water; methanol | High performance liquid chromatography | — | Rhein; emodin; aloe-emodin; chrysophanol; physcion |
|  |  |  |  |  | Chrysophanol-1-O-glucoside; emodin-8-O-glucoside |
|  |  |  |  |  | Sennoside B |
|  |  |  |  |  | Gallic acid; catechin |
| Zeng et al., 2020 | Wash the raw rhubarb pieces and place them in a container, add a quantity of yellow wine and mix well until the yellow wine is completely absorbed (about 4 h), place them in a frying pan, fry them dry over low heat, stir fry until the surface is dark brown or brown, remove when there is a slight burnt spot, spread out to dry, sieve to remove debris. Raw rhubarb: wine=10:1 | 0.1% sodium bicarbonate solution | Reversed phase high performance liquid chromatography | — | Sennoside A; sennoside B |
| Zhang et al., 2022 | Mix raw rhubarb slices with yellow wine (100:10), moisten thoroughly, fry dry with low heat until the surface is dark brown and yellow with slightly burnt spots, remove and let it cool | 70% methanol | High performance liquid chromatography | Rhein; emodin; aloe-emodin; physcion | Chrysophanol |
|  |  |  |  | Gallic acid; catechin | Aloe-emodin-8-O-β-D-glucoside; rhein-8-O-β-D-glucoside; emodin-8-O-β-D-glucoside; physcion-8-O-β-D-glucoside |
|  |  |  |  | Polydatin; 4-(4'hydroxyphenyl)-2-butanone | Sennoside A; sennoside B |
| Li, 2011 | According to the processing methods specified in the *Chinese Pharmacopoeia*, prepared by Beijing Renwei Herbal Pieces Factory | Methanol | High performance liquid chromatography | Emodin-8-O-β-D-glucoside | Rhein; emodin; aloe-emodin; chrysophanol; physcion |
|  |  |  |  | Gallic acid; (+)-catechin | Aloe-emodin-8-O-β-D-glucoside; rhein-8-O-β-D-glucoside; aloe-emodin-3-CH2-O-β-D-glucoside |
|  |  |  |  | 4'-hydroxyphenyl-2-butane-4'-O-β-D-(6 "-O-galloyl)-glucoside; 4'-hydroxyphenyl-2-butane-4'-O-β-D-(6"-O-cinnamoyl)-glucoside | Trans-3,5,4'- trihydroxyvinyl-4'-O-β-D-glucoside; trans-3,5,4'- trihydroxystilbene-4'-O-β-D-(6"-O-galloyl)-glucoside |
|  |  |  |  |  | 4'-hydroxyphenyl-2-butanone; 4-hydroxyphenyl-2-butane-4'-O-β-D-(2"-O-galloyl-6"-O-(4''-hydroxy)-cinnamoyl)-glucoside |
| Wang et al., 2014 | Processed with rice wine (20% w/w) | Methanol | Ultra-performance liquid chromatography-quadrupole\time-of- flight mass spectrometry | Chrysophanol dimethyl ether | Emodin-8-O-glucoside; emodin-O-glucoside |
|  |  |  |  | Gallic acid-3-O-glucoside | Catechin-glucopyranoside; cinnamyl-galloyl-glucoside derivative |
|  |  |  |  | Torachrysone |  |
| Wang et al., 2015a | Directly purchased wine rhubarb after processing | 80% methanol; magnolol solution (IS, 200 μg/mL) | Ultra fast liquid chromatography with ion trap/time-of-flight mass spectrometry | — | Acetyl-chrysophanol-O-glucoside; 6-dehydroxylaccaic acid D-glucoside; acetyl-emodin -O-glucoside; acetyl-rhein-O-glucosid; emodin-O-(-6’-O-malonyl)-glucoside |
|  |  |  |  |  | (+)-catechin; (epi)catechin-O-gallate; cinnamoyl-O -glucose-O-galloyl; cinnamoyl-O -glucose-O-digalloyl |
|  |  |  |  |  | 6-hydroxyrumicin-8-O-glucoside |
| Wang et al., 2015b | Directly purchased wine rhubarb after processing | 80% methanol; naringenin solution (IS, 4 mg/mL) | High performance liquid chromatography coupled with tandem mass spectrometry | — | Rhein; aloe-emodin; Chrysophanol; Physcion |
|  |  |  |  |  | Rhein-8-O-b-D-glucoside; emodin-1-O-b-D-glucoside; aloe-emodin-8-O-b-D-glucoside |
|  |  |  |  |  | Sennoside A |

**References**

Li, H. F., Sun, Q., Wang, J. B., Jin, C., Xiao, X. H. (2011). Analysis on Change Law of Main Chemical Constituents of Rhubarb After Processing. *Journal of Shanxi University of Chinese Medicine*, 12, 14-17.

Li, L. (2011). *Study on the Variation Rules of Material Basis of Rhubarb after Processing*. doctor's thesis, China Academy of Chinese Medical Sciences.

Li, L., Zhang, C., Xiao, Y. Q., Chen, D. D., Tian, G. F., Wang, Y. (2010). Comparison of two butyrophenone constituents in 5 kinds of pieces of Dahuang (Radix et Rhizoma Rhei). *Journal of Beijing University of Traditional Chinese Medicine*, 33, 559-561.

Tian, G. F., Zhang, C., Li, L., Xiao, Y. Q., Chen, D. D., Wang, Y. (2010). Variety regulation of aloe-emodin-3-CH2-O-β-D-glucopyranoside and emodin-8-O-β-D-glucopyranoside in five processed pieces from Rheum palmatum. *China Journal of Chinese Materia Medica*, 35, 2437-2439.

Wang, M., Fu, J. F., Guo, H. M., Tian, Y., Xu, F. G., Song, R., et al. (2015a). Discrimination of crude and processed rhubarb products using a chemometric approach based on ultra fast liquid chromatography with ion trap/time-of-flight mass spectrometry. *J Sep Sci*, 38, 395-401. doi: 10.1002/jssc.201401044. Epub 2015 Jan 7. PMID: 25421806.

Wang, M., Tian, Y., Lv, M. Y., Xu, F. G., Zhang, Z. J., Song, R. (2015b). Targeted quantitative analysis of anthraquinone derivatives by high-performance liquid chromatography coupled with tandem mass spectrometry to discriminate between crude and processed rhubarb samples. *Analytical Methods*, 7, 5375-5380. doi: 10.1039/c5ay01067e.

Wang, Y., Li, L., Zhang, C., Xiao, Y. Q., Chen, D. D., Tian, G. F. (2010). Comparison of gallic acid and catechin contents in five processed products of Rheum palametum. *China Journal of Chinese Materia Medica*, 35, 2267-2269.

Wang, Z. H., Wang, D. M., Zheng, S. H., Wu, L. B., Huang, L. F., Chen, S. L. (2014). Ultra-performance liquid chromatography-quadrupole\time-of- flight mass spectrometry with multivariate statistical analysis for exploring potential chemical markers to distinguish between raw and processed Rheum palmatum. *BMC Complement Altern Med*, 14, 302. doi: 10.1186/1472-6882-14-302. PMID: 25128184; PMCID: PMC4147172.

Yan, Y. G., Yin, L. M., Wang, H. Y., Guo, L. L., Deng, C. (2016). Simultaneous Determination of 10 Kinds of Chemical Components in Processed Products of Rhei Radix et Rhizoma. *China Pharmacy*, 27, 3839-3842.

Yang, M., Xu, B. H., Wang, D. G., Chen, G. T. (2013). Effect of different processing methods on the content of five anthraquinones in Rhei Radix et Rhizoma. *Journal of Nantong University (Medical Sciences)*, 33, 385-387.

Yang, X. W., Li, J. S., Wu, D. K., Cai, B. C. (2012). Comparison of the Rhubarb and Processed Products on Eight Kinds of Components. *Chin Med J Res Prac*, 26, 73-75. doi:10.13728/j.1673-6427.2012.03.021.

Zeng, C., Lu, M. Y., Mo, T. T., Qin, Y. S., Huang, M. (2020). Processing of Dahuang(Rhubarb) and Establishment of Determination Methods of Sennanoside A and Sennanoside B in Different Processed Products. *Chinese Archives of Traditional Chinese Medicine*, 38, 47-52+263. doi:10.13193/j.issn.1673-7717.2020.11.013.

Zhang, Q., Chen, Y. Y., Yue, S. J., Wang, W. X., Zhao, C. B., Song, Y. J., et al. (2022). Study on the content changes of 16 chemical components in Radix et Rhizoma Rhei and its different processed products. *Chin J Tradit Chin Med Pharm*, 37, 1036-1040.
