# Supplementary material for: The application of rhubarb concoctions in traditional Chinese medicine and its compounds, processing methods, pharmacology, toxicology and clinical research
Source: Front Pharmacol. 2024 Aug 7;15:1442297. doi: 10.3389/fphar.2024.1442297 (PMC11335691; doi:10.3389/fphar.2024.1442297)
Supplement: Supplementary file 8 [file Table4.docx]

Supplementary Material

# Supplementary Tables

**Supplementary Table 4 The compound changes of cooked rhubarb before and after processing.**

| **References** | **Processing methods** | **Solvent used for sample preparation** | **Identification methods** | **Differences in compounds compared to raw rhubarb (increased)** | **Differences in compounds compared to raw rhubarb (decreased)** |
| --- | --- | --- | --- | --- | --- |
| Yang et al., 2013 | Slice or cut rhubarb into pieces, mix with yellow wine for 1-2 hours until the wine is completely absorbed, put it into a stewing jar or suitable container, seal it, and stew over water for 24-32 hours or steam in a wooden container until both inside and outside are black. Remove and dry in the sun. Processed in a ratio of 10:3 between rhubarb and yellow wine | Methanol; 10% hydrochloric acid; chloroform | High performance liquid chromatography | Emodin | Rhein; chrysophanol; physcion; aloe-emodin |
| Huang et al., 2023 | Clean rhubarb chunks are mixed with yellow wine, moistened until the wine is completely absorbed. Remove and steam in a steamer until both inside and outside turn black. Remove and dry at 50 ℃, then let it cool | 80% methanol | High performance liquid chromatography | — | Aloe-emodin-8-O-β-D-glucoside; rhein-8-O-β-D-glucoside; emodin-8-O-β-D-glucopyranoside; chrysophanol-1-O-β-D-glucoside; physcion-8-O-β-D-glucoside |
|  |  |  |  |  | Sennoside A |
|  |  |  |  |  | Catechin; (-)-epicatechin 3-O-gallate |
| Wang et al., 2022 | Mix rhubarb slices with yellow wine (50 kg of wine for 100 kg rhubarb slices), moisten for 1-2 hours until the yellow wine is sucked up, put into a stainless steel lunch box, sealed and steamed for 24 hours until the surface and inside are black, take out and dry | Tannins: water  Total anthraquinones: methanol; 8% hydrochloric acid; chloroform  Free anthraquinones: methanol | Ultraviolet spectrophotometry； High performance liquid chromatography | Free anthraquinones | Combined anthraquinones |
|  |  |  |  |  | Tannins |
| Li et al., 2010 | According to the processing methods specified in the *Chinese Pharmacopoeia(2005 edition)* | 50% methanol | High performance liquid chromatography | — | 4'-hydroxyphenyl-2-butanone; 4'-hydroxyphenyl-2-butanone-4'-O-β-D-(6"-galloyl)-glucoside |
| Wang et al., 2010 | According to the processing methods specified in the *Chinese Pharmacopoeia* and the *National Standards for the Processing of Traditional Chinese Medicine*, prepared by Beijing Renwei Herbal Pieces Factory | 100% methanol | High performance liquid chromatography | Gallic acid | — |
| Tian et al., 2010 | According to the processing methods specified in the *Chinese Pharmacopoeia* and the *National Standards for the Processing of Traditional Chinese Medicine*, prepared by Beijing Renwei Decoction Pieces Factory | Methanol | High performance liquid chromatography | — | Aloe-emodin-3-CH2-O-β-D-glucoside |
| Li et al., 2011 | According to the processing methods specified in the *Beijing Standards for the Processing of Traditional Chinese Medicine decoction pieces(2006 edition)*, prepared by Beijing Qiancao Decoction Pieces Co., Ltd | Tannins: water  Anthraquinone: methanol; 8% hydrochloric acid; chloroform | Anthraquinone components by ultra-high performance liquid chromatography, and tannin components by colorimetry | Total free anthraquinones;  emodin; aloe-emodin; chrysophanol | Rhein; physcion |
|  |  |  |  |  | Total combined anthraquinones |
|  |  |  |  |  | Total tannins |
| Li, 2011 | According to the processing methods specified in the *Chinese Pharmacopoeia*, prepared by Beijing Renwei Herbal Pieces Factory | Methanol | High performance liquid chromatography | Rhein; emodin; aloe-emodin; chrysophanol; physcion | Aloe-emodin-8-O-β-D-glucoside; rhein-8-O-β-D-glucoside; emodin-8-O-β-D-glucoside; Aloe-emodin-3-CH2-O-β-D-glucoside |
|  |  |  |  |  | Trans-3,5,4'-trihydroxystilbene-4'-O-β-D-(6"-O-galloyl)-glucoside; trans-3,5,4'-trihydroxystilbene-4'-O-β-D-glucoside |
|  |  |  |  | Gallic acid | 4'-hydroxyphenyl-2-butanone;  4'-hydroxyphenyl-2-butanone-4'-O-β-D-(6"-galloyl)-glucoside; 4'-hydroxyphenyl-2-butane-4'-O-β-D-(6"-O-cinnamoyl)-glucoside; 4'-hydroxyphenyl-2-butane-4'-O-β-D-(2"-O-Galloyl-6"-O-(4'-hydroxy)-cinnamoyl)-glucoside |
|  |  |  |  |  | (+)-catechin |
| Yang et al., 2012 | Mix raw rhubarb slices or chunks with yellow wine, seal for 1.5 hours until the wine is sucked up, put in a steamer, steam for 24 hours, remove and dry. For every 100kg of rhubarb chunks or pieces, use 30kg of yellow wine | Methanol | High performance liquid chromatography | Rhein; emodin; aloe-emodin; chrysophanol; physcion。 | — |
| Zhao et al., 2014 | Steam under high pressure at 120 ℃ for 3 hours, add 30mL of yellow wine to every 100g of rhubarb | 50% methanol | Ultra-performance liquid chromatography-quadrupole\time-of- flight mass spectrometry | — | Rhein; emodin; aloe-emodin; physcion |
|  |  |  |  |  | Emodin-8-O-(6'-O-malonyl)-glucoside; lac acid-D-8-O-(6'-O-cinnamyl)-glucoside |
|  |  |  |  |  | Gallic acid-3-O-glucoside/gallic acid-4-O-glucoside; catechin-glucoside; lindleyin;  gallic acid-3-O-(6'-O-gallyl)-glucoside/gallic acid-4-O-(6'-O-gallyl)-glucoside;  protocentaurin B5; catechins/epicatechins;  1-O-galloyl-2-O-cinnamoyl-β-D-glucoside;  2-cinnamoyl-1,6-digalloyl-β-D-glucoside/6-cinnamoyl-1,2-digalloylglucoside |
| Yan et al., 2016 | According to the processing method of rhubarb in the *Science of Chinese Medicine Processing* | Water; methanol | High performance liquid chromatography | Gallic acid | Rhein; emodin; aloe-emodin; chrysophanol; physcion |
|  |  |  |  |  | Chrysophanol-1-O-glucoside; emodin-8-O-glucoside |
|  |  |  |  |  | Sennoside B |
|  |  |  |  |  | Catechin |
| Zeng et al., 2020 | Mix the cleaned rhubarb slices with yellow wine, seal them until all the yellow wine is absorbed, put them into a steaming container, seal it, and heat it over high heat in a pot for 4-6 hours until both the inside and outside of the rhubarb are black brown. Take them out, sun dry or dry them. Rhubarb: yellow wine=10:2-3 | 0.1% sodium bicarbonate solution | Reversed phase high performance liquid chromatography | — | Sennoside A; Sennoside B |
| Han, 2021 | Clean rhubarb slices mixed with 50% yellow wine, sealed for 2 hours. After the yellow wine is completely absorbed by rhubarb, seal and steam for 24 hours until the inside turns yellow brown, and air dry | 8 times purified water | High performance liquid chromatography | Rhein; emodin; aloe-emodin; chrysophanol; physcion | — |
| Song et al., 2021 | Put 50g of rhubarb slices into a beaker, break them into small pieces, add yellow wine (diluted with 20g of deionized water for every 10g of yellow wine), mix well, let it moisten for 1.5h, wait for the yellow wine to be sucked up by rhubarb, seal with cling film and tin foil, and steam over water for 20h | Methanol | High performance liquid chromatography | Emodin; aloe-emodin; chrysophanol; physcion | ω-citreorosein |
|  |  |  |  |  | Aloe-emodin-8-O-glucoside; rhein-8-O-glucoside; emodin-8-O-glucoside |
|  |  |  |  | Gallic acid | Catechin; epicatechin; ethyl gallate |
|  |  |  |  |  | Trans-3,5,4'-trihydroxystyrene-4'-O-β-D-glucoside |
|  |  |  |  | 5-HMF | 4'-hydroxyphenyl-2-butanone-4'-O-β-D-[2ʺ-O-galloyl-6ʺ-O-(4ʺ-hydroxy)-cinnamoyl]-glucoside |
| Su et al., 2021 | Processed based on the processing method of *Chinese Pharmacopoeia (volume IV, 2015 edition)* | 70% ethanol | Ultraviolet-visible spectrophotometer method | — | Total flavone |
| Zhang et al., 2022 | Mix the raw rhubarb slices with yellow wine (100:30), seal and moisten until fully saturated, place in a steaming container, steam until both inside and outside turn black, remove and let cool, dry | 70% methanol | High performance liquid chromatography | Gallic acid; catechin | Rhein; emodin; aloe-emodin; chrysophanol; physcion |
|  |  |  |  | Polydatin; 4-(4'hydroxyphenyl)-2-butanone | Aloe-emodin-8-O-β-D-glucoside; rhein-8-O-β-D-glucoside; emodin-8-O-β-D-glucoside; chrysophanol-8-O-β-D-glucoside; physcion-8-O-β-D-glucoside |
|  |  |  |  |  | Sennoside A; Sennoside B |
| Zhou, 2022 | According to the processing methods specified in the *Chinese Pharmacopoeia(2015 edition)*, prepared by Beijing Renwei Herbal Pieces Factory | 75% ethanol; methanol | Ultra performance liquid chromatography | Rhein; emodin; aloe-emodin; chrysophanol; physcion | Aloe-emodin-8-O-β-D-glucoside; rhein-8-O-β-D-glucoside; emodin-8-O-β-D-glucoside |
|  |  |  |  |  | Ethyl gallate |
|  |  |  |  |  | Lindleyin; isolindleyin;  4'-hydroxyphenyl-2-butane-4'-O-β-D-(2"-Galloyl-6"-O-(4'-hydroxy)-cinnamoyl) |
| Zhu et al., 2016 | Directly purchased cooked rhubarb after processing | Water; methanol | Ultra-performance liquid chromatography-quadrupole\time-of- flight mass spectrometry | Rhein | Emodin |
|  |  |  |  |  | Aloe-emodin-1-O-β-D-glucopyranoside/aloe-emodin-8-O-β-D-glucopyranoside; emodin-8-O-β-D-glucopyranoside; emodin-8-O-(6'-O-malonyl)-glucopyranoside; 6-methyl-aloe-emodin |
|  |  |  |  |  | Sennoside A; Sennoside B; Sennoside C; Sennoside D |
|  |  |  |  |  | Gallic acid-3-O-β-D-(6'-O-galloyl)-glucopyranoside/gallic acid-4-O-β-D-(6'-O-galloyl)-glucopyranoside  Catechin; dimer of catechin;  epicatechin-(4β-8)-epicatechin-(4β-8)-catechin/epicatechin-(4β-6)-epicatechin-(4β-8)-catechin/epicatechin-(4β-8)-epicatechin-(4β-6)-catechin; (-)epicatechin-3-O-gallate;  2-O-p-coumaroyl-1-O-galloy-β-D-glucose; 2-O-cinnamoyl-1-O-galloy-β-D-glucose / 1-O-galloy-6-O-cinnamoyl-β-D-glucose; cassialoin |
| Wang et al., 2014 | Processed with rice wine (20% w/w), steam until both inside and outside are black | Methanol | Ultra-performance liquid chromatography-quadrupole\time-of- flight mass spectrometry | Chrysophanol dimethyl ether | Emodin-8-O-glucoside; emodin-O-glucoside |
|  |  |  |  | Gallic acid-3-O-glucoside | Catechin-glucopyranoside; cinnamyl-galloyl-glucoside derivative |
|  |  |  |  | Torachrysone |  |
| Wang et al., 2015a | Directly purchased cooked rhubarb after processing | 80% methanol; magnolol solution (IS, 200 μg/mL) | Ultra fast liquid chromatography with ion trap/time-of-flight mass spectrometry | — | 6-dehydroxylaccaic acid D; rhein; emodin |
|  |  |  |  |  | Acetyl-chrysophanol-O-glucoside;  6-dehydroxylaccaic acid D-glucoside;  emodin-O-glucoside; acetyl-emodin-O-glucoside; acetyl-rhein-O-glucoside; emodin-O-(-6’-O-malonyl)-glucoside |
|  |  |  |  |  | (+)-catechin; (epi)catechin-O-gallate; cinnamoyl-O-glucose-O-galloyl; cinnamoyl-O-glucose-O-digalloyl |
|  |  |  |  |  | 6-hydroxyrumicin-8-O-glucoside; torachrysone-8-O-glucoside |
| Wang et al., 2015b | Directly purchased cooked rhubarb after processing | 80% methanol; naringenin solution (IS, 4 mg/mL) | High performance liquid chromatography coupled with tandem mass spectrometry | — | Rhein; emodin; aloe-emodin; chrysophanol; physcion |
|  |  |  |  |  | Emodin-1-O-b-D-glucoside; aloe-emodin-8-O-b-D-glucoside |
|  |  |  |  |  | Sennoside A; Sennoside B |

**References**

Han, H. F. (2021). Correlation Between Anthraquinone and Antibacterial Activity in Different Processed Products of Dahuang (Rhubarb) by Multiple Regression Analysis. *Guiding Journal of Traditional Chinese Medicine and Pharmacy*, 27, 65-68. doi:10.13862/j.cnki.cn43-1446/r.2021.01.014.

Huang, K. W., Zhang, H., Zhao, W. Z., Zheng, X. Y., Hu, Y., Tan, P. (2023). Study on the difference of components in raw and steamed products of Rheum tanguticum Maxim. ex Balf. *Journal of Guangdong Pharmaceutical University*, 39, 77-86. doi:10.16809/j.cnki.2096-3653.2023040308.

Li, H. F., Sun, Q., Wang, J. B., Jin, C., Xiao, X. H. (2011). Analysis on Change Law of Main Chemical Constituents of Rhubarb After Processing. *Journal of Shanxi University of Chinese Medicine*, 12, 14-17.

Li, L. (2011). *Study on the Variation Rules of Material Basis of Rhubarb after Processing*. doctor's thesis, China Academy of Chinese Medical Sciences.

Li, L., Zhang, C., Xiao, Y. Q., Chen, D. D., Tian, G. F., Wang, Y. (2010). Comparison of two butyrophenone constituents in 5 kinds of pieces of Dahuang (Radix et Rhizoma Rhei). *Journal of Beijing University of Traditional Chinese Medicine*, 33, 559-561.

Song, Y. N., Wang, Y., Gao, Y., Zheng, Y. H., Liu, T. L.Zhang, C. (2021). Analysis on Quality Transfer Law of Rhei Radix et Rhizoma Steamed with Rice-wine During Processing Based on Correlation Between External Appearance Color and Internal Component. *Chinese Journal of Experimental Traditional Medical Formulae*, 27, 157-164. doi:10.13422/j.cnki.syfjx.20211758.

Su, H. Z., Xie, Z., Wei, J. C., Tan, Q. Y., Luo, Y. L., Zhong, W., et al. (2021). Comparative Study on the Contents of Total Flavonoids Contained in Raw Rhubarb, Steamed and Processed Rhubarb. *Western Journal of Traditional Chinese Medicine*, 34, 28-31.

Tian, G. F., Zhang, C., Li, L., Xiao, Y. Q., Chen, D. D., Wang, Y. (2010). Variety regulation of aloe-emodin-3-CH2-O-β-D-glucopyranoside and emodin-8-O-β-D-glucopyranoside in five processed pieces from Rheum palmatum. *China Journal of Chinese Materia Medica*, 35, 2437-2439.

Wang, M., Fu, J. F., Guo, H. M., Tian, Y., Xu, F. G., Song, R., et al. (2015a). Discrimination of crude and processed rhubarb products using a chemometric approach based on ultra fast liquid chromatography with ion trap/time-of-flight mass spectrometry. *J Sep Sci*, 38, 395-401. doi: 10.1002/jssc.201401044. Epub 2015 Jan 7. PMID: 25421806.

Wang, M., Han, T., Li, C. S., Xu, W. J., Yang, L. L., Zhang, S. Y., et al. (2022). Chemical Components and Toxicity of Radix et Rhizoma Rhei before and after Processing. *World Chinese Medicine*, 17, 3131-3138.

Wang, M., Tian, Y., Lv, M. Y., Xu, F. G., Zhang, Z. J., Song, R. (2015b). Targeted quantitative analysis of anthraquinone derivatives by high-performance liquid chromatography coupled with tandem mass spectrometry to discriminate between crude and processed rhubarb samples. *Analytical Methods*, 7, 5375-5380. doi: 10.1039/c5ay01067e.

Wang, Y., Li, L., Zhang, C., Xiao, Y. Q., Chen, D. D., Tian, G. F. (2010). Comparison of gallic acid and catechin contents in five processed products of Rheum palametum. *China Journal of Chinese Materia Medica*, 35, 2267-2269.

Wang, Z. H., Wang, D. M., Zheng, S. H., Wu, L. B., Huang, L. F., Chen, S. L. (2014). Ultra-performance liquid chromatography-quadrupole\time-of- flight mass spectrometry with multivariate statistical analysis for exploring potential chemical markers to distinguish between raw and processed Rheum palmatum. *BMC Complement Altern Med*, 14, 302. doi: 10.1186/1472-6882-14-302. PMID: 25128184; PMCID: PMC4147172.

Yan, Y. G., Yin, L. M., Wang, H. Y., Guo, L. L., Deng, C. (2016). Simultaneous Determination of 10 Kinds of Chemical Components in Processed Products of Rhei Radix et Rhizoma. *China Pharmacy*, 27, 3839-3842.

Yang, M., Xu, B. H., Wang, D. G., Chen, G. T. (2013). Effect of different processing methods on the content of five anthraquinones in Rhei Radix et Rhizoma. *Journal of Nantong University (Medical Sciences)*, 33, 385-387.

Yang, T., Hu, C. J., Zhou, Y. C., Wang, H., Long, L. Y.Wu, W. H. (2012). Comparative Study on HPLC Fingerprints of Stewed Rhubarb and Rhubarb. *Chin Med J Res Prac*, 26, 29-31+51. doi:10.13728/j.1673-6427.2012.06.017.

Zeng, C., Lu, M. Y., Mo, T. T., Qin, Y. S., Huang, M. (2020). Processing of Dahuang(Rhubarb) and Establishment of Determination Methods of Sennanoside A and Sennanoside B in Different Processed Products. *Chinese Archives of Traditional Chinese Medicine*, 38, 47-52+263. doi:10.13193/j.issn.1673-7717.2020.11.013.

Zhang, Q., Chen, Y. Y., Yue, S. J., Wang, W. X., Zhao, C. B., Song, Y. J., et al. (2022). Study on the content changes of 16 chemical components in Radix et Rhizoma Rhei and its different processed products. *Chin J Tradit Chin Med Pharm*, 37, 1036-1040.

Zhao, N., Zhang, X. Z., Hu, C. J., Jia, T. Z.Xiao, H. B. (2014). Metabolomics analysis revealing multiple compounds changed in rhubarb after processing. *China Journal of Chinese Materia Medica*, 39, 1607-1613.

Zhou, P. (2022). *Pharmacodynamic effect of "laxative followed by astringent" of raw rhubarb and mechanism studies based on metabolomics*. master's thesis, China Academy of Chinese Medical Sciences. doi:10.27658/d.cnki.gzzyy.2022.000144.

Zhu, T. T., Liu, X., Wang, X. L., Cao, G., Qin, K. M., Pei, K., et al. (2016). Profiling and analysis of multiple compounds in rhubarb decoction after processing by wine steaming using UHPLC-Q-TOF-MS coupled with multiple statistical strategies. *J Sep Sci*, 39, 3081-90. doi: 10.1002/jssc.201600256. Epub 2016 Jul 12. PMID: 27291339.
