# Supplementary material for: The application of rhubarb concoctions in traditional Chinese medicine and its compounds, processing methods, pharmacology, toxicology and clinical research
Source: Front Pharmacol. 2024 Aug 7;15:1442297. doi: 10.3389/fphar.2024.1442297 (PMC11335691; doi:10.3389/fphar.2024.1442297)
Supplement: Supplementary file 9 [file Table6.DOCX]

Supplementary Material

# Supplementary Tables

**Supplementary Table 6 Study quality list.**

| **Study** | **(1)** | **(2)** | **(3)** | **(4)** | **(5)** |
| --- | --- | --- | --- | --- | --- |
| Huang et al., 2023 | + | + | - | + | - |
| Wang et al., 2022 | - | + | + | + | - |
| Li et al., 2010 | ± | + | + | - | - |
| Wang et al., 2010 | + | + | + | - | - |
| Tian et al., 2010 | + | + | + | - | - |
| Li et al., 2011 | - | - | + | - | - |
| Yang et al., 2012 | + | + | - | - | - |
| Yang et al., 2013 | + | + | + | - | - |
| Zhao et al., 2014 | + | - | - | + | - |
| Yan et al., 2016 | + | + | + | - | - |
| Zeng et al., 2020 | + | + | + | - | - |
| Han, 2021 | - | + | + | - | - |
| Song et al., 2021 | ± | - | - | + | - |
| Su et al., 2021 | - | + | + | - | - |
| Zhang et al., 2022 | - | + | + | + | - |
| Zhou, 2022 | + | + | + | - | - |
| Li, 2011 | + | + | + | - | - |
| Yang et al., 2012 | ± | + | + | - | - |
| Sun et al., 2024 | - | + | - | + | - |
| Yang et al., 2023 | - | + | - | + | - |
| Nan et al., 2019 | + | + | + | - | - |
| Cai et al., 2018 | - | - | + | - | - |
| Yang et al., 2020 | - | + | + | - | - |
| Zhu et al., 2016 | + | ± | - | + | - |
| Wang et al., 2014 | + | - | - | + | + |
| Wang et al., 2015a | + | ± | - | + | + |
| Wang et al., 2015b | + | + | - | + | - |

**Notes: (1) Optimized the content determination method; (2) Conducted precision, stability, and repeatability tests for detection; (3) Reported the specific values measured in the results section; (4) Reported statistical methods; (5) Potential conflict of interest Statement.**

**"+" indicates that the item is "yes", "-" indicates that the item is "unclear" or "no", and "±" indicates that the item is "partly yes" [mainly for the two items (1) and (2), such as only the chromatographic conditions were optimized without the sample preparation conditions, or only one of the precision, stability, and repeatability tests was reported].**

**References**

Cai, X. J., Xu, H. X., Lin, S. H., Yu, N. C. (2018). Separation and identification of the new chemical constituent from rhubarb charcoal and its formational rule. *Chinese Journal of Hospital Pharmacy*, 38, 2336-2339. doi:10.13286/j.cnki.chinhosppharmacyj.2018.22.09.

Han, H. F. (2021). Correlation Between Anthraquinone and Antibacterial Activity in Different Processed Products of Dahuang (Rhubarb) by Multiple Regression Analysis. *Guiding Journal of Traditional Chinese Medicine and Pharmacy*, 27, 65-68. doi:10.13862/j.cnki.cn43-1446/r.2021.01.014.

Huang, K. W., Zhang, H., Zhao, W. Z., Zheng, X. Y., Hu, Y., Tan, P. (2023). Study on the difference of components in raw and steamed products of Rheum tanguticum Maxim. ex Balf. *Journal of Guangdong Pharmaceutical University*, 39, 77-86. doi:10.16809/j.cnki.2096-3653.2023040308.

Li, H. F., Sun, Q., Wang, J. B., Jin, C., Xiao, X. H. (2011). Analysis on Change Law of Main Chemical Constituents of Rhubarb After Processing. *Journal of Shanxi University of Chinese Medicine*, 12, 14-17.

Li, L. (2011). *Study on the Variation Rules of Material Basis of Rhubarb after Processing*. doctor's thesis, China Academy of Chinese Medical Sciences.

Li, L., Zhang, C., Xiao, Y. Q., Chen, D. D., Tian, G. F., Wang, Y. (2010). Comparison of two butyrophenone constituents in 5 kinds of pieces of Dahuang (Radix et Rhizoma Rhei). *Journal of Beijing University of Traditional Chinese Medicine*, 33, 559-561.

Nan, J. H., Zhang, X. S., Hua, Y. L., Wei, Y. M. (2019). Study on Variations of Five Chemical Compositions in Raw Rhubarb and Charred Rhubarb. *Progress in Veterinary Medicine*, 40, 58-63. doi:10.16437/j.cnki.1007-5038.2019.04.011.

Song, Y. N., Wang, Y., Gao, Y., Zheng, Y. H., Liu, T. L.Zhang, C. (2021). Analysis on Quality Transfer Law of Rhei Radix et Rhizoma Steamed with Rice-wine During Processing Based on Correlation Between External Appearance Color and Internal Component. *Chinese Journal of Experimental Traditional Medical Formulae*, 27, 157-164. doi:10.13422/j.cnki.syfjx.20211758.

Su, H. Z., Xie, Z., Wei, J. C., Tan, Q. Y., Luo, Y. L., Zhong, W., et al. (2021). Comparative Study on the Contents of Total Flavonoids Contained in Raw Rhubarb, Steamed and Processed Rhubarb. *Western Journal of Traditional Chinese Medicine*, 34, 28-31.

Sun, J., Xu, W. J., Zhong, L. Y., Chen, J. B., DONG, L. (2024). UPLC-QE-Orbitrap-MS combined with network pharmacology to explore differential components and mechanisms of raw and scorched rhubarb for treatment of ulcerative colitis. *China Journal of Chinese Materia Medica*, 49, 1834-1847. doi:10.19540/j.cnki.cjcmm.20231226.302.

Tian, G. F., Zhang, C., Li, L., Xiao, Y. Q., Chen, D. D., Wang, Y. (2010). Variety regulation of aloe-emodin-3-CH2-O-β-D-glucopyranoside and emodin-8-O-β-D-glucopyranoside in five processed pieces from Rheum palmatum. *China Journal of Chinese Materia Medica*, 35, 2437-2439.

Wang, M., Fu, J. F., Guo, H. M., Tian, Y., Xu, F. G., Song, R., et al. (2015a). Discrimination of crude and processed rhubarb products using a chemometric approach based on ultra fast liquid chromatography with ion trap/time-of-flight mass spectrometry. *J Sep Sci*, 38, 395-401. doi: 10.1002/jssc.201401044. Epub 2015 Jan 7. PMID: 25421806.

Wang, M., Han, T., Li, C. S., Xu, W. J., Yang, L. L., Zhang, S. Y., et al. (2022). Chemical Components and Toxicity of Radix et Rhizoma Rhei before and after Processing. *World Chinese Medicine*, 17, 3131-3138.

Wang, M., Tian, Y., Lv, M. Y., Xu, F. G., Zhang, Z. J., Song, R. (2015b). Targeted quantitative analysis of anthraquinone derivatives by high-performance liquid chromatography coupled with tandem mass spectrometry to discriminate between crude and processed rhubarb samples. *Analytical Methods*, 7, 5375-5380. doi: 10.1039/c5ay01067e.

Wang, Y., Li, L., Zhang, C., Xiao, Y. Q., Chen, D. D., Tian, G. F. (2010). Comparison of gallic acid and catechin contents in five processed products of Rheum palametum. *China Journal of Chinese Materia Medica*, 35, 2267-2269.

Wang, Z. H., Wang, D. M., Zheng, S. H., Wu, L. B., Huang, L. F., Chen, S. L. (2014). Ultra-performance liquid chromatography-quadrupole\time-of- flight mass spectrometry with multivariate statistical analysis for exploring potential chemical markers to distinguish between raw and processed Rheum palmatum. *BMC Complement Altern Med*, 14, 302. doi: 10.1186/1472-6882-14-302. PMID: 25128184; PMCID: PMC4147172.

Yan, Y. G., Yin, L. M., Wang, H. Y., Guo, L. L., Deng, C. (2016). Simultaneous Determination of 10 Kinds of Chemical Components in Processed Products of Rhei Radix et Rhizoma. *China Pharmacy*, 27, 3839-3842.

Yang, L., Wen, Y. X., Liu, Y., Cheng, Y. R., Shi, X. J., Gong, Y. T., et al. (2020). Study on relationship between color characteristics of rhubarb charcoal in heating process and contents of 14 chemical components. *China Journal of Chinese Materia Medica*, 45, 4230-4237. doi:10.19540/j.cnki.cjcmm.20200622.307.

Yang, L., Yang, D. P., Sun, J., Dong, L.Chen, J. B. (2023). Study on the Variation Patterns of the Potential Q-Markers for the Efficacy Enhancement and Toxicity Attenuation during the Scorching Process of Rhubarb Charcoal. *Guiding Journal of Traditional Chinese Medicine and Pharmacy*, 29, 66-73. doi:10.13862/j.cn43-1446/r.2023.11.012.

Yang, M., Xu, B. H., Wang, D. G., Chen, G. T. (2013). Effect of different processing methods on the content of five anthraquinones in Rhei Radix et Rhizoma. *Journal of Nantong University (Medical Sciences)*, 33, 385-387.

Yang, T., Hu, C. J., Zhou, Y. C., Wang, H., Long, L. Y.Wu, W. H. (2012). Comparative Study on HPLC Fingerprints of Stewed Rhubarb and Rhubarb. *Chin Med J Res Prac*, 26, 29-31+51. doi:10.13728/j.1673-6427.2012.06.017.

Yang, X. W., Li, J. S., Wu, D. K., Cai, B. C. (2012). Comparison of the Rhubarb and Processed Products on Eight Kinds of Components. *Chin Med J Res Prac*, 26, 73-75. doi:10.13728/j.1673-6427.2012.03.021.

Zeng, C., Lu, M. Y., Mo, T. T., Qin, Y. S., Huang, M. (2020). Processing of Dahuang(Rhubarb) and Establishment of Determination Methods of Sennanoside A and Sennanoside B in Different Processed Products. *Chinese Archives of Traditional Chinese Medicine*, 38, 47-52+263. doi:10.13193/j.issn.1673-7717.2020.11.013.

Zhang, Q., Chen, Y. Y., Yue, S. J., Wang, W. X., Zhao, C. B., Song, Y. J., et al. (2022). Study on the content changes of 16 chemical components in Radix et Rhizoma Rhei and its different processed products. *Chin J Tradit Chin Med Pharm*, 37, 1036-1040.

Zhao, N., Zhang, X. Z., Hu, C. J., Jia, T. Z.Xiao, H. B. (2014). Metabolomics analysis revealing multiple compounds changed in rhubarb after processing. *China Journal of Chinese Materia Medica*, 39, 1607-1613.

Zhou, P. (2022). *Pharmacodynamic effect of "laxative followed by astringent" of raw rhubarb and mechanism studies based on metabolomics*. master's thesis, China Academy of Chinese Medical Sciences. doi:10.27658/d.cnki.gzzyy.2022.000144.

Zhu, T. T., Liu, X., Wang, X. L., Cao, G., Qin, K. M., Pei, K., et al. (2016). Profiling and analysis of multiple compounds in rhubarb decoction after processing by wine steaming using UHPLC-Q-TOF-MS coupled with multiple statistical strategies. *J Sep Sci*, 39, 3081-90. doi: 10.1002/jssc.201600256. Epub 2016 Jul 12. PMID: 27291339.
