# Supplementary material for: The application of rhubarb concoctions in traditional Chinese medicine and its compounds, processing methods, pharmacology, toxicology and clinical research
Source: Front Pharmacol. 2024 Aug 7;15:1442297. doi: 10.3389/fphar.2024.1442297 (PMC11335691; doi:10.3389/fphar.2024.1442297)
Supplement: Supplementary file 10 [file Table10.docx]

Supplementary Material

# Supplementary Tables

**Supplementary Table 10 Clinical applications of different rhubarb concoctions.**

| **Disease** | **Rhubarb state or combination therapy** | **Experimental subject (Case)** | **Research design** | **Grouping and number of people** | | **Treatment, method** | | **Course of treatment** | **Result** | **References** |
| --- | --- | --- | --- | --- | --- | --- | --- | --- | --- | --- |
|  |  |  |  | **Treatment group** | **Control group** | **Treatment group** | **Control group** |  |  |  |
| Constipation | Rhubarb Extract (processing method unknown) | 42 | Double-blind randomized placebo-controlled trial | Low dose: 14  High dose: 14 | 14 | Low dose: Re×112.5 mg anthraquinone derivatives  High dose: Re×225 mg anthraquinone derivatives | Placebo: maltodextrin | 30 days | Daily oral supplementation of RE for 30 days was safe even at the higher dose. Stool frequency and consistency, and perceived change in transit problem, transit speed and difficulty in evacuating, investigated by validated questionnaires, were improved in both groups of RE-treated volunteers compared to placebo. RE supplementation promotes butyrate-producing bacteria and SCFA, an effect that could contribute to relieving chronic constipation in middle-aged persons. | (Neyrinck et al., 2022) |
| Severe acute pancreatitis | Compound formula (processing method unknown) | 80 | Randomized controlled trial | 40 | 40 | Increase Dachengqi Decoction enema on the basis of the control group. | On the basis of conventional treatment plans, add somatostatin and ulinastatin. | 1 week | After 7 days of treatment, the total effective rate of the study group was higher than that of the control group (P<0.05); The total score of main symptoms, secondary symptoms, and TCM syndromes, PAMY, LPS, GAS, TNF - α, PCT, and CRP levels in both groups of patients decreased compared to before treatment, and the study group was lower than the control group. The MTL and VIP levels in both groups significantly increased compared to before treatment, and the study group was significantly higher than the control group. There was no statistically significant difference in the incidence of adverse events between the two groups. | (Xiu et al., 2023) |
| Parkinson's functional constipation | Single drug (raw rhubarb powder) | 100 | Randomized controlled trial | 50 | 50 | Application of rhubarb powder on Shenque acupoint for treatment. | Maren Soft Capsules | 2 weeks | The overall effectiveness rate of the treatment group was higher than that of the control group (P<0.05); The constipation symptom score of the treatment group was lower than that of the control group (P<0.05). | (Fan et al., 2015) |
| Adhesive intestinal obstruction | Compound formula (processing method unknown) | 67 | Retrospective study | 35 | 32 | On the basis of the control group, apply rhubarb and mirabilite powder around the navel. | Fasting water, gastrointestinal decompression, fluid replacement, anti infection, nutritional support, inhibition of gastrointestinal fluid secretion, sedation, spasmolysis, pain relief, etc. | Not mentioned. | The total effective rate of the treatment group was higher than that of the control group (P<0.01); The relief time of abdominal pain and spontaneous defecation time in the treatment group were significantly better than those in the control group (P<0.05); The percentage of neutrophils and the recovery of serum CRP in the treatment group were significantly better than those in the control group (P<0.05); The hospitalization time and hospitalization expenses of the treatment group were lower than those of the control group (P<0.05). | (Wang et al., 2018) |
| Postoperative gynecologic malignancies | Compound formula (processing method unknown) | 86 | Randomized controlled trial | 43 | 43 | Increase Dachengqi Decoction enema on the basis of the control group. | Conventional basic therapy | Twice，8h/12h after operation | Increase Dachengqi Decoction can promote the recovery of gastrointestinal function, improve urodynamics, and enhance patients' quality of life after extensive hysterectomy with preservation of pelvic autonomic nerves, with remarkable clinical efficacy. | (Gao and Wang, 2015) |
| Severe periodontitis with diabetes | Preparation of ointment using single drug extract (raw rhubarb) | 48 | Randomized controlled trial | 24 | 24 | Routine treatment of diabetes, subgingival scaling, root planing and rhubarb extract ointment filled periodontal pockets. | Routine treatment of diabetes, subgingival scaling and root planing. | 12 weeks | After 6 and 12 weeks of treatment, the depth of periodontal pockets, clinical attachment loss, and probing bleeding detection decreased in the control group and experimental group, with the experimental group significantly lower than the control group (P<0.05). | (Li et al., 2018) |
| Periappendiceal abscess in children | Compound formula (processing method unknown) | 142 | Retrospective study | 90 | 52 | Intravenous injection of antibiotics and oral administration of Rhubarb and Moutan Decoction. | Intravenous injection of antibiotics. | 1-2 weeks | The cure rate of the intervention group was significantly higher than that of the control group (P<0.05), and there was no statistically significant difference in the total time of intravenous antibiotic use, hospital stay, recurrence rate, surgical time, and postoperative complications between the two groups before appendectomy. | (Lin et al., 2023) |
| Chronic pelvic inflammatory disease | Compound formula (processing method unknown) | 80 | Randomized controlled trial | 40 | 40 | Oral administration of Rhubarb and Moutan Decoction. | Doxycycline and Cefoxitin. | 2 weeks | The total effective rate of the observation group was higher than that of the control group (P<0.05); The total score of the main symptoms, neutrophil ratio, white blood cell count, serum MCP-1 and IGF-1 levels in the observation group were lower than those in the control group, and the differences were statistically significant (P<0.05). | (Chen, 2021) |
| Severe pancreatitis complicated by abdominal infection | Single drug (raw rhubarb) | 84 | Randomized controlled trial | 42 | 42 | On the basis of the control group, rhubarb was given nasogastric feeding (taking 30g of raw rhubarb, boiling it in water to 200ml, cooling it down, feeding it through a nasogastric tube, clamping the gastric tube for 1 hour, and then receiving negative pressure drainage). | Routine treatment and Xuebijing injection. | 1 week | The relief time of abdominal pain symptoms, abdominal distension symptoms, and anal exhaust time in the observation group were shorter than those in the control group (P<0.05); The levels of WBC, CRP, PCT, IL-6, syndecan-1, HS, and HA in the observation group were significantly lower than those in the control group (P<0.05), and there was no significant difference in the levels of AMY between the two groups (P>0.05); The treatment effectiveness rate of the observation group was significantly higher than that of the control group (P<0.05). | (Xiang et al., 2021) |
| Nonvariceal upper gastrointestinal bleeding | Single drug (processing method unknown) | 80 | Retrospective study | 42 | 38 | Gastroscopy guided injection of adrenaline combined with spraying of ultra-fine rhubarb powder for treatment. | Epinephrine injection guided by gastroscopy. | Not applicable | The total effective rate of treatment in the observation group was significantly higher than that in the control group (P<0.05); The hemostasis time and hospitalization time in the observation group were significantly shorter than those in the control group (both P<0.05); The re-bleeding rate in the observation group was significantly lower than that in the control group (P<0.05); There was no statistically significant difference in the incidence of adverse reactions between the two groups. | (Zhou et al., 2015) |
| Nonvariceal upper gastrointestinal bleeding | Single drug (raw rhubarb) | 120 | Randomized controlled trial | 60 | 60 | Spraying ultra-fine rhubarb powder under gastroscopy. | Spraying norepinephrine under gastroscopy. | Not applicable | The hemostasis time in the treatment group was significantly shorter than that in the control group (P<0.01), and the 72 hour re bleeding rate in the treatment group was significantly lower than that in the control group (P<0.01); The treatment group did not experience any adverse reactions, while the control group had 2 cases of headache and 1 case of dizziness. | (He and Yin, 2014) |
| Interstitial pulmonary fibrosis | Compound formula (wine rhubarb) | 60 | Randomized controlled trial | 30 | 30 | Modified Xia Yu Xue Decoction combined with acetylcysteine. | Acetylcysteine | 6 months | The TCM syndrome score and quality of life score of the two groups after treatment decreased compared to before treatment (P<0.05), and the treatment group was better than the control group (P<0.05); After treatment, the value of carbon monoxide diffusion in the treatment group increased (P<0.05), while there was no significant change in various indicators of lung function in the control group (P>0.05). The value of carbon monoxide diffusion in the treatment group was higher than that in the control group (P<0.05); There was no statistically significant difference in high-resolution CT scores between the two groups before and after treatment, both within and between groups. | (An et al., 2017) |
| Primary liver cancer | Compound formula (cooked rhubarb) | 79 | Randomized controlled trial | 39 | 40 | Hepatoarterial chemoembolization combined with oral administration of Dahuang Zhechong Pill. | Hepatoarterial chemoembolization | 4 weeks | The total effective rate of the observation group was higher than that of the control group (P<0.05); After treatment, the immune function indicators such as serum CD4+cells, CD4+/CD8+cells, and CD3+cells in the observation group were higher than those in the control group (P<0.05); The serum levels of alpha fetoprotein, CA 199, alanine aminotransferase, and total bilirubin in the observation group were lower than those in the control group (P<0.05). The plasma levels of VEGF, TGF - β 1, and MMP-2 in the observation group were lower than those in the control group (P<0.05). The total incidence of adverse reactions in the observation group was lower than that in the control group (P<0.05). | (Dai et al., 2021) |
| Advanced ovarian cancer | Compound formula (cooked rhubarb) | 94 | Randomized controlled trial | 47 | 47 | On the basis of the control group, add Dahuang Zhechong Pill. | TP (Paclitaxel+Cisplatin) combined with Olaparib. | 6 months | The total effective rate of the treatment group was higher than that of the control group (P<0.05); The levels of CD3+, CD4+, and CD4+/CD8+in both groups increased (P<0.05), and the treatment group was better than the control group (P<0.01); The average decrease in serum tumor markers in both groups (P<0.05) was observed, and the treatment group was better than the control group (P<0.01); The scores of various quality of life indicators in both groups decreased (P<0.05), and the treatment group was better than the control group (P<0.01); The total incidence of adverse reactions in the treatment group was lower than that in the control group (P<0.05). | (Chen and Zhao, 2023) |
| Endometriosis | Compound formula (cooked rhubarb) | 67 | Randomized controlled trial | 34 | 33 | Dahuang Zhechong Capsules, 5 capsules bid | Mifepristone, 12.5 mg daily | 12 weeks | VAS score in the experimental group was lower than that in the control group (P<0.05). B&B multidimensional scale score in the experimental group was lower than that in the control group (P<0.01). Level of prostaglandin F_2a_ (PGF_2a_), prostaglandin E_2_ (PGE_2_) and thromboxane B_2_ (TXB_2_) of experimental group was lower than those of control group (P<0.05). But level of 6-keto-PGF1a of experimental group was higher than that of control group (P<0.01). | (Li, 2013) |
| Endometriosis | Compound formula (cooked rhubarb) | 126 | Randomized controlled trial | 62 | 64 | Dahuang Zhechong Pill+Deferiprone tablets | Sanjie Zhentong Pill+Deferiprone tablets | 3 menstrual cycles | On the basis of progesterone treatment, the use of Dahuang Zhechong Pill in the treatment of endometriosis pelvic pain patients can further reduce the symptoms related to pelvic pain, improve the quality of life of patients and clinical efficacy, and has the effect of regulating the levels of prostaglandins, matrix metalloproteinases and pro-inflammatory factors. | (Fan et al., 2019) |
| Non alcoholic fatty liver disease | Compound formula (processing method unknown) | 80 | Randomized controlled trial | 40 | 40 | On the basis of treatment for the control group patients, oral administration of Dahuang Lidan Capsules was given. | Basic treatment+oral administration of silybin. | 3 months | The AST, ALT, GGT, TC, TG, and LDL-C of the observation group patients were lower than those of the control group (P<0.01); The total effective rate of the observation group was significantly better than that of the control group (P<0.05). After treatment, the observation group had significantly higher levels of bifidobacteria and lactobacilli compared to the control group, and significantly lower levels of Escherichia coli compared to the control group (P<0.01). | (Li et al., 2021) |
| Non alcoholic fatty liver disease | Compound formula (cooked rhubarb) | 120 | Randomized controlled trial | TCM Group: 40 | 40 | TCM Group: Oral Yin Chen Hao Decoction. | Oral polyene phosphatidylcholine. | 2 months | After treatment, the levels of ALT, AST, TB, and ALP in each group were significantly reduced compared to before treatment (P<0.05), and the levels of various indicators in the combination group were significantly lower than those in the control group and traditional Chinese medicine group (P<0.05); After treatment, the levels of TG, TC, and LDL-c in each group were significantly reduced compared to before treatment (P<0.05), and the levels of various indicators in the combination group were significantly lower than those in the control group and traditional Chinese medicine group (P<0.05); After treatment, LBP, TBA, and TNF - α in each group decreased significantly compared to before treatment (P<0.05), and the levels of various indicators in the combination group were significantly lower than those in the control group and traditional Chinese medicine group after treatment (P<0.05). | (Gu, 2021) |
|  |  |  |  | Joint group: 40 |  | Oral administration of Yin Chen Hao Decoction and Bifidobacterium triple live bacteria. |  |  |  |  |
| Jaundiced hepatitis | Single drug (raw rhubarb powder) | 103 | Randomized controlled trial | 52 | 51 | On the basis of the application of diammonium glycyrrhizinate, vitamin C and other treatments, combined with oral administration of raw rhubarb powder. | Oral administration of potassium magnesium aspartate tablets on the basis of treatment with diammonium glycyrrhizinate, vitamin C, etc. | 1 month | The total effective rate of the observation group was significantly higher than that of the control group (P<0.05). The serum ALT, TB, and Fas/FasL levels in the observation group decreased significantly compared to the control group (P<0.05). | (Huang et al., 2008) |
